# Supplementary material for: Neoadjuvant cobimetinib and atezolizumab with or without vemurafenib for high-risk operable Stage III melanoma: the Phase II NeoACTIVATE trial
Source: Nat Commun. 2024 Feb 16;15:1430. doi: 10.1038/s41467-024-45798-8 (PMC10873383; doi:10.1038/s41467-024-45798-8)
Supplement: Supplementary file 1 — Supplementary Information [file 41467_2024_45798_MOESM1_ESM.pdf]

Neoadjuvant Cobimetinib and Atezolizumab With or Without Vemurafenib for High-Risk Operable Stage III Melanoma: the phase 2 NeoACTIVATE trial

Supplementary Information

Supplementary Table 1. Details of Pathologic Response

**Details of Pathologic Response**	Cohort A *BRAF*m N=15	Cohort B *BRAF*wt N=13	All Patients N=28
Number of lymph nodes examined, median (range)	14 (3-66)	14 (2-30)	14 (2-66)
Number of positive nodes at operation			
0	10 (66.7%)	2 (15.4%)	12 (42.9%)
1	3 (20%)	5 (38.5%)	8 (28.6%)
2	2 (13.3%)	0	2 (7.1%)
≥3	0	6 (46.2%)	6 (21.4%)
Number of positive nodes at operation, median (range)*	1 (1-3)	3 (1-8)	1.5 (1-8)
Greatest linear extent of disease at operation, mm, median (range)*	9.8 (0.92-25.0)	10 (0.4-77.0)	9.9 (0.4-77.0)
Extranodal extension present*	2/5 (40%)	6/11 (54.5%)	8/16 (50%)

*among node-positive patients

Supplementary Table 2. Detailed Summary of Adverse Events (Regardless of Attribution) During Neoadjuvant Treatment

Adverse Event	Cohort A (*BRAF*m, N=15)			Cohort B (*BRAF*wt, N=15)		
	Grade 2	Grade 3	Grade 4	Grade 2	Grade 3	Grade 4
Number of patients in total with at least one adverse event	12 (80%)	11 (73.3%)	1 (6.7%)	12 (80%)	8 (53.3%)	1 (6.7%)
Hypertension		2 (13.33%)			2 (13.33%)	1 (6.67%)
Hyperglycemia	1 (6.67%)	1 (6.67%)	1 (6.67%)			
Alanine aminotransferase increased	3 (20.00%)				3 (20.00%)	
Infections and infestations - Oth spec					2 (13.33%)	
Anaphylaxis					1 (6.67%)	
Hypercalcemia					1 (6.67%)	
Rash maculo-papular	2 (13.33%)	8 (53.33%)		3 (20.00%)		
Skin and subcut tissue disord - Oth spec	1 (6.67%)	2 (13.33%)				
Pneumonitis	1 (6.67%)	1 (6.67%)				
Photosensitivity	1 (6.67%)	1 (6.67%)				
CPK increased		1 (6.67%)		2 (13.33%)		
Hyponatremia		1 (6.67%)				
Hypoxia		1 (6.67%)				
Pruritus		1 (6.67%)				
Fatigue	5 (33.33%)			2 (13.33%)		
Arthralgia	4 (26.67%)					
Aspartate aminotransferase increased	3 (20.00%)			1 (6.67%)		
Fever	3 (20.00%)					
Nausea	3 (20.00%)			1 (6.67%)		
Lymphocyte count decreased	2 (13.33%)			1 (6.67%)		
Blood bilirubin increased	2 (13.33%)					
Myalgia	2 (13.33%)					
Dehydration	2 (13.33%)			2 (13.33%)		
Musculoskeletal, conn tissue - Oth spec	1 (6.67%)					
Sore throat	1 (6.67%)					
Thromboembolic event	1 (6.67%)					
Generalized muscle weakness	1 (6.67%)					
Acute kidney injury	1 (6.67%)					
Anemia	1 (6.67%)			1 (6.67%)		
Neutrophil count decreased	1 (6.67%)					
White blood cell decreased	1 (6.67%)			1 (6.67%)		
Alkaline phosphatase increased	1 (6.67%)					
Hypokalemia	1 (6.67%)			1 (6.67%)		
Hypophosphatemia	1 (6.67%)					
Headache	1 (6.67%)					
Dyspnea	1 (6.67%)			1 (6.67%)		
Anorexia	1 (6.67%)					
Diarrhea	1 (6.67%)			2 (13.33%)		
Mucositis oral	1 (6.67%)					
Allergic reaction				2 (13.33%)		
Edema face				2 (13.33%)		
Infusion related reaction				1 (6.67%)		
Platelet count decreased				1 (6.67%)		
Hyperkalemia				1 (6.67%)		
Back pain				1 (6.67%)		
Hypotension				1 (6.67%)		
Rash acneiform				1 (6.67%)		
Dry mouth				1 (6.67%)		

Supplementary Table 3. Detailed Summary of Adverse Events At Least Possibly Related to Neoadjuvant Treatment

Adverse Event	Cohort A (*BRAF*m, N=15)			Cohort B (*BRAF*wt, N=15)		
	Grade 2	Grade 3	Grade 4	Grade 2	Grade 3	Grade 4
Number of patients in total with at least one adverse event	12 (80%)	11 (73.3%)	1 (6.7%)	11 (73.3%)	3 (20%)	0 (0%)
Hyperglycemia		1 (6.67%)	1 (6.67%)			
Rash maculo-papular	2 (13.33%)	8 (53.33%)		3 (20.00%)		
Skin and subcut tissue disord - Oth spec	1 (6.67%)	2 (13.33%)				
Pneumonitis	1 (6.67%)	1 (6.67%)				
Photosensitivity	1 (6.67%)	1 (6.67%)				
CPK increased		1 (6.67%)		1 (6.67%)		
Hyponatremia		1 (6.67%)				
Hypoxia		1 (6.67%)				
Pruritus		1 (6.67%)				
Alanine aminotransferase increased	3 (20.00%)				2 (13.33%)	
Anaphylaxis					1 (6.67%)	
Fatigue	5 (33.33%)			2 (13.33%)		
Arthralgia	4 (26.67%)					
Aspartate aminotransferase increased	3 (20.00%)			1 (6.67%)		
Fever	3 (20.00%)					
Nausea	3 (20.00%)			1 (6.67%)		
Lymphocyte count decreased	2 (13.33%)			1 (6.67%)		
Myalgia	2 (13.33%)					
Dehydration	2 (13.33%)			2 (13.33%)		
Musculoskeletal, conn tissue - Oth spec	1 (6.67%)					
Sore throat	1 (6.67%)					
Thromboembolic event	1 (6.67%)					
Generalized muscle weakness	1 (6.67%)					
Acute kidney injury	1 (6.67%)					
Anemia	1 (6.67%)					
Neutrophil count decreased	1 (6.67%)					
White blood cell decreased	1 (6.67%)			1 (6.67%)		
Alkaline phosphatase increased	1 (6.67%)					
Hypokalemia	1 (6.67%)					
Headache	1 (6.67%)					
Dyspnea	1 (6.67%)			1 (6.67%)		
Anorexia	1 (6.67%)					
Diarrhea	1 (6.67%)			2 (13.33%)		
Mucositis oral	1 (6.67%)					
Anorexia	1 (6.67%)					
Diarrhea	1 (6.67%)			2 (13.33%)		
Allergic reaction				2 (13.33%)		
Edema face				2 (13.33%)		
Infusion related reaction				1 (6.67%)		
Platelet count decreased				1 (6.67%)		
Hyperkalemia				1 (6.67%)		
Hypotension				1 (6.67%)		
Rash acneiform				1 (6.67%)		
Dry mouth				1 (6.67%)		

Supplementary Table 4. 60 Day Postoperative Complications*

	N	Complication	
		Surgical Site Infection	Seroma
**All Patients**	28**	2 (7.1%)	6 (21.4%)
**Anatomic Basin**			
Cervical	6	0	0
Axillary	10	0	2 (7.1%)
Inguinal +/- Pelvic	13	2 (7.1%)	4 (14.2%)

*All complications were Grade 2

**Total number of anatomic basins = 29, as one patient had both a cervical and axillary dissection

Supplementary Table 5. Flow cytometry antibody panel*.

Name	Clone	Cat#	Concentration (dilution)	Company
Anti-Human CD8-PE-Cy7	RPA-T8	# 557746	1:200	BD Biosciences
Anti-human PD-1-BV510	EH12.2H7	#329931	1:100	Biolegend
Anti-human CD11a-APC	HI111	301212	1:200	Biolegend
Anti-human CX3CR1-APC/Cy7	2A9.1	341616	1:100	Biolegend
Anti-human CD3-BV421	OKT3	317344	1:200	BD Biosciences
Anti-Bim-Rabbit mAb -PE	C34C5	12186	1:200	Cell Signaling
Anti-NKG7- Rabbit mAb -AF488	8H3/8K3		1:200	House made

*Antibodies were validated in previously published studies as referenced below.

1. Yan, Y., et al. CX3CR1 identifies PD-1 therapy-responsive CD8+ T cells that withstand chemotherapy during cancer chemoimmunotherapy. JCI Insight 3(2018).

2. Zhang, H., et al. Phase II Evaluation of Stereotactic Ablative Radiotherapy (SABR) and Immunity in (11)C-Choline-PET/CT-Identified Oligometastatic Castration-Resistant Prostate Cancer. Clin Cancer Res 27, 6376-6383 (2021).

3. Gicobi, J.K., et al. Salvage therapy expands highly cytotoxic and metabolically fit resilient CD8(+) T cells via ME1 up-regulation. Sci Adv 9, eadi2414 (2023).

4. Wen, T., et al. NKG7 Is a T-cell-Intrinsic Therapeutic Target for Improving Antitumor Cytotoxicity and Cancer Immunotherapy. Cancer Immunol Res 10, 162-181 (2022).

Supplementary Table 6. Mass Cytometry Antibody Panel*.

No.	Target	Metal	Clone	Vendor	Catalog Number	Dilution Factor	Surface or Intracellular	Cell Specificity
1	CD45	089Y	HI30	Standard BioTools	3089003B	400	Surface	Lymphocytes
2	CD196/CCR6	141Pr	G034E3	Standard BioTools	3141003A	200	Surface	Effector memory T-cells
3	CD19	142Nd	HIB19	Standard BioTools	3142001B	400	Surface	B-cells
4	CD127/IL-7Ra	143Nd	A019D5	Standard BioTools	3143012B	200	Surface	Effector memory T-cells
5	CD38	144Nd	HIT2	Standard BioTools	3144014B	200	Surface	Basophils
6	CD11a	145Nd	HI111	Biolegend	301223	400	Surface	T-cell effector
7	IgD	146Nd	IA6-2	Standard BioTools	3146005B	400	Surface	Naïve B-cells
8	CD11c	147Sm	Bu15	Standard BioTools	3147008B	400	Surface	monocytic DC
9	CD16	148Nd	3G8	Standard BioTools	3148004B	800	Surface	NK Cells
10	CD194/CCR4	149Sm	L291h4	Standard BioTools	3149029A	200	Surface	T-regulatory cells/Th2 cells
11	LAG-3	150Nd	11C3C65	Standard BioTools	3150030B	100	Surface	T-cell exhaustion
12	CD123/IL-3R	151Eu	6H6	Standard BioTools	3151001B	200	Surface	Plasmacytoid DC/Basophils
13	TCRgd	152Sm	11F2	Standard BioTools	3152008B	400	Surface	Gamma/Delta T-cells
14	CD185/CXCR5	153Eu	RF8B2	Standard BioTools	3153020B	200	Surface	Central Memory/Effector Memory
15	CD3	154Sm	UCHT1	Standard BioTools	3154003B	400	Surface	T-cells
16	CD45RA	155Gd	HI100	Standard BioTools	3155011B	400	Surface	Naïve T and Memory B cells
17	PD-L1	156Gd	29E.2A3	Standard BioTools	3156026B	100	Surface	MDSC/T-cell exhaustion
18	CD27	158Gd	L128	Standard BioTools	3158010B	400	Surface	Memory B-cells
19	Tim-3	159Tb	F38-2E2	Biolegend	345019	100	Surface	T-cell exhaustion
20	CD28	160Gd	CD28.2	Standard BioTools	3160003B	200	Surface	Naïve CD8 T cells
21	PD-1	161Dy	EH12.2H7	Biolegend	329941	100	Surface	T-cell exhaustion
22	CD66b	162Dy	80H3	Standard BioTools	3162023B	100	Surface	Neutrophils
23	CD183/CXCR3	163Dy	G025H7	Standard BioTools	3163004B	200	Surface	Th1 cells
24	CD161	164Dy	HP-3G10	Standard BioTools	3164009B	100	Surface	MAIT and NKT cells
25	CD45RO	165Ho	UCHL1	Standard BioTools	3165011B	200	Surface	Activated/Memory T-cells
26	CD24	166Er	ML5	Standard BioTools	3166007B	200	Surface	Memory B-cells
27	CD197/CCR7	167Er	G043H7	Standard BioTools	3167009A	200	Surface	Naïve/Central memory CD8 T cells
28	CD8a	168Er	SK1	Standard BioTools	3168002B	800	Surface	CD8 T-cells
29	CD25/IL-2R	169Tm	2A3	Standard BioTools	3169003B	200	Surface	Activated T-cells; T-regulatory cells
30	CTLA-4	170Er	14D3	Standard BioTools	3170005B	100	Surface	T-cell exhaustion
31	CD20	171Yb	2H7	Standard BioTools	3171012B	800	Surface	B-cells
32	CX3CR1	172Yb	2A9-1	Biolegend	341602	100	Surface	Effector/Memory T-cells
33	HLA-DR	173Yb	L243	Standard BioTools	3173005B	400	Surface	monocytic DC

34	CD4	174Yb	SK3	Standard BioTools	3174004B	800	Surface	CD4 T-cells/Monocytes
35	CD14	175Lu	M5E2	Standard BioTools	3175015B	200	Surface	Monocytes
36	CD56/NCAM	176Yb	NCAM16.2	Standard BioTools	3176008B	200	Surface	NK Cells
37	TIGIT	209Bi	MBSA43	Standard BioTools	3209013B	100	Surface	Immune checkpoint; Exhaustion

*Antibody validation was performed by the vendors per established protocols.

Supplementary Figure 1. Histologic Details of Pathologic Response.

The percentages of the tumor bed area at the time of operation represented by viable tumor, melanosis/necrosis, and fibrosis are shown. Each column represents an individual patient. (Cohort A: *BRAF*m, Cohort B: *BRAF*wt)

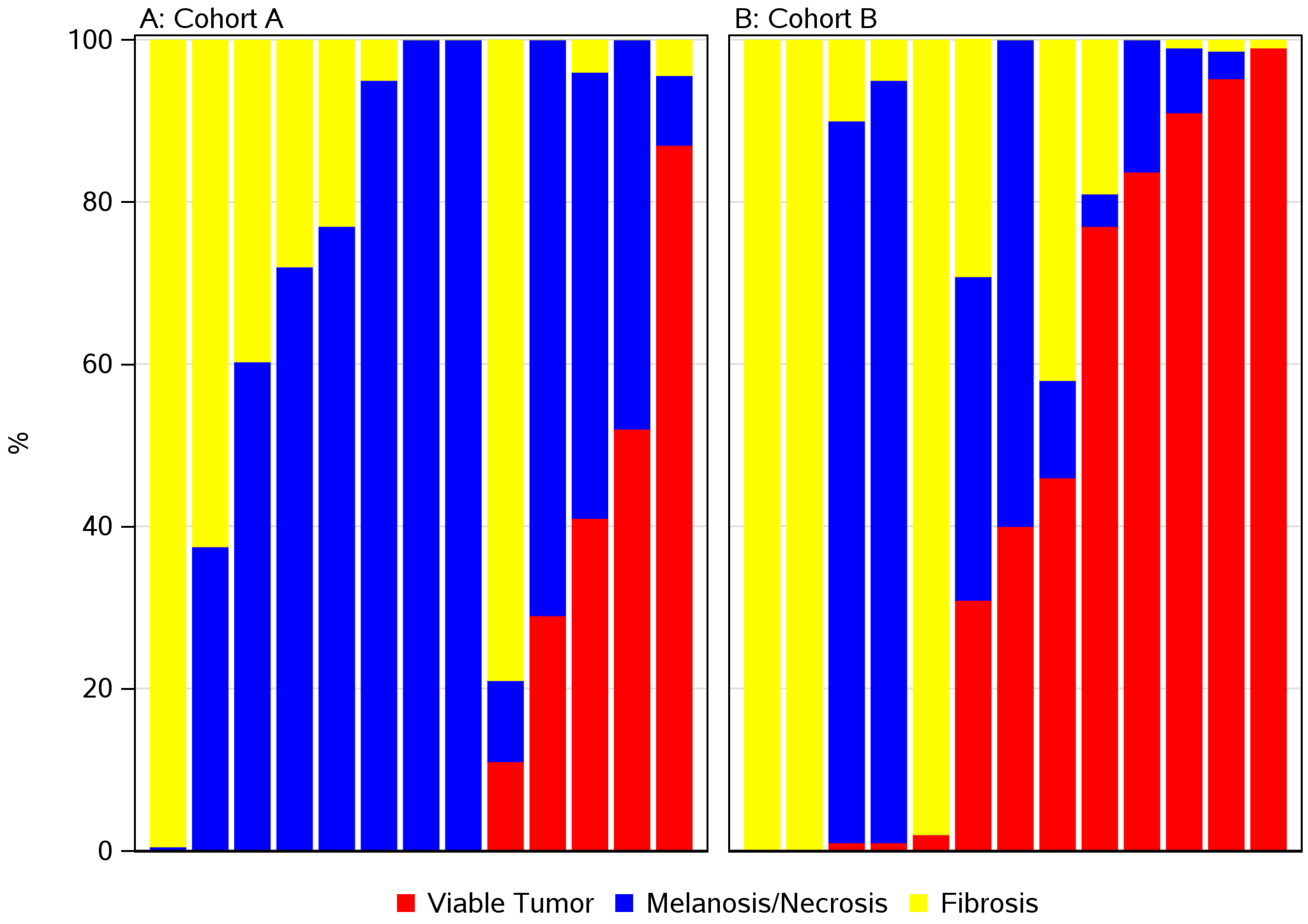

Supplementary Figure 2. PD-L1 IHC and sPD-L1 Analyses in Cohort A (*BRAF*m).

The percentage of tumor cells (A) and immune cells (B) with PD-L1 expression at baseline, and the change in percentage of immune cells with PD-L1 expression after neoadjuvant treatment (C) were quantitated. The plasma concentration of soluble PD-L1 at baseline (D) and the change in sPD-L1 concentration (E) were measured. Tissue PD-L1 staining on tumor (F) and immune cells (G, H) was compared at baseline (F, G) and after neoadjuvant therapy (H). Blue circles are ≤ 10% viable tumor. Red triangles are > 10% viable tumors. A-E lines are medians.

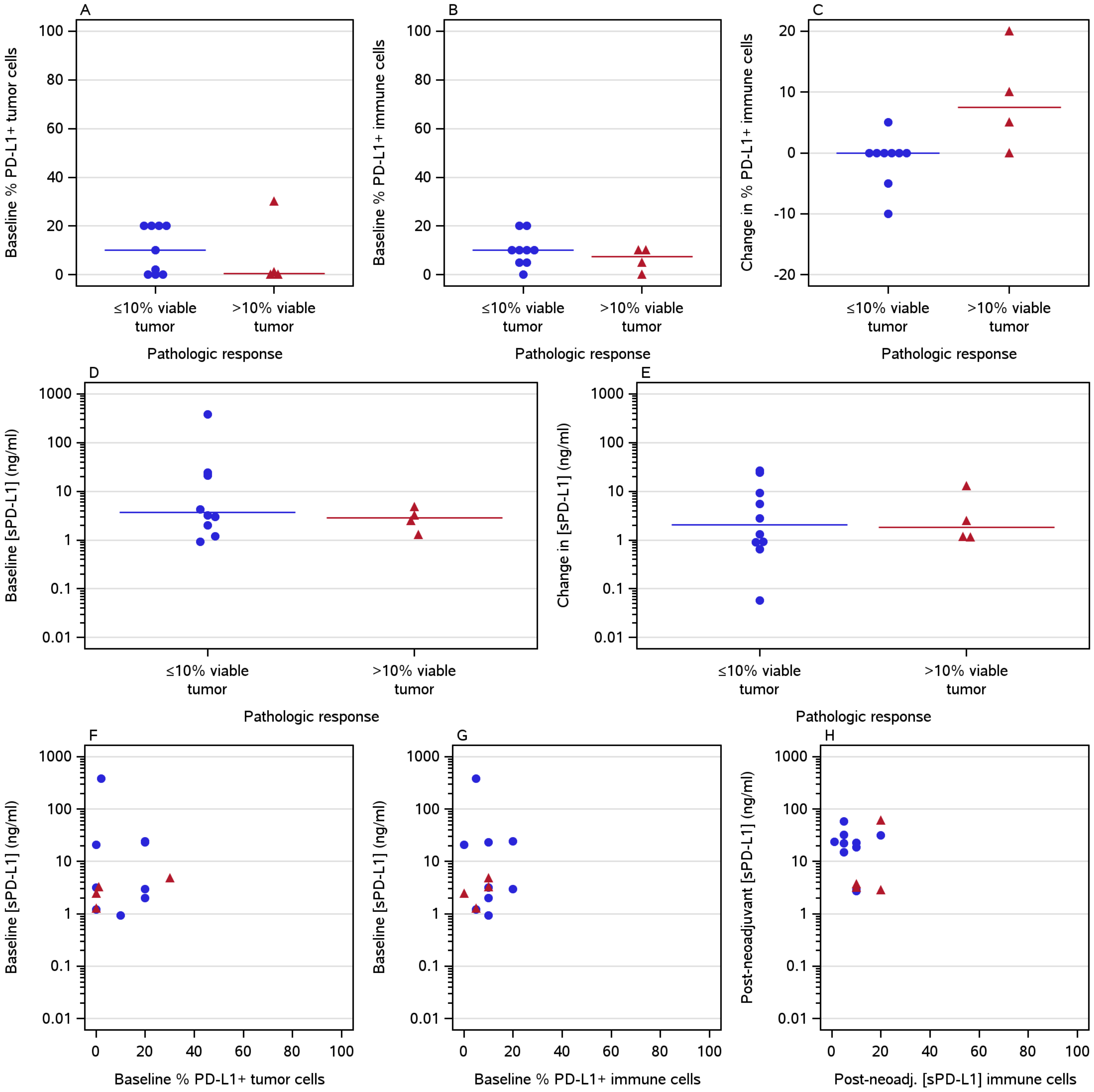

Supplementary Figure 3. PD-L1 IHC and sPD-L1 Analyses in Cohort B (*BRAF*wt).

The percentage of tumor cells (A) and immune cells (B) with PD-L1 expression at baseline, and the change in percentage of immune cells with PD-L1 expression after neoadjuvant treatment (C) were quantitated. The plasma concentration of soluble PD-L1 at baseline (D) and the change in sPD-L1 concentration (E) were measured. Tissue PD-L1 staining on tumor (F) and immune cells (G, H) was compared at baseline (F, G) and after neoadjuvant therapy (H). Blue circles are ≤ 10% viable tumor. Red triangles are > 10% viable tumors. A-E lines are medians.

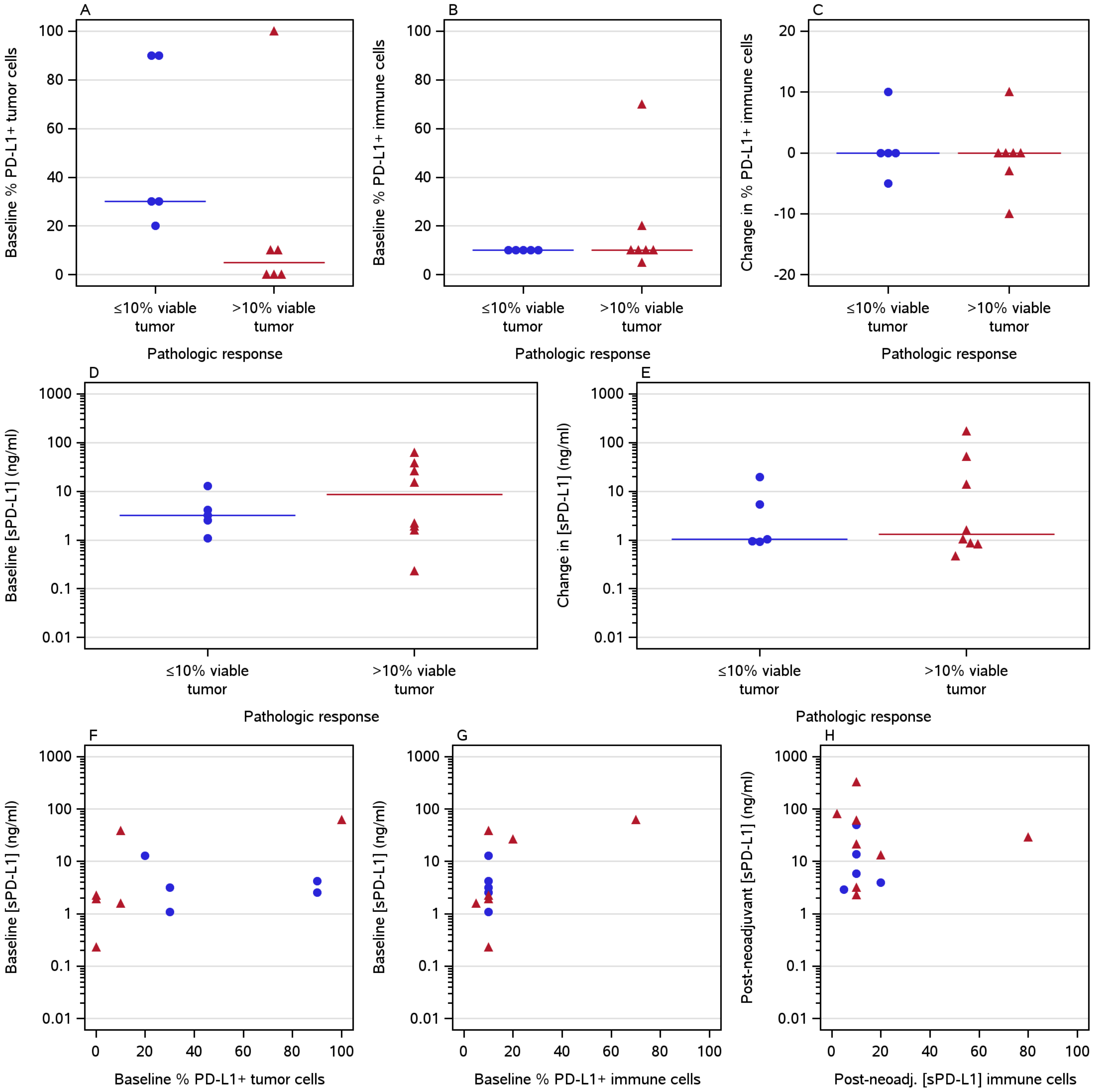

Supplementary Figure 4. Differential Abundance of Immune Cell Subsets Over Time.

Panel A shows volcano plots summarizing the results of differential abundance analyses of immune cell subsets comparing time points for the *BRAF*m (top) and *BRAF*wt (bottom) cohorts separately. The linear mixed effects model is used. The X-axis shows the log-fold changes between time points with the reference time point ("ref") indicated. The Y-axis shows the log of FDR-adjusted p-values. Cell subsets with FDR level ≤ 0.2 are indicated in red. Panel B shows changes in abundance of individual cell types identified as significant in Panel A for *BRAF*wt patients: naïve CD4+ T cells, CD4+ T_{CM}, and CD4+ T_{EM}.

A

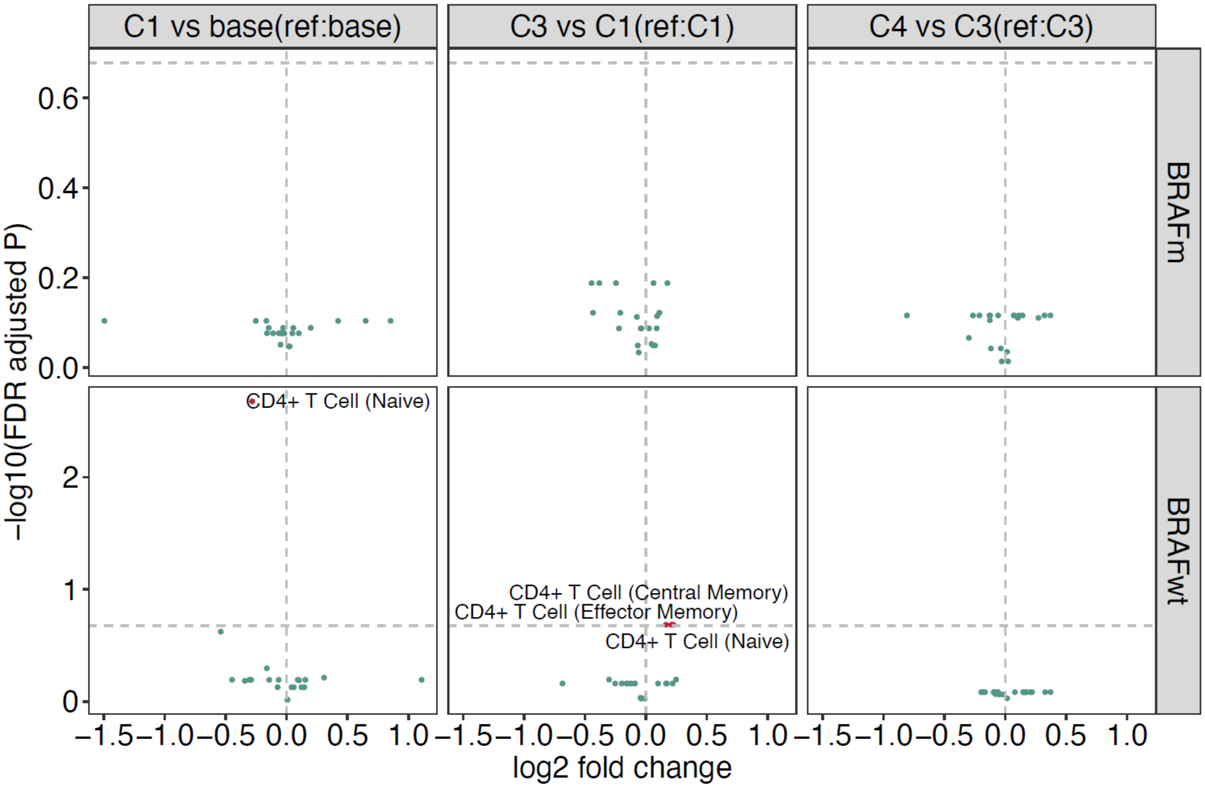

B

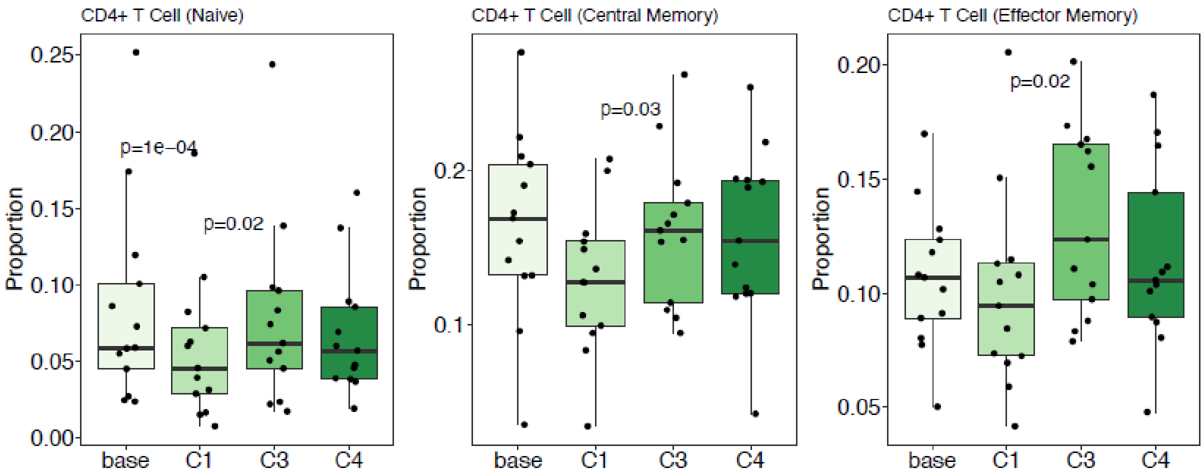

Supplementary Figure 5. Flow Cytometry Analyses in Relation to Response to Neoadjuvant Therapy.

Tumor-related T cells, effector CTLs, and pro-apoptotic T cells were quanitated at baseline **(A, B, C)**, and the difference in percentage from baseline to post-neoadjuvant treatment was assessed **(D, E, F)**. Tumor-related T cells are expressed as a percentage of CD8+ T cells **(A, D)**, while effector CTLs and pro-apoptotic T cells are expressed as a percentage of tumor-related T cells **(B, C, E, F)**. Pooled data from both cohorts are shown here. Data from individual cohorts are shown in Supplementary Figures 6 (Cohort A, *BRAF*m) and 7 (Cohort B, *BRAF*wt). Blue circles are ≤ 10% viable tumor. Red triangles are > 10% viable tumors. Lines are medians. The gating strategy is shown in Supplementary Figure 8.

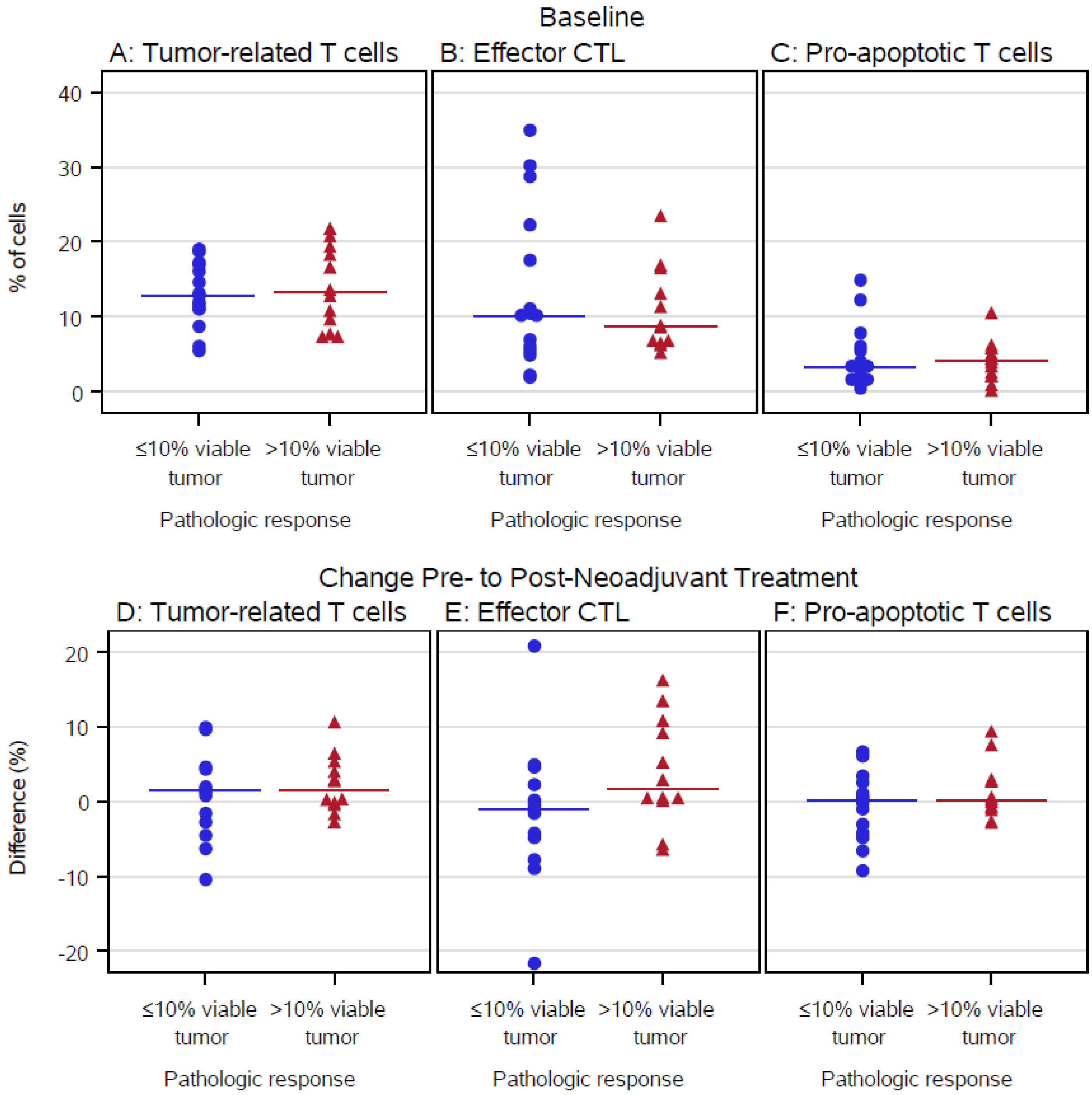

Supplementary Figure 6. Flow Cytometry Analyses in Relation to Response to Neoadjuvant Therapy, Cohort A, *BRAF*m.

Tumor-related T cells, effector CTLs, and pro-apoptotic T cells were quanitated at baseline **(A, B, C)**, and the difference in percentage from baseline to post-neoadjuvant treatment was assessed **(D, E, F)**. Tumor-related T cells are expressed as a percentage of CD8+ T cells **(A, D)**, while effector CTLs and pro-apoptotic T cells are expressed as a percentage of tumor-related T cells **(B, C, E, F)**. Blue circles are ≤ 10% viable tumor. Red triangles are > 10% viable tumors. Lines are medians. The gating strategy is shown in Supplementary Figure 8.

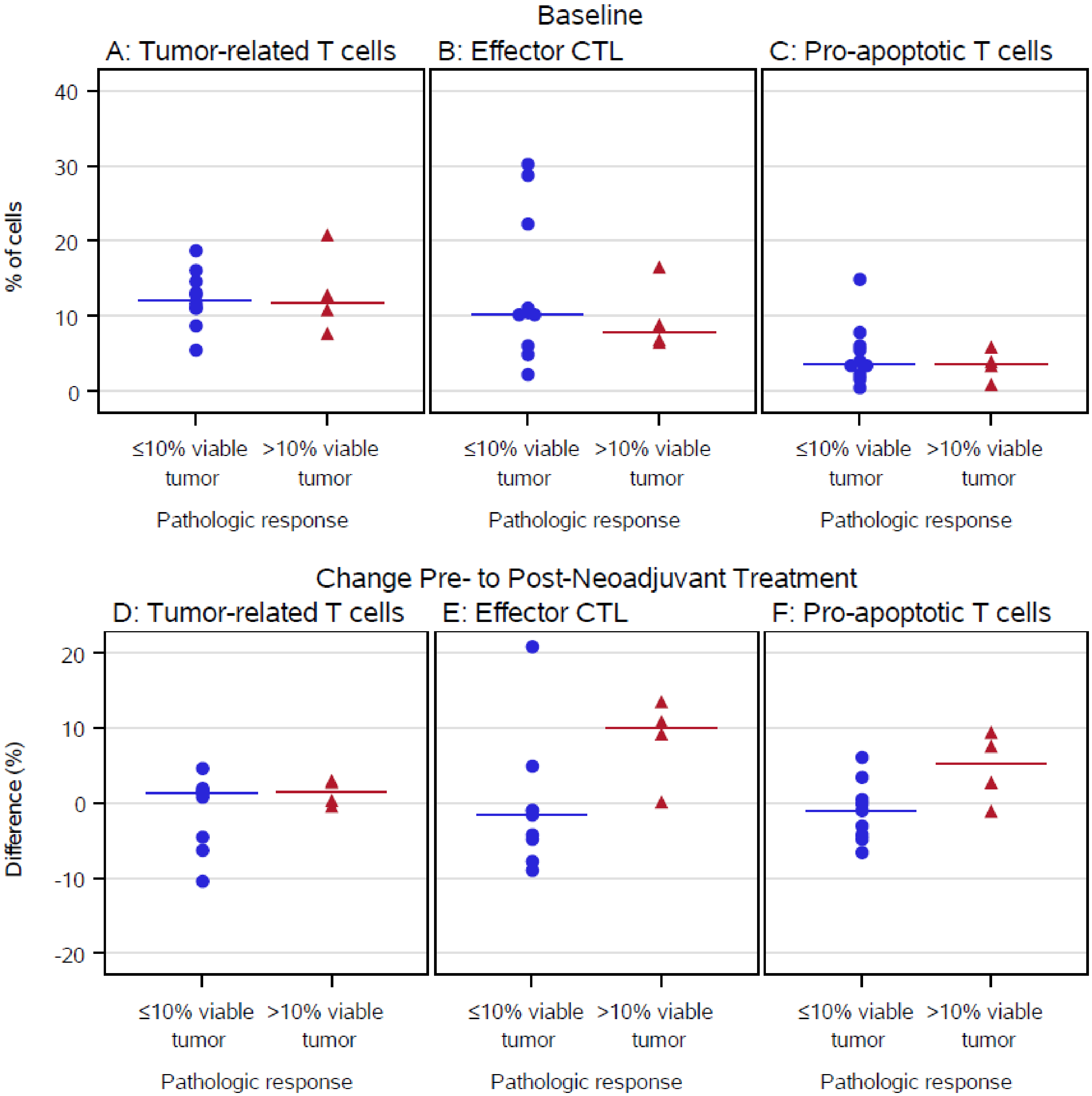

Supplementary Figure 7. Flow Cytometry Analyses in Relation to Response to Neoadjuvant Therapy, Cohort B, *BRAF*wt.

Tumor-related T cells, effector CTLs, and pro-apoptotic T cells were quanitated at baseline **(A, B, C)**, and the difference in percentage from baseline to post-neoadjuvant treatment was assessed **(D, E, F)**. Tumor-related T cells are expressed as a percentage of CD8+ T cells **(A, D)**, while effector CTLs and pro-apoptotic T cells are expressed as a percentage of tumor-related T cells **(B, C, E, F)**. Blue circles are ≤ 10% viable tumor. Red triangles are > 10% viable tumors. Lines are medians. The gating strategy is shown in Supplementary Figure 8.

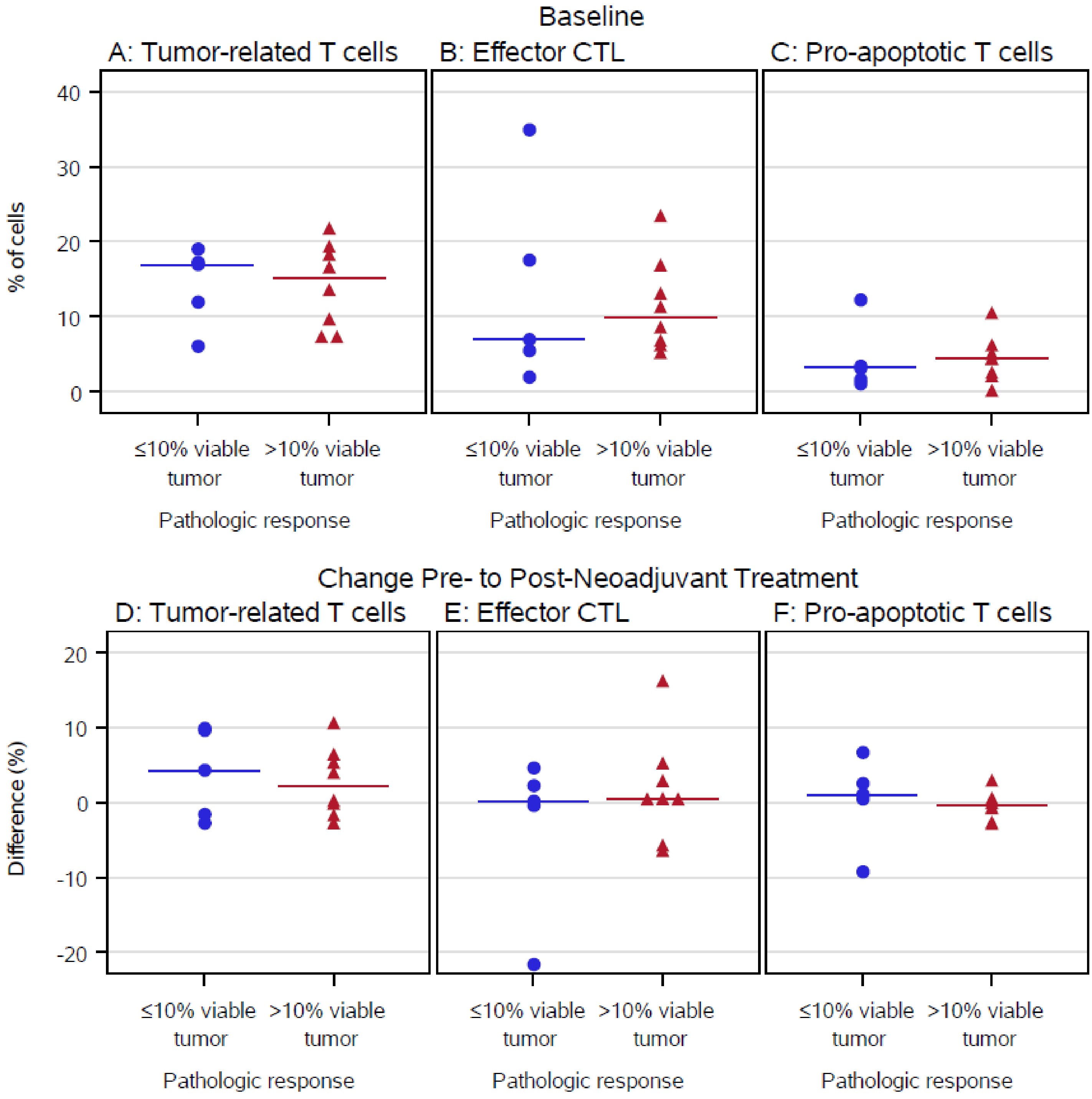

Supplementary Figure 8. Flow cytometry gating strategy for Supplementary Figures 5-7

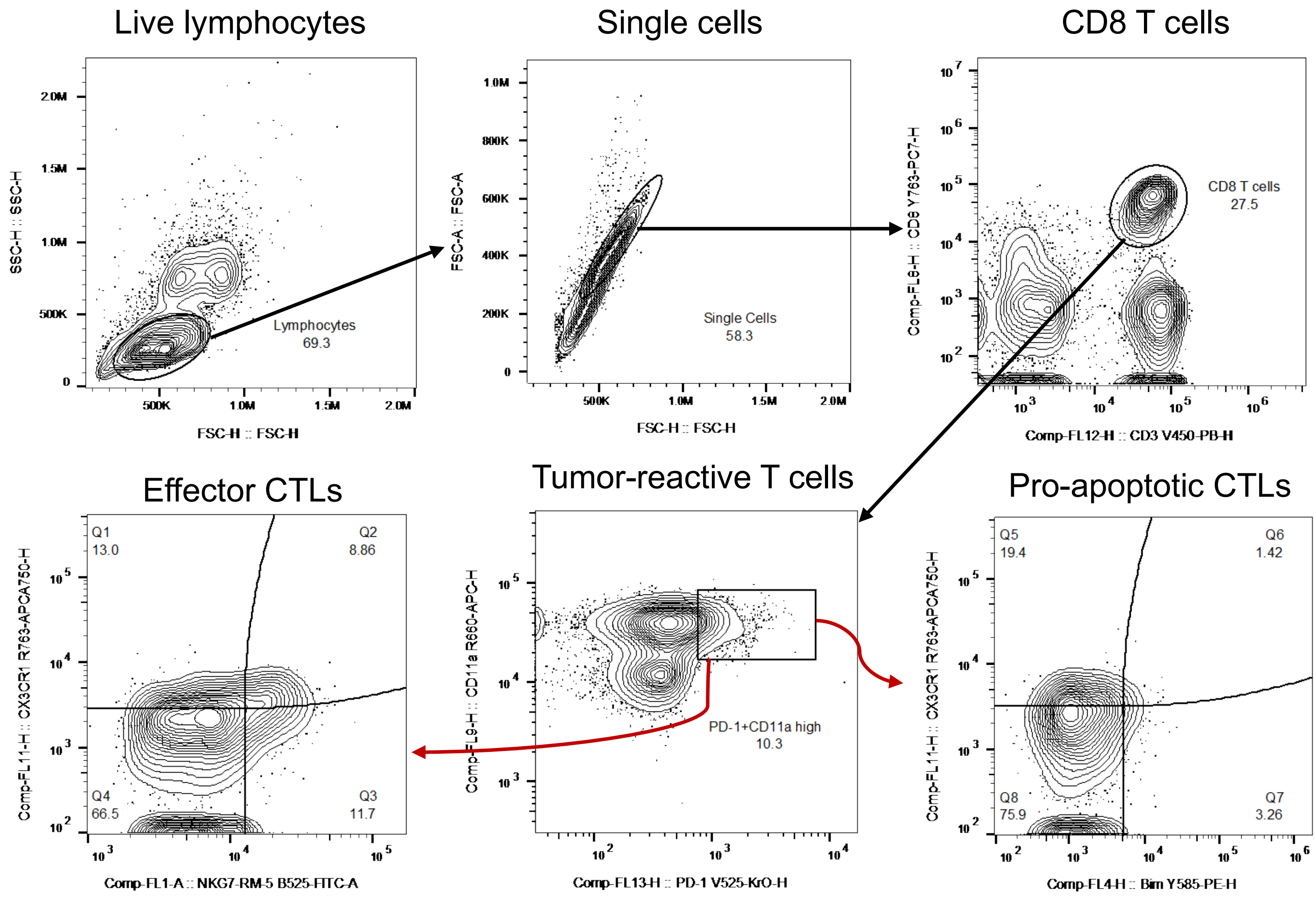

Supplementary Note 1 (Study Protocol)

Mayo Clinic Cancer Center

Neoadjuvant therapy for patients with high risk stage III melanoma: a pilot clinical trial

Study Chairs: Matthew S Block MD PhD
Tina J Hieken MD

Mayo Clinic
200 First Street SW
Rochester MN 55905
507/284-2511

Study Cochairs: Evidio Domingo Musibay MD (University of Minnesota)
Thomas J Flotte MD√
Yiyi Yan MD PhD
Roxana S Dronca MD
Lisa A Kottschade APRN CNP
Haidong Dong MD PhD√

Statistician: Vera J Suman PhD√

√Study contributor(s) not responsible for patient care

Drug Availability
Supplied Investigational Agents: Atezolizumab, Cobimetinib, Vemurafenib (IND#137509)

Drug Company Supplied: Atezolizumab, Cobimetinib, Vemurafenib

Document History	Effective Date
Activation	22Jun2018
MCCC Amendment 1	20Feb2019
MCCC Amendment 2	25Jul2019
MCCC Amendment 3	09Oct2019
MCCC Amendment 4	pending

Protocol Resources

Questions:	Contact Name:
Patient eligibility*, test schedule, treatment delays/interruptions/adjustments, dose modifications, adverse events, forms completion and submission	Sharon F Solinger<br>Quality Assurance Specialist<br>Phone: 507/266-1714<br>E-mail: solinger.sharon@mayo.edu
Drug administration, infusion pumps, nursing guidelines	Lisa A Kottschade APRN CNP<br>Phone: 507/538-7888<br>E-mail: kottschade.lisa@mayo.edu
Clinical Research Coordinator (lead site)	Heidi J Turner<br>Clinical Research Coordinator<br>Phone: 507/266-3172<br>Email: turner.heidi@mayo.edu
Clinical Data Coordinator (lead site)	Tracy L Callahan<br>Clinical Data Coordinator<br>Phone: 507/538-6739<br>Email: callahan.tracy@mayo.edu
Melanoma Program Coordinator	Jill M Schimke MS<br>Melanoma Program Coordinator<br>Phone: 507/266-0743<br>Email: schimke.jill@mayo.edu
Protocol document, consent form, regulatory issues (lead site)	Lynn M Flickinger MA<br>Senior Research Protocol Specialist<br>Phone: 507/538-7034<br>Email: flickinger.lynn@mayo.edu<br><br>Mitchell (Mitch) G Perrizo<br>Research Protocol Specialist<br>Phone: 507/284-9104<br>Email: perrizo.mitchell@mayo.edu
Laboratory Contacts for Correlative Studies	Courtney L Erskine<br>Immunology Lab<br>Phone: 507/284-4488{Pager 127-14057}<br>Email: erskine.courtney@mayo.edu
Serious Adverse Events	Patricia (Pat) G McNamara<br>SAE Coordinator<br>Phone: 507/266-3028<br>E-mail: mcnamara.patricia@mayo.edu

*No waivers of eligibility allowed

Table of Contents

Neoadjuvant therapy for patients with high risk stage III melanoma: a pilot clinical trial 1
Protocol Resources 2
Table of Contents 3
Schema for Arms A and B 4
Schema for Arm C (BRAF wild type or BRAF mutant Melanoma) 5
1.0 Background 6
2.0 Goals 15
3.0 Patient Eligibility 17
4.0 Study Schedules 22
5.0 Grouping Factor 27
6.0 Registration/Randomization Procedures 27
7.0 Protocol Treatment 31
8.0 Dosage Modification Based on Adverse Events 33
9.0 Ancillary Treatment/Supportive Care 50
10.0 Adverse Event (AE) Monitoring and Reporting 59
11.0 Treatment Evaluation 79
12.0 Descriptive Factors 79
13.0 Treatment/Follow–up Decision at Evaluation of Patient 79
14.0 Body Fluid Biospecimens 81
15.0 Drug Information 85
16.0 Statistical Considerations and Methodology 95
17.0 Pathology Considerations/Tissue Biospecimens 102
18.0 Records and Data Collection Procedures 105
19.0 Budget 105
20.0 References 106
Appendix I ECOG Performance Status 111
Appendix II MC1776 Patient Medication Diary 112
Appendix III General Guidelines for Concomitant Therapy and Additional Restrictions* 114

Schema for Arms A and B

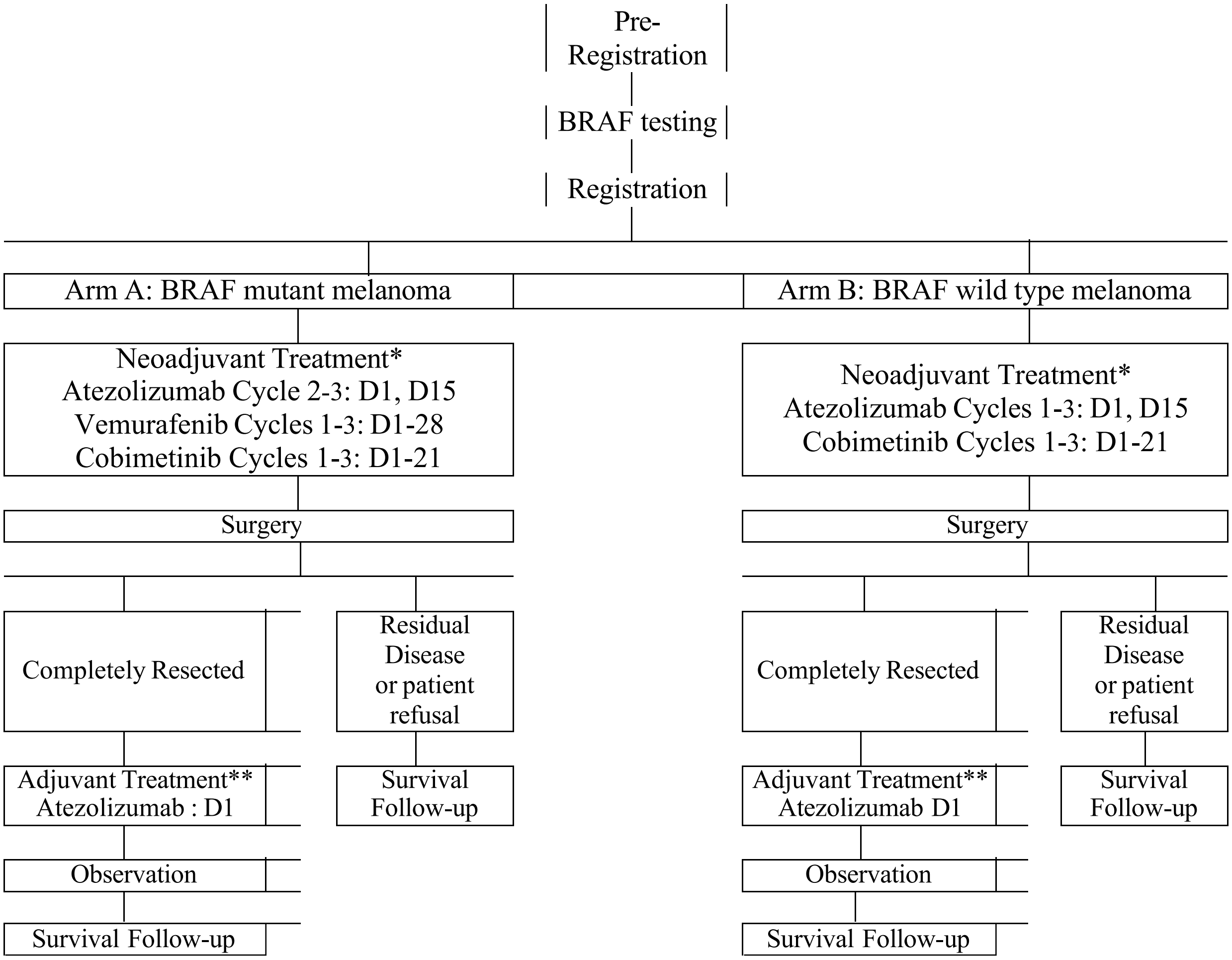

*Cycle neoadjuvant = 28 days ±3 days (maximum of 3 cycles of treatment)

Cycle surgery = ~4-6 weeks

**Cycle adjuvant = 21 days ±3 days (maximum of 8 cycles of treatment)

Total of 30 patients with 15 patients in each arm

Patients who do not have surgery will proceed to Survival Follow-up

If patient has disease progression confirmed by imaging during Neo-adjuvant treatment that no longer deems them a surgical candidate, the patient proceeds to survival follow-up.

Generic name: Atezolizumab<br>Brand name(s): Tecentriq®<br>Mayo Abbreviation: ATEZO<br>Availability: Genentech	Generic name: Cobimetinib<br>Brand name(s):Cotellic®<br>Mayo Abbreviation: COBI<br>Availability: Genentech	Generic name: Vemurafenib<br>Brand name(s): Zelboraf®<br>Mayo Abbreviation: VEM<br>Availability: Genentech

Schema for Arm C (BRAF wild type or BRAF mutant Melanoma)

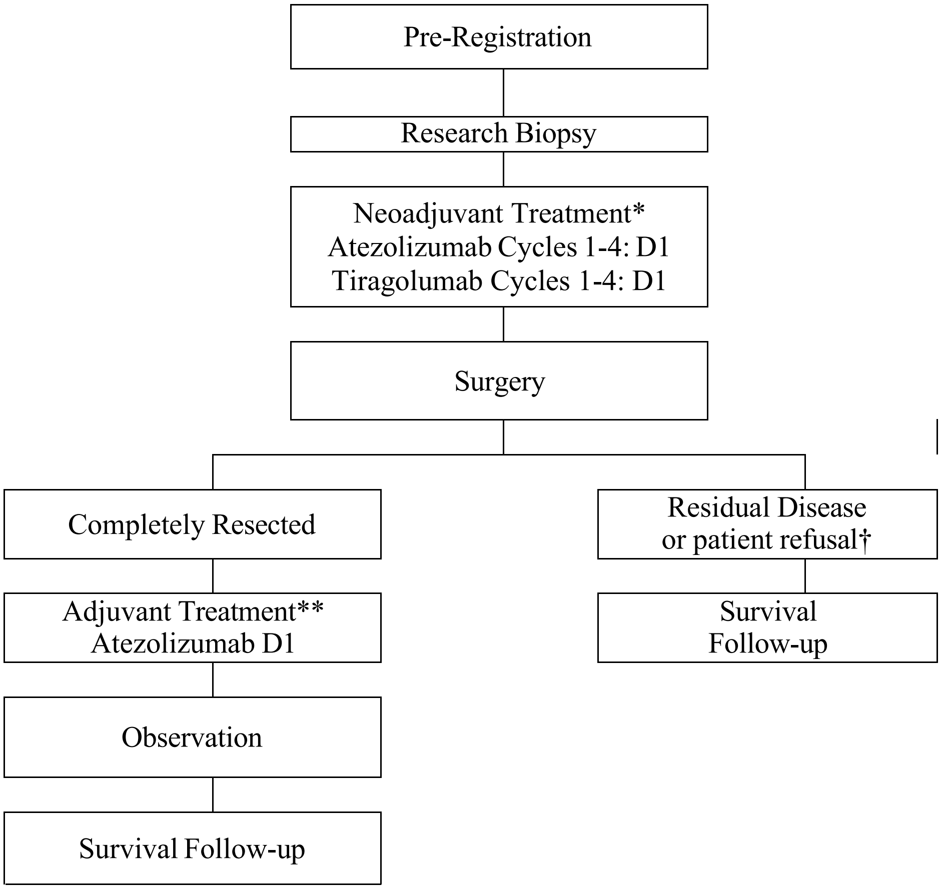

*Cycle neoadjuvant = 21 days ±3 days (maximum of 4 cycles of treatment)

Surgery = ~4-6 weeks

**Cycle adjuvant = 21 days ±3 days (maximum of 8 cycles of treatment)

†Patients who do not have surgery will proceed to Survival Follow-up
If patient has disease progression confirmed by imaging during neo-adjuvant treatment that no longer deems them a surgical candidate, the patient proceeds to survival follow-up.

Total of 36 patients (maximum)

Generic name: Atezolizumab<br>Brand name(s): Tecentriq®<br>Mayo Abbreviation: ATEZO<br>Availability: Genentech	Generic name: Tiragolumab<br>Brand name(s):<br>Mayo Abbreviation: TIRA<br>Availability: Genentech

1.0 Background

1.1 Stage III Melanoma

The rapid and continuously rising incidence of invasive melanoma over the past several decades, with an estimated 87,110 new cases in 2017 in the US alone, translates into a ten-fold increase in the number of patients with stage III disease at presentation and expands in parallel the number at risk of regional nodal relapse (Silverberg 1973; Siegel 2017; Guy 2015; Svedman 2016). Surgery remains the primary treatment for the majority of patients with melanoma. While surgery is most often curative for patients with early stage melanoma, patients with advanced stage III disease, including those who have clinically evident nodal disease, involvement of multiple nodal basins or recurrence in a regional lymph node basin, have a very high risk of subsequent recurrence even after successful and apparently complete surgical eradication of disease (Balch 2010; Morton 2014). Reported five-year overall survival for these patients ranges from 29% to 52% while 5-year progression-free survival is approximately 10 to 35% (Romano 2010).

1.2 Adjuvant Therapy for Stage III Melanoma

The approved adjuvant therapies for stage III melanoma, interferon alpha and ipilimumab, are associated with considerable toxicity and provide a modest overall survival benefit; the median progression-free survival for patients with macroscopic nodal metastases treated with adjuvant ipilimumab is 15.4 months (Eggermont 2015). In addition, evidence from one randomized controlled trial has demonstrated efficacy of nivolumab (Weber 2017). Given their toxicity and modest clinical efficacy, current adjuvant therapies are not acceptable for many patients.

1.3 New Drug Classes Have Revolutionized Melanoma Care

Survival of patients with metastatic melanoma has been improved by two classes of drugs recently approved by the FDA: targeted therapies and immune checkpoint inhibitors (Shah 2014).

Targeted treatments provide a high response rate, rapid responses, and prolongation of overall survival for approximately 50% of melanoma patients whose tumors harbor a somatic mutation at BRAFV600 (hereafter BRAFm melanoma) (Davies 2002). Specifically, the combination of the BRAF inhibitor vemurafenib and the MEK inhibitor cobimetinib (hereafter vem/cobi) is associated with an objective response rate of 68% in patients with metastatic BRAFm melanoma (Larkin 2014).

In contrast to targeted therapies, immunotherapies appear to be effective independent of BRAF mutation status. While responses to immunotherapy may be less frequent than to targeted therapy, many responses to immunotherapy are durable. In addition to the approved melanoma therapies ipilimumab, nivolumab, and pembrolizumab, the programmed death ligand 1 (PD-L1) inhibitor atezolizumab (hereafter atezo) recently has demonstrated activity in patients with pretreated urothelial carcinoma and non-small cell lung cancer(Rosenberg 2016; Fehrenbacher 2016).

Recently, the preliminary safety and clinical activity results of a trial testing the combination of atezo/vem/cobi has been reported for patients with metastatic BRAFm melanoma (Sullivan 2016) and updated in June 2017 (Sullivan 2017). No emerging toxicities beyond those expected for the vem/cobi combination and for atezolizumab were seen, although Grade 3-4 hepatotoxicity was noted in several patients (and was primarily attributed to vem/cobi). The objective response rate to vemurafenib/cobimetinib/atezolizumab was 85% (29 of 34 patients) including a complete response (CR) in 6 patients (21%) and durable responses in 20 patients (69%). For patients with BRAF-wild-type (hereafter BRAFwt) melanoma, effective targeted therapies have not been identified; as such, immune checkpoint inhibition vis-à-vis atezolizumab monotherapy is a reasonable approach, albeit with a lower anticipated response rate.

1.4 Rationale for Neoadjuvant Therapy for High-Risk Stage III Melanoma

In the landscape of effective immunotherapies, high-risk nodal disease might actually present a unique window of opportunity for the optimal delivery of immunotherapy, with or without targeted agents, generating an immune response to the tumor *in vivo* with the goal of thwarting progression to lethal metastatic disease. Effective neoadjuvant therapy for high risk melanoma would be advantageous in that it would both 1) reduce the risk for recurrence of melanoma after curative-intent surgery in patients with responsive disease, and 2) allow for early identification of the subset of patients with stage III disease who would be diagnosed with systemic metastasis shortly after operation with the surgery first approach, and thereby spare these patients a futile operation. In addition, if it induces a tumor response, neoadjuvant therapy may convert unresectable or borderline-resectable melanoma to resectable disease. Moreover, for patients with technically resectable yet bulky disease, a favorable response to upfront systemic therapy facilitates surgical resection of residual disease; data supports that lymphadenectomy for high-volume versus low volume disease is associated with greater long-term morbidity including lymphedema (Faries 2010) .Neoadjuvant therapy has become standard of care for several cancers, especially those with regional nodal involvement, and a pathologic complete response (pCR) to neoadjuvant therapy generally predicts an overall survival benefit (Donohoe 2017; Capirci 2008; Haddad 2015; Cortazar 2015). However, neoadjuvant therapies for Stage III melanoma have not been systematically studied. Thus, we believe neoadjuvant therapy may improve both cancer and quality of life outcomes for patients with high-risk stage III melanoma.

1.41 Preclinical data supporting the use of neoadjuvant therapy

There are preclinical data suggesting that neoadjuvant is more effective than adjuvant immunotherapy in preventing or eradicating distant metastases in mouse models of spontaneously metastasizing triple-negative breast cancer (Liu 2016). Further, these investigators show peripheral tumor-specific CD8+ T cells pre and post-treatment were predictive of response, suggesting potential biomarkers of response for human clinical investigation. In murine melanoma models neoadjuvant vaccination has been shown to be superior to adjuvant vaccination in terms of prevention of tumor relapse (Grinshtein, 2009).

1.42 Clinical data supporting the use of neoadjuvant therapy

While the many potential advantages of neoadjuvant therapy are described above, some have expressed concern that neoadjuvant therapy might actually be harmful for some patients. If neoadjuvant therapy were ineffective at regional control, then delaying surgery might convert initially resectable disease to unresectable disease. To address this concern in our proposed patient cohort, we retrospectively evaluated a series of high-risk stage III melanoma patients treated at Mayo Clinic between 2009 and 2016 with a variety of neoadjuvant therapies. Of the patients analyzed, only 6% (1/16) of patients with initially resectable or borderline-resectable disease progressed to regionally unresectable disease after neoadjuvant therapy, whereas 63% (5/8) patients with initially unresectable disease responded regionally such that disease became resectable [unpublished data]. Further, as discussed above in Section 1.3, the safety of neoadjuvant systemic therapy for multiple other epithelial cancers has been well-established. It seems logical then that with the advent of effective therapies for melanoma in the metastatic setting moving systemic therapy in part to the neoadjuvant setting for patients who are identified at the time of diagnosis as suitable candidates for systemic therapy would be as safe as and potentially more efficacious than postoperative adjuvant therapy.

In addition, there are now five open single institution phase I or II clinical trials testing neoadjuvant therapy for melanoma patients (Clinicaltrials.gov 2017). Three trials are evaluating immunotherapy. NCT01608594, a phase I trial of Stage III ≥N1b patients testing ipilimumab 10 mg plus high dose interferon$_{\alpha 2b}$ versus ipilimumab 3 mg plus high dose interferon$_{\alpha 2b}$ followed by low dose interferon$_{\alpha 2b}$ for 46 weeks postoperatively with the primary aim of safety and secondary

aims of progression-free and overall survival. A second 30 patient phase II study includes stage III and oligometastatic stage IV patients in which patients are randomized to nivolumab 3mg versus nivolumab 1mg plus ipilimumab 3 mg for two months preoperatively followed by 6 months of postoperative therapy. In this study the primary aim is pathologic complete response. A third study in the Netherlands, NCT02437279, OpACIN, is a 2 arm phase Ib trial evaluating neoadjuvant versus adjuvant nivolumab plus ipilimumab for stage IIIB melanoma patients who present with palpable disease without in-transit metastasis, of the axilla or groin. The primary aim of this 20 patient study is change in neo-antigen specific T cell response in the blood and safety while the secondary aims are recurrence-free survival and toxicity. Two additional and very similar open trials now are evaluating neoadjuvant targeted therapy for patients with BRAFm melanoma. NCT02231775, Combi-NEO, is a phase II single arm 2:1 randomization of dabrafenib plus trametinib for 8 weeks preoperatively plus 44 weeks postoperatively versus standard of care for patients with Stage IIIB, IIIC or oligometastatic Stage IV resectable BRAFm melanoma with the primary outcome of 2 year recurrence-free survival for patients with a pCR versus no pCR. Data on 21 patients treated in a similar manner has been presented in abstract form in 2016 and 2017 and the investigators reported a 77% objective response rate, a 58% pCR rate at operation and improved recurrence-free survival for the treatment arm (HR 0.017, $p<0.05$) with grade 3 adverse events in 27% of patients (Prieto 2017). A similar single institution phase II trial in Australia, NCT01972347, is testing dabrafenib plus trametinib for 12 weeks pre-operatively plus 40 weeks postoperatively for resectable BRAFm Stage IIIB and IIIC melanoma with the primary aim evaluation of the percentage of viable melanoma in the lymph nodes and secondary aims evaluating biomarkers of response, surgical outcomes and treatment-associated pyrexia (Wargo 2015).

A combined preliminary analysis of four of these trials (exclusive of the one testing interferon) was presented in the summer of 2017 (Menzies 2017). The investigators reported on 58 patients with stage III melanoma who had completed neoadjuvant therapy (18 immunotherapy, 40 targeted therapy) and proceeded to operation. They saw a pCR in 39% to immunotherapy and 55% to targeted therapy. Interestingly, after 10 months median followup, the recurrence rate was three times higher for patients treated with targeted therapy alone (12 of 40, 33%) than for immunotherapy (2 of 18, 11%). Recurrence rates were lower among patients with a pCR (0 of 7, 0%, for immunotherapy and 4 of 22, 18%, for targeted therapy) (Menzies 2017). These data, while preliminary, further support our proposed study to combine the potential benefits of targeted therapy (rapid efficacy) with immunotherapy (durability) for BRAFm and immunotherapy for BRAFwt high-risk melanoma.

1.5 Atezolizumab

Atezolizumab is a humanized IgG1 monoclonal antibody that targets programmed death ligand-1 (PD-L1) and inhibits the interaction between PD-L1 and its receptors, PD-1 and B7-1 (also known as CD80), both of which function as inhibitory receptors expressed on T cells. Therapeutic blockade of PD-L1 binding by atezolizumab has been shown to enhance the magnitude and quality of tumor-specific T-cell responses, which results in improved anti-tumor activity (Fehrenbacher 2016; Rosenberg 2016). Atezolizumab has minimal binding to Fc receptors, thus eliminating detectable Fc-effector function and associated antibody-mediated clearance of activated effector T cells.

Atezolizumab shows anti-tumor activity in both nonclinical models and cancer patients and is being investigated as a potential therapy in a wide variety of malignancies. Atezolizumab is being studied as a single agent in the advanced cancer and adjuvant therapy settings, as well as in combination with chemotherapy, targeted therapy, and other cancer immunotherapies. Atezolizumab is approved for the treatment of patients with metastatic non-small cell lung cancer whose disease progressed during or following platinum-containing chemotherapy based on the results of two international randomized, open-label clinical trials (OAK and POPLAR) (Vansteenkiste 2017). Atezolizumab now is also approved as

therapy for patients with urothelial carcinoma,non-small cell lung cancer, small-cell lung cancer, triple-negative breast cancer, hepatocellular carcinoma, and metastatic melanoma.

1.6 Vemurafenib

Vemurafenib is a compound that selectively inhibits oncogenic BRAF kinase. Discovery of oncogenic *BRAF* mutations highlights the central role of this kinase in signaling pathways that control cellular proliferation. Oncogenic mutations in *BRAF* result in constitutive activation of BRAF kinase, which causes dysregulated downstream signaling via MEK and ERK, leading to increased cell proliferation and survival.

Vemurafenib is approved as a treatment for adult patients with unresectable or metastatic $BRAF^{V600}$ mutation-positive melanoma in numerous countries worldwide, including the European Union, Switzerland, Canada, Australia, New Zealand, and Israel, and as a treatment for $BRAF^{V600E}$ mutation-positive melanoma in the United States, Brazil, and Korea.

Clinical studies of vemurafenib monotherapy support its safety, but as for other BRAF inhibitors, the superiority of combined BRAF and MEK inhibition has now been well established (Blank 2017; Ascierto 2016; Grob 2015).

1.7 Cobimetinib

Cobimetinib is a potent and highly selective inhibitor of MEK1 and MEK2, central components of the MAPK pathway. Activated MEK triggers downstream signaling through ERK to promote growth. Cancer cells transformed by $BRAF^{V600}$ are exceptionally sensitive to MEK inhibition in vitro. Allosteric MEK inhibitors can result in G1 phase growth arrest in melanoma cells (Solit 2006; Haass 2008). In vitro, MEK inhibitors reduce cell proliferation, soft agar colony formation, and matrigel invasion of $BRAF^{V600}$ mutation-positive melanoma cells, and are also effective against $BRAF^{V600}$ mutation-positive melanoma xenografts, which is suggestive of a potentially important role for MEK inhibitors in melanoma and other tumors that harbor the $BRAF^{V600}$ mutation (Solit 2006).

Cobimetinib is approved for the treatment of patients with unresectable or metastatic melanoma with a *BRAF* V600E or V600K mutation, in combination with vemurafenib. Clinical trial data in this setting supports the efficacy and safety of this combinational approach (Ascierto 2016).

1.8 Combined treatment with vemurafenib, cobimetinib and atezolizumab

Preliminary data from an ongoing Phase Ib study (GP28384) in patients with previously untreated BRAFV600 mutation-positive unresectable or metastatic melanoma indicate that treatment with atezolizumab and vemurafenib with or without cobimetinib is associated with increased response rates, as well as faster and more durable responses, compared with either cobimetinib plus vemurafenib (cobi + vem) or atezolizumab alone.

1.81 Study Design

In GP28384, treatment with atezolizumab plus vemurafenib was first evaluated, and a tolerable regimen was found. Subsequently, evaluation of the atezolizumab plus cobimetinib plus vemurafenib (atezo + cobi + vem) regimen was initiated and is ongoing with preliminary safety and efficacy information available. There are two stages in this study: a dose-escalation stage and an expansion stage. The objectives of this study are to assess the safety, tolerability, and pharmacology of atezo + cobi + vem. More mature data are currently available for the atezolizumab plus vemurafenib cohorts (median follow-up of approximately 1 year, data cutoff 1 March 2016) than for the atezo + cobi + vem cohorts (median follow-up of approximately 6 months, data cutoff 1 March 2016).

Atezolizumab is administered intravenously (IV) at a fixed dose of either 1200 mg every 3 weeks or 800 mg every 2 weeks, depending on the arm/cohort (or weight-based equivalent in the first

three cohorts). For targeted treatments, all vemurafenib dosing is by mouth (PO) twice daily (BID), and all cobimetinib dosing is PO once daily (QD) on a 21 days on/7 days off schedule. All cohorts except Cohort 1 include a run-in period consisting of targeted treatment at starting doses of 960 mg vemurafenib, with or without 60 mg cobimetinib prior to initiation of treatment with atezolizumab.

Initial cohort starting-dose levels of targeted treatments following the run-in period are 60 mg for cobimetinib plus 720 mg for vemurafenib (based on results from the ongoing Phase Ib Study GP28363 of atezo + cobi, which indicate that cobimetinib can be administered tolerably with atezolizumab at this dose and schedule).

The study enrolled four dose escalation cohorts and is currently enrolling three expansion cohorts. All cohorts except the initial cohort (Cohort 1) include a run-in period consisting of targeted treatment of vemurafenib, with or without cobimetinib, prior to initiation of treatment with atezolizumab. Cohort 1 assessed the concurrent start of atezo + vem. Cohort 2 included a 56-day run-in period with vemurafenib dosed at 960 mg BID followed by vemurafenib 720 mg BID plus atezolizumab. Cohort 3 included a 28–day run-in with vemurafenib 960 mg for 21 days, then vemurafenib 720 mg for 7 days, followed by vemurafenib 960 mg BID plus atezolizumab. Cohort 4 (and all subsequent expansion cohorts) includes a 28–day run-in with vemurafenib 960 mg for 21 days then vemurafenib 720 mg for 7 days plus cobimetinib 60 mg 21/7, followed by vemurafenib 720 mg BID plus cobimetinib 60 mg QD plus atezolizumab 800 mg every 2 weeks. Approximately 70 patients will be enrolled at approximately eight sites in the United States.

1.82 Dose-Limiting Toxicities

Approximately 24 of 30 patients evaluable for dose-limiting toxicities were enrolled in the dose-escalation cohorts with use of a modified 3 + 3 dose-escalation design to evaluate the safety, tolerability, and pharmacokinetics of treatments. There were no dose-limiting toxicities observed in any of the escalation cohorts.

1.83 Efficacy

Of the 15 patients treated with atezo + cobi + vem, 4 patients did not have a post-baseline scan at least 6 weeks from the first dose of any study drug and were considered unevaluable. Therefore, a total of 11 patients were evaluable for efficacy as of 1 March 2016. Due to limited follow-up time, unconfirmed responses are included in the preliminary efficacy results for this treatment regimen.

The 11 patients who were evaluable for efficacy all had a best response of unconfirmed PR (11/11 [100%]). Two of the 11 patients had a 100% decrease in target lesions as measured by Response Evaluation Criteria in Solid Tumors (RECIST) v1.1, one of whom had remaining disease resected and currently has no evidence of disease. One additional patient (of the 11) also had remaining disease resected and is pending evaluation for any residual evidence of disease.

Median DOR and PFS are not evaluable due to the limited follow-up time at the 1 March 2016 cutoff date. As of 1 March 2016, 9 of 11 patients continued to respond to study treatment. One patient developed a new lesion and continued study treatment with continued response in both target and non-target lesions for four cycles before discontinuing treatment. One additional patient developed new lesions and discontinued study drug due to progressive disease.

1.84 Safety

Fifteen patients have been treated with atezo + cobi + vem as of 1 March 2016 and were evaluable for safety. The median safety follow-up from the first dose of any study drug is 5.78 months (1.5–13.3 months).

Following the run-in period, the most common treatment-emergent adverse events (in ≥ 20% of patients) included photosensitivity, maculo-papular rash, ALT/AST and bilirubin elevation, fatigue, and arthralgia. A single patient discontinued as a result of an adverse event of Grade 3/4 elevation in AST simultaneous with Grade 3 ALT and total bilirubin elevation attributed to study treatment.

Five patients had a total of 10 Grade 3 adverse events and two Grade 4 adverse events that were related to atezolizumab and/or cobimetinib and/or vemurafenib following the run-in period. Grade 3 events included diverticulitis, elevated ALT, elevated AST, elevated bilirubin cellulitis, and anemia. All events except cellulitis were reported as resolved with dose modification at the time of data cutoff.

Two Grade 4 events occurred in 2 patients, both reported to be related to cobi + vem. One patient had sepsis that resolved, and the second patient had a Grade 3/4 elevation in AST simultaneous with Grade 3 ALT and total bilirubin elevation that resolved.This patient also had concurrent alkaline phosphatase (ALP) elevation as well as a liver mass and multiple gallstones (that were revealed by abdominal ultrasound). None of the patients had a Grade 5 (fatal) adverse event.

There were no serious adverse events attributed to atezolizumab. Two patients experienced serious adverse events attributed to cobimetinib and/or vemurafenib following the run-in period that included sepsis (Grade 4) and cellulitis (Grade 3). Sepsis was reported to have resolved within 6 days. The serious adverse event of cellulitis occurred in a patient with a medical history of cellulitis and was reported as not resolved as of 1 March 2016.

In summary, overall treatment with atezo + vem ± cobi, resulted in a safety profile that appears tolerable and manageable, with no Grade 5 (fatal) events and no atezolizumab-related serious adverse events. Overall, there were no unexpected adverse events, and the majority of adverse events were mild or moderate in severity, manageable with dose modification, and generally reversible.

The treatment of patients with BRAFV600 mutation–positive locally advanced or metastatic melanoma with atezo + cobi + vem is strongly supported by data presented above. Cobi + vem is approved in this indication, and demonstrates a statistically significant and clinically meaningful benefit relative to vemurafenib alone, coupled with a tolerable and manageable safety profile.

1.85 Atezolizumab Monotherapy in metastatic melanoma

In the Phase Ia setting (Study PCD4989g), atezolizumab has demonstrated activity in melanoma with a response rate comparable to that of two PD-1 inhibitors that are currently approved for the treatment of melanoma (pembrolizumab and nivolumab). Specifically, a 28% confirmed objective response rate (ORR; RECIST v1.1) in efficacy-evaluable patients with melanoma was observed, with a minimum follow-up of 15 months. In patients with cutaneous melanoma, there was a 33% confirmed ORR, and responses were continuing in 10 out of 11 responders at the time of data cutoff (January 2014).In addition, atezolizumab was generally well-tolerated by patients with metastatic melanoma.

1.86 Atezolizumab plus Cobimetinib

Study GP28363 is an ongoing Phase Ib, open-label, multicenter, global study of atezolizumab and cobimetinib in patients with metastatic or locally advanced (nonresectable) solid tumors for which no recognized standard therapy exists, including those who carry a KRAS or BRAFV600 mutation. At the 12 October 2015 data cutoff, the median safety follow up was 8.92 months. There was a confirmed ORR of 27.3% (6 of 22 patients).Safety findings have been consistent with the known adverse event profile for cobimetinib and atezolizumab and have not shown additive toxicity.

1.9 a Correlative studies

We will collect plasma, peripheral blood mononuclear cells (PBMCs), excess surgical tissue (frozen and FFPE), buccal swabs, skin swabs, and stool. These samples may be used to perform the following correlative studies: plasma soluble PD-L1 ELISA; testing for circulating tumor DNA levels; Bim levels in tumor-related T cells; RNA-Seq studies of the tumor microenvironment; and tumor, buccal, and stool microbiome studies; and sequencing of T cell receptors in peripheral blood, tumor, and lymph node tissues.

1.9b Rationale for the proposed study

Combination therapy with BRAF and MEK inhibitors and immunotherapeutic agents for patients with BRAFV600 mutation-positive melanoma is supported by a strong scientific and clinical rationale based on 1) their complementary mechanisms of action, 2) observations that RAF and MEK inhibitors may enhance anti-tumor T cell-mediated immunity (Zhu 2016), and 3) the potential to deliver both the high response rate observed with BRAF and MEK inhibitors and the prolonged duration of response (DOR) that may be elicited with immune modulation. Coupled with the clinical data described above, several lines of nonclinical and biomarker evidence support this therapeutic strategy in BRAFV600 mutation-positive melanoma.

Nonclinical studies have indicated that BRAFV600 mutation-positive melanoma induces an immunosuppressive tumor microenvironment that can be reversed by BRAF and MAPK pathway inhibitors (Sumimoto 2006).In BRAFV600 mutation-positive melanoma cell lines, BRAF inhibitors and MEK inhibitors were shown to increase antigen expression and enhance reactivity to antigen-specific T lymphocytes (Boni 2010).MEK inhibition has also been shown to increase tumor major histocompatibility complex (MHC) expression and PD-L1 expression in triple-negative breast cancer cells in vivo and in vitro (Loi 2016).

In tumor biopsies from melanoma patients, BRAF and MEK inhibitors, alone or in combination, have been shown to increase melanoma antigen expression, MHC expression, T-cell infiltration, and PD-L1 expression (Wilmott 2012; Frederick 2013; Kavakand 2015; Liu 2015; Hu-Lieskovan 2015).

Additional MAPK inhibition effects may further modulate the tumor microenvironment in ways that could enable an improved immune reaction against the tumor. These effects include increased recruitment and activation status of CD8+ and CD4+ T cells, increased tumor antigen presentation, and reduced secretion of reduced granulocyte-colony stimulating factor with reduced mobilization and activity of CD11 + GRL + myeloid-derived suppressor cells (MDSCs) (Phan 2013; Liu 2015; Ebert 2016; Loi 2016), as well as reduced expression of angiogenesis factors with altered tumor vascular support (Ciuffreda 2009; Chang 2013; Mohan2015).

Although the MAPK pathway is important for survival and function of immune cells, it has been shown that BRAF-targeted inhibition does not impair overall immune competency in cancer patients (Hong 2012).

It has been shown that the MEK inhibitor G-38963, which is mechanistically similar to cobimetinib, promotes the effector phenotype and longevity of tumor-infiltrating T cells in a CT26 colon cancer mouse model and combines synergistically with anti-PD-L1 in inhibiting growth of CT26 tumors (Ebert 2016).In addition, the MEK inhibitor trametinib has been shown to combine effectively with anti-PD-1 and anti-CTLA-4 in nonclinical models (Liu 2015).

The data described above demonstrate that inhibition of the MAPK pathway leads to an increase in immune effector cells in the tumor, thus priming the microenvironment to enable the immune system to attack the tumor. In addition, data suggest that concomitant treatment with atezolizumab in the setting of enhanced exposure of tumor-associated antigens and other immune-priming effects of these agents may improve the potency and duration of immune control potentially leading to improved survival. Since

resistance to dual agent targeted therapy is well-described and chronic MEK inhibition might deplete T cell response due to its known inhibitory effect on naïve T cell priming (Ebert 2016), we propose that a short neoadjuvant course of these agents, coupled with immune checkpoint blockade, might actually be the most optimum dosing regimen in terms of timing and duration.

Given the high response rate of patients with metastatic BRAFm melanoma to vemurafenib/cobimetinib/atezolizumab, we will treat high risk stage III BRAFm melanoma patients with vemurafenib/cobimetinib/atezolizumab neoadjuvant therapy, followed by surgery, followed by adjuvant atezolizumab.

Given the preclinical synergy between MEK inhibition and PD-1 blockade, along with the promising preliminary results of Study GP28363, we will treat high risk stage III BRAFwt melanoma patients with neoadjuvant cobimetinib/atezolizumab therapy, followed by surgery, followed by adjuvant atezolizumab.

In both cohorts of patients, we will identify biomarkers of response in blood and tumor tissue.

1.9c Addition of Arm C: Testing Neoadjuvant Atezolizumab and Tiragolumab followed by Surgery plus Adjuvant Atezolizumab

1.9c1 Rationale

Increasing evidence suggests that patients with clinically evident, high-risk, but resectable stage III melanoma who have a favorable response to neoadjuvant therapy—systemic therapy given before operation—have improved melanoma-specific survival compared to patients treated with adjuvant therapy following surgical treatment. (Blank 2018; Song 2020) Further, the neoadjuvant therapy approach, with assessment of efficacy by evaluating surgical pathology, is one avenue toward shortening of the overall duration of systemic therapy and thus decreasing cost and toxicity, while preserving benefit. Studies have suggested that an immunotherapy (versus targeted therapy) backbone leads to more durable responses, but the pathologic response rates to neoadjuvant immunotherapy are lower than those reported for neoadjuvant targeted therapy. Therefore, approaches to improve the complete pathologic response rate (pCR) or near complete pathologic response rate (ncpCR) are the focus of great current interest and warranted in order to improve the cure rate for these high-risk patients and extend their life expectancy. Reported pCR/ncpCR rates vary from 13-50% for neoadjuvant immunotherapy. Much effort is now focused on identifying the optimal combinatorial regimens to improve pCR/ncpCR rates for these patients as well as understanding the underlying mechanisms governing response, resistance, and toxicity.

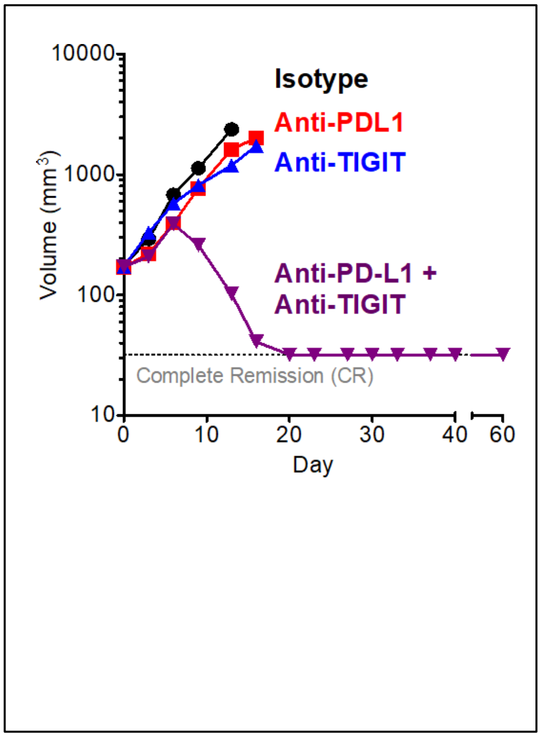

TIGIT (T cell immunoreceptor with Ig and ITIM domains) is an immunomodulatory receptor present on immune cells, including T and NK cells. TIGIT inhibits T and NK cell function by binding to its ligand on tumor cells, PVR (CD155). In the absence of TIGIT, engagement of the CD226 costimulatory receptor with CD155 on tumor cells promotes NK cell anti-tumor activity. TIGIT expression is reported to be relatively robust in melanoma-associated lymphocytes and

correlates closely with PD-1 expression. Inhibition of TIGIT with anti-TIGIT antibodies is hypothesized to complement the anti-tumor response to anti-PD-L1/anti-PD-1 antibodies, resulting in a more robust anti-tumor response than either immune checkpoint inhibitor alone.(Blessin 2019; Manieri 2017; Rotte 2018) Pre-clinical data supports this approach to reverse T cell exhaustion (Figure 1, excerpted from Johnston 2014)

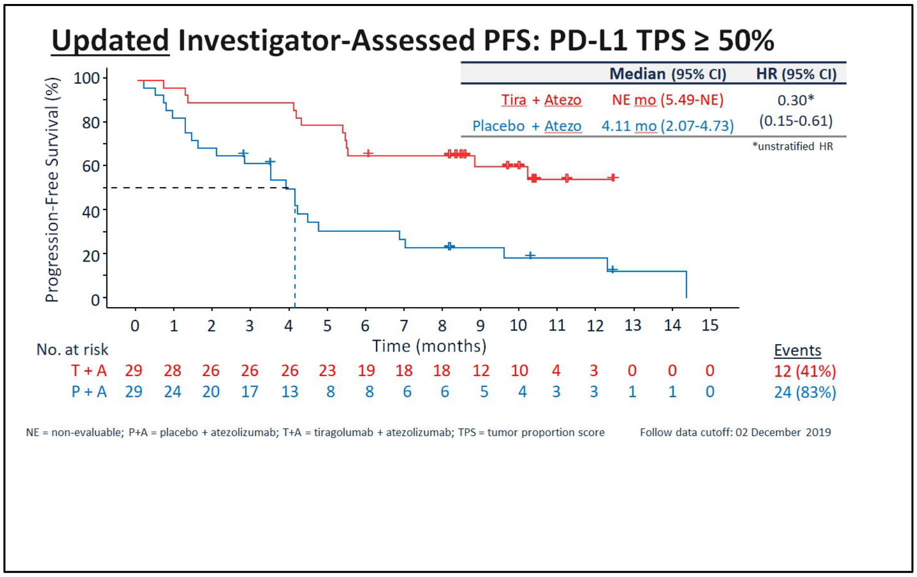

This approach was recently tested in a phase 1 clinical trial (NCT02794571) in which the anti-TIGIT antibody tiragolumab was administered in conjunction with the PD-L1 inhibitor atezolizumab and found to be active and well-tolerated in patients with a variety of solid tumors. A randomized phase II study in stage IV NSCLC testing atezolizumab + placebo versus atezolizumab + tiragolumab was just reported at ASCO in June 2020 (Rodriguez-Abreu 2020).

This study enrolled 135 patients and found the combination as compared to atezolizumab monotherapy was associated with a significantly higher overall response rate (37% versus 21%) which was significantly better for strongly PD-L1+ tumors (≥50% PD-L1+ by TPS, 66% versus 24%) and improved PFS (median not reached versus 4.1 months, HR 0.3 favoring the combination). Progression-free survival data are shown in Figure 2, excerpted from this abstract. Immune-related AEs were similar for each arm with the exception of more dermatologic and infusion-related reactions with the combination. These data suggest a sound rationale for testing this combination in the neoadjuvant setting for high-risk stage III melanoma patients. The neoadjuvant approach also facilitates correlative science to address the biologic basis of response, resistance and toxicity to further improve patient care.

Based on these promising preclinical and clinical results, we propose to test the combination of atezolizumab (anti-PD-L1) and tiragolumab (anti-TIGIT) as neoadjuvant therapy for patients with high-risk but resectable Stage III melanoma. This arm will begin accrual after completion of accrual to Arms A and B of the NeoACTIVATE (MC1776) trial. Both BRAF-mutant and BRAF-wild-type patients will be enrolled.

1.9c2 Study Design

Arms A and B of MC1776 will continue accrual without change. Following completion of accrual to Arms A and B, accrual to Arm C will open to both BRAF-mutated and BRAF-wild-type patients.

1.9c3 Correlative Studies

We plan to perform the same correlative studies for Arm C of MC1776 as for Arms A and B. These include determining the associations between soluble and tissue PD-L1 with RFS, determining the association between intracellular Bim levels on tumor-associated T cells with

RFS, evaluating associations between molecular features of melanoma (assessed by RNA-Seq) and the tumor immune microenvironment (assessed by multiplexed immunofluorescence) with RFS, evaluating changes in cell-free DNA over time and correlating these with clinical outcomes, and evaluating T cell receptor diversity and T cell neoantigen responses in peripheral blood and treated lymph nodes. We also plan to study the host microbiome at serial time points in association with the above as well as local and systemic immunity assays and treatment response.

In addition, to further characterize changes in peripheral blood mononuclear cells (PBMCs), we plan to perform CyTOF analysis of peripheral blood mononuclear cells at multiple time points and correlate changes in PBMC subsets with other biomarkers (as noted above) and with RFS.

2.0 Goals

2.1 Primary Objectives

2.11 Neoadjuvant phase primary objectives

2.111 To estimate the percentage of patients with stage III BRAFm melanoma that achieves a pathologic complete response after 12 weeks of neoadjuvant vemurafenib/cobimetinib/atezolizumab.

2.112 To estimate the percentage of patients with stage III BRAFwt melanoma that achieves a pathologic complete response after 12 weeks of neoadjuvant cobimetinib/atezolizumab.

2.113 To estimate the percentage of patients with stage III melanoma that achieves a pathologic complete response (pCR) after 12 weeks of neoadjuvant atezolizumab/tiragolumab.

2.12 Adjuvant phase primary objectives

2.121 To assess recurrence-free survival (RFS) in patients with stage III BRAFm melanoma after neoadjuvant vemurafenib/cobimetinib/atezolizumab, surgery, and adjuvant atezolizumab.

2.122 To assess RFS in patients with stage III BRAFwt melanoma after neoadjuvant cobimetinib/atezolizumab, surgery, and adjuvant atezolizumab.

2.123 To assess RFS in patients with stage III melanoma after neoadjuvant atezolizumab/tiragolumab, surgery, and adjuvant atezolizumab.

2.2 Secondary Objectives

2.21 To determine the frequency of adverse events among patients with stage III BRAFm melanoma receiving neoadjuvant vemurafenib / cobimetinib / atezolizumab followed by surgery followed by adjuvant atezolizumab.

2.22 To determine the frequency of adverse events among patients with stage III BRAFwt melanoma receiving neoadjuvant cobimetinib/atezolizumab followed by surgery followed by adjuvant atezolizumab.

2.23 To determine the frequency of adverse events among patients with stage III melanoma receiving neoadjuvant atezolizumab/tiragolumab followed by surgery followed by adjuvant atezolizumab.

2.3 Translational objectives

2.31 To determine the association between pretreatment, on treatment, post-neoadjuvant and post-adjuvant treatment sPD-L1 and RFS in patients with stage III melanoma receiving neoadjuvant vemurafenib / cobimetinib / atezolizumab or cobimetinib/atezolizumab or atezolizumab/tiragolumab, followed by surgery and adjuvant atezolizumab.

2.32 To determine the association between pretreatment, on treatment, post-neoadjuvant and post-adjuvant treatment intracellular Bim in tumor-related T cells and RFS in patients with stage III melanoma after neoadjuvant vemurafenib/cobimetinib/atezolizumab or cobimetinib/atezolizumab or atezolizumab/tiragolumab, followed by surgery and adjuvant atezolizumab.

2.33 Evaluate associations between pre and post-neoadjuvant treatment molecular features of melanomas and the tumor immune microenvironment in responders versus non-responders with MxIF (including TIGIT) and RNA-Seq in patients with stage III melanoma after neoadjuvant vemurafenib/cobimetinib/ atezolizumab or cobimetinib/atezolizumab or atezolizumab/tiragolumab, followed by surgery and adjuvant atezolizumab.

2.34 To determine the association between pretreatment tumor PD-L1, pCR, and RFS in patients with stage III melanoma receiving neoadjuvant vemurafenib/ cobimetinib/ atezolizumab or cobimetinib/atezolizumab or atezolizumab/ tiragolumab, followed by surgery and adjuvant atezolizumab.

2.35 To evaluate changes in cell-free DNA over time to determine whether these changes correlate with clinical outcomes, including pathologic complete response and cancer progression.

2.36 To compare T cell receptor diversity/clonality in pretreatment tumor, tumor and uninvolved lymph node from the time of surgery, as well as blood from before treatment, after neoadjuvant treatment, after surgery, and after adjuvant treatment.

2.37 To compare the frequency of neoantigen-specific T cells in pretreatment tumor, tumor and uninvolved lymph node from the time of surgery, as well as blood from before treatment, after neoadjuvant treatment, after surgery, and after adjuvant treatment.

2.38 To compare peripheral blood mononuclear cell subsets from before treatment, after neoadjuvant treatment, after surgery, and after adjuvant treatment via multiparametric mass cytometry (CyTOF).

3.0 Patient Eligibility

3.1 Pre-Registration – Inclusion Criteria

3.11 Age ≥18 years.

3.12 High-risk stage III melanoma, defined as (any of the following):
- recurrent nodal metastasis, or
- clinically detectable nodal metastasis, or
- metastatic involvement of more than one nodal basin

NOTE: For the purpose of pre-registration, high-risk stage III melanoma is defined based on clinical and imaging assessment (PET/CT, CT, or MRI). Histologic confirmation of nodal metastatic disease is not needed at the time of pre-registration, provided there is histologic confirmation of primary melanoma or a prior lymph node metastasis.

3.13 Willing to submit archival tissue from a lymph node biopsy or undergo a needle biopsy for BRAF testing and for research purposes.

3.14 Willing to forego anticancer treatments or investigational agents during pre- registration period.

3.15 The following laboratory values obtained ≤28 days prior to pre-registration:
- Only for patients receiving therapeutic anticoagulation: stable anticoagulant regimen and stable INR

3.2 Pre-Registration – Exclusion Criteria

3.21 Prior systemic anti-cancer therapy for melanoma (e.g., chemotherapy, hormonal therapy, targeted therapy, immunotherapy including anti-PD-1, anti-PDL1 agents, or other biologic therapies),
with the following exceptions: adjuvant treatment with interferon, IL-2, GM-CSF or vaccine therapies are allowed, if discontinued ≥28 days prior to pre-registration.

3.22 Receiving any other investigational agent which would be considered as a treatment for the primary neoplasm.

3.23 For patients with concurrent diagnosis of primary melanoma with nodal involvement, major surgical procedure other than lymph node biopsy or wide local excision of primary melanoma ≤4 weeks prior to pre-registration, or anticipation of need for a major surgical procedure for reasons other than melanoma during the course of the study.

3.24 For patients with nodal recurrence, surgical procedure or anti-cancer therapy for this recurrence (other than lymph node biopsy) or anticipation of need for a major surgical procedure for reasons other than melanoma during the course of the study.

3.25 Prior radiotherapy for melanoma

3.26 History of non-nodal melanoma metastasis or CNS lesion(s) proven or clinically suspected to be metastasis

3.27 Active malignancy (other than melanoma) or malignancy ≤3 years prior to pre-registration

NOTE: Exceptions: Asymptomatic papillary thyroid cancer (not requiring treatment), Resected basal cell carcinoma (BCC), resected cutaneous squamous cell carcinoma

(SCC), resected carcinoma in situ of the cervix, resected carcinoma in situ of the breast, in situ prostate cancer, non-muscle-invasive bladder cancer, Stage I uterine cancer, or other curatively treated malignancies from which the patient has been disease-free for at least 3 years prior to pre-registration.

3.28 Prior allogeneic stem cell or solid organ transplantation.

3.29 a History of idiopathic pulmonary fibrosis, organizing pneumonia (e.g., bronchiolitis obliterans), drug-induced pneumonitis, or idiopathic pneumonitis, or evidence of active pneumonitis on screening chest CT scan.

3.29b History of autoimmune disease requiring systemic immunosuppressive or immune-modulatory therapy ≤5 years prior to pre-registration.
NOTE: Exceptions are allowed for hypothyroidism on thyroid replacement therapy; or Type 1 diabetes on insulin regimen.

3.29c Active psoriasis requiring therapy (systemic or topical)).

3.29d Known clinically significant liver disease, including alcoholism, cirrhosis, fatty liver, and other inherited liver disease as well as active viral disease.

3.29e Arms A and B only: History of or evidence of retinal pathology on ophthalmologic examination including but not limited to:

- neurosensory retinal detachment
- central serous chorioretinopathy
- retinal vein occlusion (RVO)
- neovascular macular degeneration

3.29f Immunocompromised patients and patients known to be HIV positive and currently receiving antiretroviral therapy.
NOTE: Patients known to be HIV positive, but without clinical evidence of an immunocompromised state, are eligible for this trial.

3.29g Uncontrolled intercurrent illness including, but not limited to:

- ongoing or active infection (including but not limited to tuberculosis)
- clinically significant cardiac dysfunction including:
  - symptomatic congestive heart failure defined as New York Heart Association Class II or higher
  - unstable angina pectoris or new-onset angina ≤3 months prior to pre- registration
  - unstable cardiac arrhythmia
  - myocardial infarction ≤3 months prior to pre-registration
  - congenital long QT syndrome
- clinically significant stroke. reversible ischemic neurological defect, or transient ischemic attack ≤6 months prior to pre-registration
- any Grade 3 hemorrhage or bleeding event ≤4 weeks prior to pre-registration
- uncontrolled diabetes or symptomatic hyperglycemia
- psychiatric illness/social situations

that, in the judgement of the investigator, would:
a) limit compliance with study requirements, or
b) make the patient inappropriate for entry into this study, or
c) interfere significantly with the proper assessment of safety and toxicity of the prescribed regimens.

3.29h Known hypersensitivity to biopharmaceutical agents produced in Chinese hamster ovary cells (ex: recombinant FSH).

3.29i Known hypersensitivity to any components of the atezolizumab (all arms), tiragolumab (Arm C only), cobimetinib (Arms A and B only), or vemurafenib (Arm A only) formulations.

3.29j History of severe allergic, anaphylactic or other hypersensitivity reactions to chimeric or humanized antibodies or fusion proteins.

3.3 Registration – Inclusion Criteria

3.31 Histologic confirmation of Stage III melanoma, as defined by the American Joint Committee on Cancer, 8th revised edition.

3.32 Documentation of BRAFV600 mutation status in melanoma tumor tissue (archival or newly obtained) through use of a CLIA-approved clinical mutation test.

3.33 Surgically resectable disease, as determined by a melanoma surgical oncologist.

3.34 Eastern Cooperative Oncology Group (ECOG) Performance Status of 0 or 1 (See Appendix I).

3.35 Life expectancy ≥26 weeks.

3.36 The following laboratory values obtained ≤14 days prior to registration:

- Absolute neutrophil count (ANC) ≥1500/mm^3
- Platelet count ≥100,000/mm^3
- Hemoglobin ≥9.0 g/dL
- Direct bilirubin ≤institutional upper limit of normal (ULN)
- Aspartate transaminase (AST) and alanine transaminase (ALT) ≤2 x ULN
- Alkaline phosphatase <2.5 x ULN
- Creatinine ≤1.5 x ULN or creatinine clearance (CrCl) ≥45 mL/min on the basis of measured CrCl from a 24-hour urine collection or Cockcroft-Gault glomerular filtration rate estimation:

$$\text{CrCl}= \frac{(140 - \text{age}) \times (\text{weight in kg})}{72 \text{ x } (\text{serum creatinine in mg/dL})} \text{ (x 0.85 if female)}$$

3.37 Arms A and B only: LVEF ≥50% or institutional LLN ≤6 months prior to registration.

3.38 Arms A and B only: Average QTc≤450 ms on triplicate 12 lead ECG ≤28 days prior to registration.
NOTE: QTc intervals will be corrected using Fridericia's formula (Fridericia 1920)

3.39 a Negative pregnancy test done ≤7 days prior to registration, for persons of childbearing potential only.

3.39b For persons of childbearing potential: agreement to remain abstinent (refrain from heterosexual intercourse) or use a contraceptive method with a failure rate of <1% per year during the treatment period and for 6 months after the last dose of study treatment.

3.39c For persons able to father a child: agreement to remain abstinent (refrain from heterosexual intercourse with a person of childbearing potential) or use

contraceptive measures, and agreement to refrain from donating sperm during the treatment period and for 6 months after the last dose of study treatment.

3.39d Provide written informed consent.

3.39e Willing to return to enrolling institution for follow-up (during the Active Monitoring Phase of the study).

3.39f Willing to provide tissue, blood, and stool samples for correlative research purposes (see Sections 6.22, 14.1 and 17.1).

3.4 Registration – Exclusion Criteria

3.41 Received anticancer treatments or investigational agents during pre-registration period.

3.42 Clinically suspected non-nodal metastatic melanoma

3.43 Arm A only: For BRAF-mutant patients only: Anticipated use of any concomitant medication ≤7 days prior to registration that is known to cause QT prolongation (which may lead to torsade de pointes).

3.44 Arms A and B only: History of malabsorption or other clinically significant metabolic dysfunction that may interfere with absorption of oral study treatment or inability or unwillingness to swallow oral medication.

3.45 Signs or symptoms of infection or has received antibiotics ≤14 days prior to registration.

NOTE Patients receiving prophylactic antibiotics (e.g., to prevent a urinary tract infection or chronic obstructive pulmonary disease exacerbation) are eligible for the study.

3.46 Any of the following because this study involves investigational agents whose genotoxic, mutagenic and teratogenic effects on the developing fetus and newborn are unknown:

- Pregnant persons
- Nursing persons
- Persons of childbearing potential who are unwilling to employ adequate contraception

3.47 Treatment with a live, attenuated vaccine ≤4 weeks prior to registration, or anticipation of need for such a vaccine during the course of the study

3.48 Treatment with systemic immunosuppressive medication (including, but not limited to, prednisone, cyclophosphamide, azathioprine, methotrexate, thalidomide, and anti-tumor necrosis factor (TNF) -α agents) ≤2 weeks prior to registration, or anticipation of need for systemic immunosuppressive medication during the course of the study

NOTE: Patients who have received acute, low-dose systemic steroids (≤10 mg/day oral prednisone or equivalent) prior to registration or a one-time pulse dose of systemic immunosuppressant medication (e.g., 48 hours of corticosteroids for a contrast allergy) are eligible for the study.

NOTE: The use of inhaled corticosteroids for chronic obstructive pulmonary disease or asthma, mineralocorticoids (e.g., fludrocortisone), or low-dose corticosteroids for patients with orthostatic hypotension or adrenocortical insufficiency is allowed.

3.49a Requirement for concomitant therapy or food that is prohibited during the study, as described in Appendix III.

3.49b Arms A and B only: Inability to abstain from alcohol during neoadjuvant phase.

3.49c Arm C only: Known Epstein-Barr virus (EBV) infection.
Patients with symptoms such as splenomegaly, fever, sore throat, non-malignant cervical lymphadenopathy, and/or tonsillar exudate, should undergo an EBV polymerase chain reaction (PCR) test to screen for *acute* infection or suspected chronic active infection. Patients with a positive EBV PCR test are excluded.

4.0 Study Schedules

4.1 Test Schedule for Arms A and B

			Active Monitoring Phase								
				Neoadjuvant treatment			Surgical Period		Adjuvant Treatment		
**Tests and procedures**	Pre-registration period	≤14 days prior to registration	After registration prior to start of treatment on C1D1	D15 of each Neoadj Treatment Cycle	D8, D22 of each Neoadj Treatment Cycle	At completion of each Neoadj Treatment Cycle	After completion of ALL Neoadj Tx (prior to surgery)	At time of Surgery	≤7 days prior to start of Adjuvant Treatment	At completion of each Adjuvant Treatment Cycle: D21	Observation[1]
Window				±3 days	±3 days	±3 days	±3 days			±3 days	±14 days
History and exam, Wt, PS[2]	X			X		X			X	X	X
Height	X										
Adverse event assessment		X		X		X			X	X	X
Pregnancy test[3]		X									
Hematology group: CBC with differential		X		X		X			X	X	X
Chemistry group: AST, ALT, alkaline phosphatase, total and direct bilirubin, creatinine, random glucose		X		X	X[R4]	X			X	X	X
Neoadjuvant treatment period only: albumin, BUN, calcium, potassium, phosphorus, sodium, GGT		X		X		X					
INR[5]	X						X				
AM Cortisol							X				
TSH (reflex per institutional std)		X				X			X	X[6]	X

[1] Observation every 3 months for max of 3 years post-surgery or until disease recurrence (only for patients who undergo surgery)

[2] Exam includes monitoring for non-cutaneous SCC of head and neck consisting of at least a visual inspection of oral mucosa and lymph node palpation, and dermatologic conditions

[3] For persons of childbearing potential only: Must be done ≤7 days prior to registration.

[4] LFTs only (AST, ALT, AlkPhos, tbili, dbili); for visits when patient does not need to be at treating institutions (Mayo Clinic or UMN), LFTs may be done locally and sent to treating institutions

[5] Only for patients receiving therapeutic anticoagulation: stable anticoagulant regimen and stable INR

			Active Monitoring Phase								
				Neoadjuvant treatment			Surgical Period		Adjuvant Treatment		
**Tests and procedures**	Pre-registration period	≤14 days prior to registration	After registration prior to start of treatment on C1D1	D15 of each Neoadj Treatment Cycle	D8, D22 of each Neoadj Treatment Cycle	At completion of each Neoadj Treatment Cycle	After completion of ALL Neoadj Tx (prior to surgery)	At time of Surgery	≤7 days prior to start of Adjuvant Treatment	At completion of each Adjuvant Treatment Cycle: D21	Obser-vation[1]
Window				±3 days	±3 days	±3 days	±3 days			±3 days	±14 days
Echocardiogram or MUGA scan (LVEF)		X[7]				X[8]					
ECG[9] and CPK		X		X[10]		X					
Ophthalmic exam[11]	X[R]			X[R]							
Clinical staging	X[12]										
Pathologic staging								X			
Imaging: PET/CT (preferred), or CT and/or MRI imaging of chest, abdomen, and pelvis	X[13]						X			X[14]	X
Biopsy for BRAF testing (or archival biopsy specimen)[R]	X										
Research blood specimens (see Section 14.0) [15,R]			X[16]			X	X[17]		X	X[18]	X

[6] Thyroid testing once per cycle or as clinically indicated

[7] Baseline echocardiogram or MUGA scan obtained ≤6 months prior to registration.

[8] Repeat echo at one month after starting treatment and prior to surgery. All patients restarting treatment with a dose reduction of cobimetinib because of a decrease in LVEF should have LVEF measurements taken after approximately 2 weeks and 4 weeks or until LVEF recovers to LLN or 50%.

[9] Triplicate 12-lead ECG recordings will be obtained at screening. Single 12-lead ECG recordings will be obtained prior to and 2-4 hours after the morning doses of cobimetinib and vemurafenib for all neoadjuvant treatment cycles: Day 15. QTc intervals will be corrected using Fridericia's formula (Fridericia 1920).

[10] Repeat ECG and CPK at ~14 days after initiating neoadjuvant therapy and monthly for 3 months.

[11] Required at baseline, and after completion of first cycle of neoadjuvant treatment , then as clinically indicated based on AE assessment

[12] Submit clinical note or imaging documenting recurrent nodal metastasis, clinically detectable nodal metastasis, or metastatic involvement of more than one nodal basin

[13]≤30 days prior to registration

[14] Completion of 4th and 8th cycle of adjuvant treatment only

[15] Blood specimens will be collected and submitted per Section 14.0

[16] Research blood specimens will be collected and submitted per Section 14.0. Samples may be collected prior to registration using the pre-registration number. If the patient is not registered, the samples will be destroyed.

[17] Collect at end of all neoadjuvant treatment prior to surgery (NOTE: Different from end of cycle collection – see Section 14.0)

[18] At completion of 4th and 8th cycle of adjuvant treatment only

			Active Monitoring Phase								
				Neoadjuvant treatment			Surgical Period		Adjuvant Treatment		
**Tests and procedures**	Pre-registration period	≤14 days prior to registration	After registration prior to start of treatment on C1D1	D15 of each Neoadj Treatment Cycle	D8, D22 of each Neoadj Treatment Cycle	At completion of each Neoadj Treatment Cycle	After completion of ALL Neoadj Tx (prior to surgery)	At time of Surgery	≤7 days prior to start of Adjuvant Treatment	At completion of each Adjuvant Treatment Cycle: D21	Obser-vation[1]
Window				±3 days	±3 days	±3 days	±3 days			±3 days	±14 days
Research stool specimen, buccal (cheek) and skin swabs[19]			X				X	X[20]		X[21]	X[22]
Research tissue specimens (see Section 17.0) [23R]			X[24]					X[25]			X[26]
Medication diary[27]						X	X				

Neoadjuvant treatment cycles = 28 ±3 days (maximum of 3 cycles)

Adjuvant treatment cycles = 21 ±3 days (maximum of 8 cycles)

R= Research funded

---

[19] Microbiome specimens may be collected and processed during pre-registration period. If the patient does not register onto this study, these specimens will be destroyed. Following registration, microbiome specimens are collected and are to be submitted per Section 14.0.

[20] Skin swab collected during surgery.

[21] Microbiome stool collection at end of adjuvant therapy (≤2 weeks after last dose of study drug)

[22] Microbiome stool specimen at first recurrence (optional) to be collected and submitted per Section 14.0.

[23]Tissue specimens must be collected and submitted per Section 17.0

[24] Research tissue specimens may be collected at the same time as that needed for BRAF testing. Research tissue specimens should be processed as outlined in Section 17.0. Following registration, research tissue samples should be shipped per Section 17.0. Specimens from patients who do not register onto the study should be destroyed or keep at harvesting site according to local policies.

[25] Surgical research tissue specimens should be shipped per Section 17.0.

[26]Tissue biopsy specimen at first recurrence (optional) to be collected and submitted per Section 17.0

[27]The diary must begin the day the patient starts taking the medication and must be completed per protocol and returned to the treating institution OR compliance must be documented in the medical record by any member of the care team

4.2 Test Schedule for Arm C

			Active Monitoring Phase						
	Prior to Registration			Neoadjuvant treatment	Surgical Period		Adjuvant Treatment		
**Tests and procedures**	Pre-registration period	≤14 days prior to registration	After registration prior to start of treatment on C1D1	At completion of each Neoadj Treatment Cycle	After completion of ALL Neoadj Tx (prior to surgery)	At time of Surgery	≤7 days prior to start of Adjuvant Treatment	At completion of each Adjuvant Treatment Cycle	Obser-vation[28]
Window				±3 days	±3 days			±3 days	±14 days
History and exam, Wt, PS	X			X			X	X	X
Height	X								
Adverse event assessment		X		X			X	X	X
Pregnancy test[29]		X							
Hematology group: CBC with differential		X		X			X	X	X
Chemistry group: AST, ALT, alkaline phosphatase, total & direct bilirubin, creatinine, random glucose		X		X			X	X	X
Neoadjuvant treatment period only: potassium, sodium		X		X					
INR[30]	X				X				
AM Cortisol					X				
TSH (reflex per institutional std)		X		X			X	X[31]	X
EBV testing		X[32]							
ECG[33]		X							
Clinical staging	X[34]								
Pathologic staging						X			
Imaging per clinical standards[35]	X[36]				X			X[37]	X

[28] Observation (clinical follow-up) every 3 months for max of 3 years post-surgery or until disease recurrence (only for patients who undergo surgery)

[29] For persons of childbearing potential only: Must be done ≤7 days prior to registration.

[30] Only for patients receiving therapeutic anticoagulation: stable anticoagulant regimen and stable INR

[31] Thyroid testing once per cycle or as clinically indicated

[32] SEBV panel; If result is positive, patient is not eligible.

[33] Needed at baseline, thereafter as clinically indicated

[34] Submit clinical note or imaging documenting recurrent nodal metastasis, clinically detectable nodal metastasis, or metastatic involvement of more than one nodal basin

[35] Current Mayo Clinic standard: PET/CT (preferred), or CT and/or MRI imaging of chest, abdomen, and pelvis

[36]≤30 days prior to registration

Tests and procedures	Prior to Registration		Active Monitoring Phase						Observation[28]
				Neoadjuvant treatment	Surgical Period		Adjuvant Treatment		
	Pre-registration period	≤14 days prior to registration	After registration prior to start of treatment on C1D1	At completion of each Neoadj Treatment Cycle	After completion of ALL Neoadj Tx (prior to surgery)	At time of Surgery	≤7 days prior to start of Adjuvant Treatment	At completion of each Adjuvant Treatment Cycle	
Window				±3 days	±3 days			±3 days	±14 days
Biopsy for BRAF testing (or archival biopsy specimen)[R]	X								
Research blood specimens (see Section 14.0) [38,R]			X[39]	X	X[40]		X	X[41]	X
Research stool specimen, buccal (cheek) and skin swabs[42]			X		X	X[43]		X[44]	X[45]
Research tissue specimens (see Section 17.0) [46R]			X[47]			X[48]			X[49]

Neoadjuvant treatment cycles = 21 ±3 days (maximum of 4 cycles);
Adjuvant treatment cycles = 21 ±3 days (maximum of 8 cycles);
R= Research funded

---

[37] Completion of 4th and 8th cycle of adjuvant treatment only

[38] Blood specimens will be collected and submitted per Section 14.0

[39] Research blood specimens will be collected and submitted per Section 14.0. Samples may be collected prior to registration using the pre-registration number. If the patient is not registered, the samples will be destroyed.

[40] Collect at end of all neoadjuvant treatment prior to surgery (NOTE: Different from end of cycle collection – see Section 14.0)

[41] At completion of 4th and 8th cycle of adjuvant treatment only

[42] Microbiome specimens may be collected and processed during pre-registration period. If the patient does not register onto this study, these specimens will be destroyed. Following registration, microbiome specimens are collected and are to be submitted per Section 14.0.

[43] Skin swab collected during surgery.

[44] Microbiome stool collection at end of adjuvant therapy (≤2 weeks after last dose of study drug)

[45] Microbiome stool specimen at first recurrence (optional) to be collected and submitted per Section 14.0.

[46]Tissue specimens must be collected and submitted per Section 17.0

[47] Research tissue specimens may be collected at the same time as tissue collection for BRAF testing. Research tissue specimens should be processed as outlined in Section 17.0. Following registration, research tissue samples should be shipped per Section 17.0. Specimens from patients who do not register onto the study should be destroyed or kept at harvesting site according to local policies.

[48] Surgical research tissue specimens should be shipped per Section 17.0.

[49]Tissue biopsy specimen at first recurrence (optional) to be collected and submitted per Section 17.0

4.3 Event Monitoring/Survival Follow-up

	Event Monitoring Phase[1]				
	q. 3 months until PD	At PD	After PD q. 6 months	Death	New Primary
Event Monitoring	X	X	X	X	At each occurrence

1. If a patient is still alive 5 years after registration, no further follow-up is required.

5.0 Grouping Factor

5.1 Arms A and B only: BRAF status: mutant versus wildtype

6.0 Registration/Randomization Procedures

6.1 Pre-Registration

6.11 Pre-registering a patient

Non Mayo Clinic sites only:

To register a patient, fax (507-284-0885) a completed eligibility checklist to the Mayo Clinic Research Site Management Office between 8 a.m. and 5 p.m. Central Time Monday through Friday.

Mayo Clinic sites only:

To register a patient, access the Mayo Clinic Research Registration application. The registration application is available 24 hours a day, 7 days a week. Back up and/or system support contact information is available on the website. If unable to access the website, call the MCResearch Site Management Office at (507) 284-2753 between the hours of 8 a.m. and 5:00 p.m. Central Time (Monday through Friday).

Access and training instructions for the Research Registration Application are available on the Office of Clinical Trials web page (https://www.mayo.edu/research/centers-programs/center-clinical-translational-science/offices/office-of-clinical-trials/research-registration-application)and detail the process for completing and confirming patient registration. Prior to initiation of protocol treatment, this process must be completed in its entirety and an MCCC subject ID number must be available as noted in the instructions. It is the responsibility of the individual and institution registering the patient to confirm the process has been successfully completed prior to release of the study agent. Patient registration via the Research Registration Application can be confirmed in any of the following ways:

- Contact the Research Site Management Office (507) 284-2753. If the patient was fully registered, the MCCC Research Site Management Office staff can access the information from the centralized database and confirm the registration.
- Refer to “Instructions for Remote Registration” in section “Finding/Displaying Information about A Registered Subject.”

All institutions

6.12 Correlative studies (All institutions)

A mandatory correlative research component is part of this study. The patient will be automatically registered onto this component (see Sections 3.1 and 17.0).

6.13 Verification (All institutions)

Prior to accepting the pre-registration, the registration/randomization application will verify the following:

- IRB approval at the registering institution
- Patient pre-registration eligibility
- Existence of a signed consent form
- Existence of a signed authorization for use and disclosure of protected health information.

6.14 Pre-registration tests/procedures (All institutions)

Pre-registration tests/procedures (see Section 4.0) must be completed within the guidelines specified on the test schedule.

6.2 Registration Requirements

6.21 Registering a patient

Non Mayo Clinic sites only:

To register a patient, fax (507-284-0885) a completed eligibility checklist to the Mayo Clinic Research Site Management Office between 8 a.m. and 5 p.m. Central Time Monday through Friday.

Mayo Clinic sites only:

To register a patient, access the Mayo ClinicResearchRregistration Application at www.registration.mayo.edu. The Research Registration Application is available 24 hours a day, 7 days a week. Back up and/or system support contact information is available on the website. If unable to access the website, call the Research Site Management Office at (507) 284-2753 between the hours of 8 a.m. and 5 p.m. Central Time (Monday through Friday).

The instructions for the Research Registration Application are available on the Office of Clinical Trials web page (https://www.mayo.edu/research/centers-programs/center-clinical-translational-science/offices/office-of-clinical-trials/research-registration-application) and confirming patient registration. Prior to initiation of protocol treatment, this process must be completed in its entirety and an MCCC subject ID number must be available as noted in the instructions. It is the responsibility of the individual registering the patient to confirm the process has been successfully completed prior to release of the study agent. Patient registration via the Research Registration Application can be confirmed in any of the following ways:

- Contact the Research Site Management Office (507) 284-2753. If the patient was fully registered, the Research Site Management Office staff can access the information from the centralized database and confirm the registration.
- Refer to “Instructions for Remote Registration” in section “Finding/Displaying Information about A Registered Subject.”

6.22 Correlative studies (All institutions)

6.221 A mandatory correlative research component is part of this study. The patient will be automatically registered onto this component (see Sections 3.1, 14.0 and 17.0).

6.222 An optional correlative research component is part of this study. There will be an option to select if the patient is to be registered onto this component (see Section 17.0)

- Patient has/has not given permission to give his/her tissue sample for research testing.

6.223 At the time of registration, the following will be recorded:

- Patient has/has not given permission to store and use his/her sample(s) for future research at Mayo Clinic.
- Patient has/has not given permission to store and use his/her sample(s) for future research to learn, prevent, or treat other health problems.
- Patient has/has not given permission for MCCC to give his/her sample(s) to researchers at other institutions.

<u>All institutions</u>

6.23 IRB approvals

Documentation of IRB approval must be on file in the Research Site Management Office before an investigator may register any patients.

In addition to submitting initial IRB approval documents, ongoing IRB approval documentation must be on file (no less than annually) at the Research Site Management Office (fax: 507-284-0885). If the necessary documentation is not submitted in advance of attempting patient registration, the registration will not be accepted and the patient may not be enrolled in the protocol until the situation is resolved.

When the study has been permanently closed to patient enrollment, submission of annual IRB approvals to the Research Site Management Office is no longer necessary.

6.24 Verification

At the time of registration, the Research Registration Application will verify the following:

- IRB approval
- Patient eligibility

6.25 Pretreatment tests/procedures (see Section 4.0) must be completed within the guidelines specified on the test schedule.

6.26 Consent and treatment on this protocol must commence at Mayo Clinic Rochester or a participating site under the supervision of a medical oncologist.

6.27 Treatment cannot begin prior to registration and must begin ≤14 days after registration.

6.28 All required baseline symptoms (see Section 10.6) must be documented and graded.

6.29 Blood draw/biospecimen kits must be available on site for patients being treated outside of Mayo Clinic in Rochester, MN.

6.3 Trial Rules

The clinical trial will be conducted in compliance with regulations (21 CFR 312, 50, and 56), guidelines for Good Clinical Practice (ICH Guidance E6), and in accordance with general ethical principles outlined in the Declaration of Helsinki; informed consent will be obtained from all participating patients; the protocol and any amendments will be subject to approval by the designated IRB prior to implementation, in accordance with 21 CFR 56.103(a); and subject records will be stored in a secure location and subject confidentiality will be maintained. The investigator will be thoroughly familiar with the appropriate use of the study drug as described in the protocol and Investigator's Brochure. Essential clinical documents will be maintained to demonstrate the validity of the study and the integrity of the data collected. Master files should be established at the beginning of the study, maintained for the duration of the study and retained according to the appropriate regulations.

7.0 Protocol Treatment

7.1 Neoadjuvant Treatment Schedule

7.11 Arm A: BRAF mutant disease

Cycle 1 only

Agent	Dose	Route	Day
Vemurafenib (VEM)	960 mg	PO BID	Days 1-22
	720 mg	PO BID	Days 23-28
Cobimetinib (COBI)	60 mg	PO QD	Days 1-21

Cycle 2 and beyond for a maximum of 2 cycles

Agent	Dose	Route	**Days**
Vemurafenib (VEM)	720 mg	PO BID	Days 1-28
Cobimetinib (COBI)	60 mg	PO QD	Days 1-21
Atezolizumab (ATEZO)	840 mg	IV	Days 1, 15 (±3 days)

Cycle = 28 ±3 days; PO = by mouth; BID= twice daily; QD = once daily; IV= intravenous

7.111 General instructions for oral agents

Oral agents (vemurafenib and cobimetinib) may be taken at the same time for the morning dose.
Both agents may be taken with or without food

Do not chew or crush tablets

7.112 Missed dose instructions for oral agents

If patient misses a dose or vomits the dose, do not make up the dose. Resume treatment with next scheduled dose.

7.12 Arm B: BRAF wild-type disease (maximum of 3 cycles)

Agent	Dose	Route	Day
Cobimetinib (COBI)	60 mg	PO QD	Days 1-21 (±3 days)
Atezolizumab (ATEZO)	840 mg	IV	Days 1, 15 (±3 days)

Cycle = 28 ±3 days; PO = by mouth; QD = once daily; IV= intravenous

7.13 Arm C: BRAF wild-type or BRAF mutant disease (maximum of 4 cycles)

No premedication is indicated for the administration of Cycle 1 of atezolizumab or tiragolumab. Premedication with antihistamines, antipyretics, and/or analgesics may be administered for the second and subsequent atezolizumab infusions only, at the discretion of the investigator.

Agent	Dose	Route	Day
Atezolizumab (ATEZO)	1200 mg	IV	Day 1 (±3 days)
Tiragolumab (TIRA)	600 mg	IV	Day 1 (±3 days)

Cycle = 21 ±3 days; IV= intravenous

Atezolizumab should be infused over 30 (± 10) minutes if the previous infusion was tolerated without an infusion related reaction (IRR) or 60 (± 15) minutes if the patient experienced an IRR with the previous infusion. The infusion will be followed by a 60 minute observation period.

If the patient tolerated the previous atezolizumab infusion well, without infusion-associated adverse events, the observation period after the next and following infusions may be reduced to 30 minutes.

If the patient experienced infusion-associated adverse events in the previous infusion, the observation period should be 60 minutes.

Following the administration of atezolizumab and a 60 minute observation period, patients will receive 600 mg tiragolumab administered by IV infusion over 60 (±15) minutes on Day 1 of each 21-day cycle. The infusion will be followed by a 60 minute observation period.

Tiragolumab should be infused over 30 (±10) minutes if the previous infusion was tolerated without an infusion-related reaction, or 60 (±15) minutes if the patient experienced an infusion-related reaction with the previous infusion.

7.2 Surgery

Within 2-4 weeks of completing neoadjuvant therapy, patients with resectable disease should undergo surgery. Operation will include all affected regional nodal basins based on clinical and radiologic evaluation. Guidelines for the performance of therapeutic lymph node dissection based on site of disease are as follows:

7.21 Axillary dissection

A complete axillary lymph node dissection to include all levels of the axilla is recommended. The dissection should be governed by anatomic boundaries. Lymph node bearing tissue should be resected from within the following margins. The superior margin of dissection includes the axillary vein from the thoracic inlet (Halsted's ligament) to the latissimus dorsi tendon. The medial margin is the intercostal and serratus anterior muscles on the chest wall. The lateral margin is the edge of the latissimus dorsi muscle including those nodes which lie lateral to the thoracodorsal neurovascular bundle proximally. The inferior border is approximately the fourth intercostal space. The deep margin should be the subscapularis muscle. The long thoracic, medial pectoral and thoracodorsal nerves should be preserved.

7.22 Inguinal dissection

An inguinal lymph node dissection should be performed according to standard technique with respect to the following anatomic boundaries. All of the lymph node bearing tissue should be resected from within the following margins. The superior margin should be at least 5 cm proximal to the inguinal ligament. The inferior margin is the adductor hiatus (Hunter's canal). The medial margin should expose the edge of the adductor magnus muscle and the lateral margin is the sartorius muscle. The femoral nerve and vessels should be preserved whenever possible.

7.23 Pelvic (iliac (external and internal [hypogastric]) and obturator) dissection

A deep inguinal or pelvic lymph node dissection of the iliac (both external and internal or hypogastric) and obturator nodes should be performed when there is

evidence of disease by imaging or may be performed electively for bulky disease but is not required if anatomic and functional imaging are negative. A standard anatomic approach is recommended. When disease is identified by preoperative imaging, every effort should be made to extirpate these affected nodes in addition to all the fatty alveolar lymph node containing tissue from the femoral canal distally to the level of the common iliac bifurcation proximally. The femoral and obturator nerves should be spared.

7.24 Popliteal dissection

A therapeutic popliteal lymph node dissection consists of removal of the lymph node bearing tissue from the popliteal fossa within the following anatomic boundaries. The superior margin of dissection includes the biceps femoris and semimembranosus and semitendinosus muscles. The medial margin is made up of the semimembranosus and semitendinosus muscles. The lateral margin is the biceps femoris muscle. The inferior margins are delineated by the lateral and medial heads of the gastrocnemius muscles.

7.25 Cervical dissection

A modified radical neck dissection preserving the spinal accessory nerve and sternocleidomastoid muscle is recommended. A superficial parotidectomy should be performed for peri- or intraparotid nodal disease and considered for patients with primary tumors of the face, ear or anterior scalp but might be guided by clinical examination and imaging findings including lymphoscintigraphy if performed. The lymph node bearing tissue from within the following anatomic borders should be resected: inferior to the clavicle, superior to the mandible, mastoid, parotid gland tail, posterior to the anterior border of the trapezius muscle, and anterior to the strap muscles. The contents of the posterior triangle should be resected with preservation of the spinal accessory nerve whenever possible. A posterior or posteriolateral neck dissection with extension of the boundaries to include all of the fibrofatty nodal tissue deep to the trapezius fascia cephalad to include suboccipital and possibly retroauricular nodes and deep to include the splenius capitis, levator scapulae, and semispinalis capitis muscles may be appropriate for posterior cervical disease.

7.26 Management of disease in interval nodes

Patients with disease identified in interval or ectopic sites such as the triangular intermuscular space, the fatty and alveolar tissue around the affected node or nodes should be widely excised and consideration given to dissection of the closest proximal draining basin.

7.3 Adjuvant Treatment: Completely resected patients (All Arms)

Agent	Dose	Route	Day	ReRx
Atezolizumab (ATEZO)	1200 mg	IV	Day 1 (±3 days)	Q3w for a maximum of 8 cycles

Cycle = 21 ±3 days; IV= intravenous

8.0 Dosage Modification Based on Adverse Events

Strictly follow the modifications in this table during neoadjuvant therapy. For adjuvant therapy, these modifications should be regarded as guidelines to produce mild-to-moderate, but not debilitating, side effects. If multiple adverse events are seen, administer dose based on greatest reduction required for any single adverse event observed. Reductions or increases apply to treatment given in the preceding cycle and are based on adverse events observed since the prior dose.

→ALERT: ADR reporting may be required for some adverse events (See Section 10.0) ←

8.1 Dose Levels (Based on Adverse Events in Tables 8.2 and 8.3)

Please note there are no dose reductions for atezolizumab or tiragolumab

*Dose level 0 refers to the starting dose.

8.11 Dose levels for Vemurafenib

Dose Level	Vemurafenib (Cycle 1, D1-21)	Vemurafenib (Cycle 1, D22-28, Cycles 2-3)
0*	960 mg by mouth twice daily	720 mg by mouth twice daily
-1	720 mg by mouth twice daily	480 mg by mouth twice daily
-2	480 mg by mouth twice daily	240 mg by mouth twice daily

8.12 Dose levels for Cobimetinib

Dose level	0*	-1	-2
Cobimetinib	60 mg by mouth once daily	40 mg by mouth once daily	20 mg by mouth once daily

→ → Use the NCI Common Terminology Criteria for Adverse Events (CTCAE) current version 4.0* unless otherwise specified ← ←

* Located at http:/ctep.cancer.gov/protocolDevelopment/electronic_applications.ctc.htm

8.13 General guidance for dose modifications and treatment delays and discontinuation

There will be **no** dose modifications for **atezolizumab or tiragolumab**.

The dose of **cobimetinib** can be reduced by 20 mg (one dose level) up to two times (i.e., from 60 mg to 40 mg and then from 40 mg to 20 mg). If further dose reduction is indicated after two dose reductions, the patient must discontinue cobimetinib but may continue treatment with atezolizumab and/or vemurafenib at the investigator's discretion.

For BRAFm cohort (Arm A), the dose of **vemurafenib** can be reduced by 240 mg BID (i.e., 480 mg/day) (one dose level) up to two times (i.e., from 960 mg to 720 mg BID and then from 720 mg to 480 mg BID for Cycle 1 OR from 720 mg to 480 mg to 240 mg BID for subsequent neoadjuvant treatment cycles. If further dose reduction is indicated after two dose reductions, the patient must discontinue vemurafenib but may continue treatment with atezolizumab and/or cobimetinib at the investigator's discretion.

If **atezolizumab or tiragolumab** is withheld and corticosteroids are initiated for treatment of an adverse event, corticosteroids should be tapered over ≥1 month to ≤10 mg/day oral prednisone or equivalent before atezolizumab can be resumed. If atezolizumab is withheld for >105 days, the patient should be discontinued from atezolizumab. Study treatment may be withheld for >105 days to allow for patients to taper off corticosteroids prior to resuming treatment. Atezolizumab can be resumed after being withheld for >105 days if the investigator agrees that the patient is likely to derive clinical benefit.

If either **cobimetinib or vemurafenib** is withheld for ≥28 days because of toxicity, the patient should be discontinued from that drug, unless resumption of treatment is approved by the principal investigator after discussion with the investigator.

For BRAFwt cohort (Arm B), if **atezolizumab** is discontinued during neoadjuvant period, then cobimetinib should be discontinued, as single agent activity is not expected.

8.2 Neoadjuvant Dose Modifications Based on Adverse Events

If all study medications are discontinued or all study medications are omitted for more than 28 days due to adverse events during the neoadjuvant period, the patient should proceed to surgery (once stable).

The days on which doses are omitted are not to be made up. For example, if vemurafenib is omitted on Days 1-3 of a cycle with recovery to Grade ≤1 on Day 4 of that cycle, the patient will receive vemurafenib on only Days 4-21 of the cycle.

If all study medications are discontinued due to adverse events during the adjuvant period, the patient should proceed to observation.

Providers may hold, delay, or reduce (in line with Section 8.1) doses, even in the absence of a requirement to do so in this table (Section 8.2) per clinical judgement to maximize patient safety. For example, if the patient's AE is Grade 2, and the table does not indicate a change in action, if the provider determines that the patient's Grade 2 AE is severe enough to warrant a change in the patient's clinical care (i.e., for medically significant or intolerable Grade 2 AE despite appropriate (maximal) supportive care), then the provider may make that change, as long as the reason for the change is discussed with the PI, and documented in the medical record.

8.21 Table for Arms A and B only

**CTCAE System/Organ/Class (SOC)**	**ADVERSE EVENTS/SYMPTOMS**	**ACTIONS**		
		**Vemurafenib (BRAF-mutant only)**	**Cobimetinib**	**Atezolizumab (All patients)**
Cardiac Disorders	**Heart failure OR**<br>**Left ventricular systolic dysfunction**<br>Clinically asymptomatic<br>LVEF <40% or 40-49% and >10% decrease from baseline	Continue	Omit x 2 weeks repeat LVEF assessment<br>If LVEF>LLN and absolute LVEF <10% decrease from baseline – resume with dose reduced -1 dose level<br>If LVEF <LLN or ≥10% decrease from baseline, discontinue	Continue

CTCAE System/Organ/Class (SOC)	ADVERSE EVENTS/SYMPTOMS	ACTIONS		
		Vemurafenib (BRAF-mutant only)	Cobimetinib	Atezolizumab (All patients)
Cardiac Disorders	Clinically symptomatic	Continue	Omit x 4 weeks repeat LVEF assessment<br>If asymptomatic after 4 weeks and repeat LVEF shows >LLN and <10% decrease from baseline, resume with dose reduced -1 dose level<br>If LVEF <LLN or ≥10% decrease from baseline, discontinue.	Omit until myocarditis is ruled out by Cardiology
Endocrine disorders	**Adrenal insufficiency**<br>Grade ≥2	Continue	Continue	Omit until improves to ≤Grade 1<br>Discontinue if not improved within 12 weeks<br>Refer to endocrinologist
Endocrine disorders	**Hyperglycemia**<br>Grade 3-4	Continue	Continue	Omit and initiate treatment under endocrinologist care<br>Resume when symptoms resolve and glucose levels are stable
Endocrine disorders	**Hyperthyroidism**<br>Grade≥2 or TSH< 0.1 mU/L	Continue	Continue	Omit and initiate therapy under endocrinologist care<br>Resume when symptoms resolve to ≤Grade 1<br>Refer to Endocrinology
Endocrine disorders	**Endocrine disorders, other specify Symptomatic Hypophysitis**	Continue	Continue	Omit and refer to endocrinologist<br>Resume when symptoms resolve to ≤Grade 1
Eye disorders (refer to Ophthalmology)	**Blurred vision or**<br>**Eye pain or**<br>**Uveitis or**<br>**Eye disorders-other specify: iritis (immune-related)**<br>Grade 2	Continue	Continue	Omit<br>Resume when symptoms resolve to ≤Grade 1

CTCAE System/Organ/Class (SOC)	ADVERSE EVENTS/SYMPTOMS	ACTIONS		
		**Vemurafenib (BRAF-mutant only)**	**Cobimetinib**	**Atezolizumab (All patients)**
Eye disorders (refer to Ophthalmology)	Grade ≥3	Omit until ≤Grade 1<br>Resume with dose reduced by 1 level once ≤Grade 1	Omit until ≤Grade 1<br>Resume at same dose once ≤Grade 1	Discontinue
	**Retinopathy (serous retinopathy, chorioretinopathy) or**<br>**Retinal detachment (RPED)**<br>Grade 2-3	Omit until ≤Grade 1<br>Resume at same dose once ≤Grade 1	Omit until ≤Grade 1<br>Resume at dose reduced by 1 level once ≤Grade 1	Continue
	Grade 4	Omit until ≤Grade 1<br>Resume with dose reduced by 1 level once ≤Grade 1	Discontinue	Continue
Eye disorders (refer to Ophthalmology)	**Retinal vascular disorder (Retinal Vein Occlusion)**<br>(Any grade)	Discontinue	Discontinue	Continue
Gastrointestinal disorders	**Diarrhea/Colitis**<br>Grade 2	Omit until symptoms resolve to ≤Grade 1<br>For Grade 2 may resume previous dose level	Omit until symptoms resolve to ≤Grade 1<br>Resume at one dose level lower	Omit and resume when symptoms resolve to ≤Grade 1,
	Grade 3	Omit until symptoms resolve to ≤Grade 1<br>Resume at one dose level lower	Omit until symptoms resolve to ≤Grade 1<br>Resume at one dose level lower	Omit and resume when symptoms resolve to ≤Grade 1
	Grade 4	Discontinue	Discontinue	Discontinue
Gastrointestinal disorders	**Pancreatitis**<br>(amylase/lipase increased)<br>Grade 2	Continue	Continue	Continue at provider's discretion<br>Monitor amylase/lipase weekly<br>See Section 9.0
	Grade 3	Omit until ≤Grade 2<br>Resume with dose reduced by 1 levels once ≤Grade 2	Omit until ≤Grade 2<br>Resume with dose reduced by 1 levels once ≤Grade 2	Omit until ≤Grade 2<br>Initiate supportive care per Section 9.0<br>Resume once symptoms resolve to ≤Grade 2<br>If pancreatitis recurs, permanently discontinue atezolizumab

CTCAE System/Organ/Class (SOC)	ADVERSE EVENTS/SYMPTOMS	ACTIONS		
		**Vemurafenib (BRAF-mutant only)**	**Cobimetinib**	**Atezolizumab (All patients)**
	Grade 4	Omit until ≤Grade 2<br>Resume with dose reduced by 1 levels once ≤Grade 2	Omit until ≤Grade 2<br>Resume with dose reduced by 1 levels once ≤Grade 2	Discontinue
General disorders and administration site conditions	**Infusion-related reaction**<br>Grade ≥3	Continue	BRAFm – Continue<br>BRAFwt – Discontinue	Discontinue
Immune System Disorders	**Allergic reaction or Anaphylaxis**<br>Grade 2	Continue	Continue	Omit and resume when symptoms resolve to ≤Grade 1<br>Pre-treatment required prior to subsequent doses<br>Resume at same dose
	Grade ≥3	Discontinue	Discontinue	Discontinue
Immune System Disorders	Immune System Disorders, Other: Immune-related myasthenic syndrome/myasthenia gravis, Guillain-Barré or meningoencephalitis	Continue	Continue	Permanently discontinue atezoliuzmab
Investigations	**Aspartate aminotransferase** or **Alanine aminotransferase increased** Grade 1	Continue, but monitor LFTs weekly	Continue, but monitor LFTs weekly	Continue, but monitor LFTs weekly
	Grade 2 (AST/ALT >3.0-5.0 x ULN)	Omit treatment<br>Monitor LFTs weekly<br>Resume with dose reduced by 1 level once ≤ Grade 1	Omit treatment<br>Monitor LFTs weekly<br>Resume at previous dose level once ≤ Grade 1	Omit treatment<br>Monitor LFTs weekly<br>Resume when symptoms resolve to ≤Grade 1
	Grade 3 (AST/ALT >5.0-10.0 x ULN)	Omit until ≤Grade 1<br>Resume with dose reduced by 2 levels once ≤Grade 1	Omit until ≤Grade 1<br>Resume with dose reduced by 2 levels once ≤Grade 1	Omit and initiate systemic steroid treatment (see Section 9.0) Consult Hepatology if not improving within 1 week<br>Resume when symptoms resolve to ≤Grade 1
	Grade 3+ (AST/ALT >10.0 x ULN)	Discontinue	Discontinue	Discontinue<br>Consult Hepatology
Investigations	**Blood bilirubin increased**<br>Total bilirubin ≤2 x ULN	Continue	Continue	Continue

CTCAE System/Organ/Class (SOC)	ADVERSE EVENTS/SYMPTOMS	ACTIONS		
		**Vemurafenib (BRAF-mutant only)**	**Cobimetinib**	**Atezolizumab (All patients)**
	Total bilirubin >2 x ULN<br>For patients with Gilbert's syndrome: modify dose only if direct bilirubin is >2 x ULN	Discontinue	Discontinue	Discontinue<br>Consult Hepatology
Investigations	**CPK increased**<br>Symptomatic (Any grade) or rhabdomyolysis	Continue	Omit until ≤Grade 1<br>Resume at dose reduced one level once ≤Grade 1	Continue
Investigations	**Electrocardiogram QT corrected interval prolonged**<br>>500 ms (≥Grade 3) and change from baseline ≤60 ms<br>OR ≤500 ms and change from baseline >60 ms	Omit until ≤500 ms and change from baseline ≤60 ms<br>When recovered, resume with dose reduced by 1 level	Continue	Continue
Investigations	**Electrocardiogram QT corrected interval prolonged**<br>>500 ms and change from baseline >60 ms	Discontinue vemurafenib	Continue	Continue
Nervous system disorders	**Intracranial hemorrhage**<br>Any grade	Omit and discontinue if not resolved within 28 days	Discontinue	Continue
Renal and urinary disorders	**Acute kidney injury**<br>Grade 2	Continue	Continue	Omit until ≤Grade 1<br>Resume when symptoms resolve to ≤Grade 1
Renal and urinary disorders	Grade ≥3	Omit until ≤Grade 1<br>Resume at same dose once ≤Grade 1	BRAFm: Omit until ≤Grade 1<br>Resume at same dose once ≤Grade 1<br>BRAFwt: Discontinue	Discontinue
Respiratory, thoracic and mediastinal disorders	**Pneumonitis**<br>Grade 2	Omit until ≤Grade 1<br>Resume at same dose once ≤Grade 1	Omit until ≤Grade 1<br>Resume at same dose once ≤Grade 1	Omit until ≤Grade 1<br>Resume once symptoms resolve to ≤Grade 1
	Grade ≥3	Omit until ≤Grade 1<br>Resume with dose reduced by 1 level once ≤Grade 1	Discontinue	Discontinue

CTCAE System/Organ/Class (SOC)	ADVERSE EVENTS/SYMPTOMS	ACTIONS		
		**Vemurafenib (BRAF-mutant only)**	**Cobimetinib**	**Atezolizumab (All patients)**
Skin and subcutaneous tissue disorders	**Photosensitivity**<br>Grade 2 lasting >7 days	Omit until ≤Grade 1<br>Resume with dose reduced by 1 level once ≤Grade 1<br>If event does not resolve within 28 days, discontinue	Omit until ≤Grade 1<br>Resume at current dose once ≤Grade 1 If event does not resolve within 28 days, discontinue	Continue
	Grade 3-4	Omit until ≤Grade1<br>Resume with dose reduced by one dose level<br>If event does not resolve within 28 days, discontinue	Omit until ≤Grade 1<br>Resume at current dose level<br>If event does not resolve within 28 days, discontinue	Continue
Skin and subcutaneous tissue disorders	**Rash acneiform**<br>Grade 2	Continue	Continue	Continue<br>Initiate supportive care per Section 9.0<br>Refer to Dermatology if does not improve to ≤Grade 1 within 2 weeks
	Grade 3	Continue	Omit until ≤Grade 1<br>Resume with dose reduced by 1 level once ≤Grade 1	Omit and<br>initiate supportive care per Section 9.0<br>Refer to Dermatology<br>Resume once ≤Grade 1
	Grade 4	Discontinue	Discontinue	Omit and initiate supportive care per Section 9.0<br>Refer to Dermatology<br>Resume once ≤Grade 1
Skin and subcutaneous tissue disorders	**Rash maculo-papular**<br>Grade 2	Continue (See Section 9.0)	Continue	Continue<br>Initiate supportive care per Section 9.0<br>Refer to Dermatology if does not improve to ≤Grade 1 within 2 weeks

CTCAE System/Organ/Class (SOC)	ADVERSE EVENTS/SYMPTOMS	ACTIONS		
		**Vemurafenib (BRAF-mutant only)**	**Cobimetinib**	**Atezolizumab (All patients)**
	Grade 3	Omit until ≤Grade 1<br>Resume with dose reduced by 1 level once ≤Grade 1	Continue	Omit and iinitiate supportive care per Section 9.0<br>Refer to Dermatology<br>Resume when symptoms resolve to ≤Grade 1
	Grade 4	Discontinue	Discontinue	Discontinue
Other Adverse Events	Grade 3	Omit until ≤Grade 1<br>Resume with dose reduced by 1 level once ≤Grade 1	Omit until ≤Grade 1<br>Resume with dose reduced by 1 level once ≤Grade 1	Omit until ≤Grade 1<br>Resume once symptoms resolve to ≤Grade 1
	Grade 4	Discontinue	Discontinue	Discontinue

8.22 Table for Arm C only: Atezolizumab and Tiragolumab

If either study drug is delayed for a related toxicity, it is recommended that the other study drug is also delayed since the safety profiles for atezolizumab and tiragolumab are similar.

In case of delays in dosing of one study drug for drug-related toxicity while the other study drug is given as planned, it is recommended that the study drug being delayed will be administered at the next scheduled infusion (i.e., at the next scheduled 21-day cycle).

CTCAE System/Organ/Class (SOC)	ADVERSE EVENTS/SYMPTOMS	ACTIONS	
		**Tiragolumab**	**Atezolizumab**
Cardiac Disorders	Any which are clinically symptomatic	Omit until myocarditis is ruled out by Cardiology	Omit until myocarditis is ruled out by Cardiology
Endocrine disorders	**Adrenal insufficiency**<br>Grade ≥2	Omit until improves to ≤Grade 1<br>Discontinue if not improved within 12 weeks<br>Refer to endocrinologist	Omit until improves to ≤Grade 1<br>Discontinue if not improved within 12 weeks<br>Refer to endocrinologist
Endocrine disorders	**Hyperglycemia**<br>Grade 3-4	Omit and initiate treatment under endocrinologist care Resume when symptoms resolve and glucose levels are stable	Omit and initiate treatment under endocrinologist care Resume when symptoms resolve and glucose levels are stable
Endocrine disorders	**Hyperthyroidism**<br>Grade≥2 or TSH< 0.1 mU/L	Omit and initiate therapy under endocrinologist care Resume when symptoms resolve to ≤Grade 1<br>Refer to Endocrinology	Omit and initiate therapy under endocrinologist care Resume when symptoms resolve to ≤Grade 1<br>Refer to Endocrinology

CTCAE System/Organ/Class (SOC)	ADVERSE EVENTS/SYMPTOMS	ACTIONS	
		Tiragolumab	Atezolizumab
Endocrine disorders	**Endocrine disorders, other specify Symptomatic Hypophysitis**	Omit and refer to endocrinologist<br>Resume when symptoms resolve to ≤Grade 1	Omit and refer to endocrinologist<br>Resume when symptoms resolve to ≤Grade 1
Eye disorders (refer to Ophthalmology)	**Blurred vision or**<br>**Eye pain or**<br>**Uveitis or**<br>**Eye disorders-other specify: iritis (immune-related)**<br>Grade 2	Omit<br>Resume when symptoms resolve to ≤Grade 1	Omit<br>Resume when symptoms resolve to ≤Grade 1
	Grade ≥3	Omit until ≤Grade 1	Discontinue
Gastrointestinal disorders	**Diarrhea/Colitis**<br>Grade 2	Omit and resume when symptoms resolve to ≤Grade 1,	Omit and resume when symptoms resolve to ≤Grade 1,
	Grade 3	Omit and resume when symptoms resolve to ≤Grade 1,	Omit and resume when symptoms resolve to ≤Grade 1
	Grade 4	Discontinue	Discontinue
Gastrointestinal disorders	**Pancreatitis**<br>(amylase/lipase increased)<br>Grade 2	Continue at provider's discretion<br>Monitor amylase/lipase weekly<br>See Section 9.0	Continue at provider's discretion<br>Monitor amylase/lipase weekly<br>See Section 9.0
	Grade 3	Omit until ≤Grade 2<br>Initiate supportive care per Section 9.0<br>Resume once symptoms resolve to ≤Grade 2<br>If pancreatitis recurs, permanently discontinue	Omit until ≤Grade 2<br>Initiate supportive care per Section 9.0<br>Resume once symptoms resolve to ≤Grade 2<br>If pancreatitis recurs, permanently discontinue atezolizumab
	Grade 4	Discontinue	Discontinue
General disorders and administration site conditions	**Infusion-related reaction**<br>Grade ≥3	Discontinue<br>See Section 9.0	Discontinue<br>See Section 9.0
Immune system disorders	**Allergic reaction or Anaphylaxis**<br>Grade 2	Omit and resume when symptoms resolve to ≤Grade 1<br>Pre-treatment required prior to subsequent doses<br>Resume at same dose	Omit and resume when symptoms resolve to ≤Grade 1<br>Pre-treatment required prior to subsequent doses<br>Resume at same dose
	Grade ≥3	Discontinue	Discontinue

CTCAE System/Organ/Class (SOC)	ADVERSE EVENTS/SYMPTOMS	ACTIONS	
		**Tiragolumab**	**Atezolizumab**
Immune system disorders	**Cytokine release syndrome**<br>Grade ≥3 **per CTCAE v 5.0**	Discontinue if CRS due to tiragolumab<br>See Section 9.7	Discontinue if CRS due to atezolizumab<br>See Section 9.7
Immune system disorders	Immune System Disorders, Other: **Hemophagocytic lymphohistiocytosis (HLH) and macrophage activation syndrome (MAS)**	Discontinue<br>See Section 9.0	Discontinue<br>See Section 9.0
Immune system disorders	Immune System Disorders, Other: **Immune-related myasthenic syndrome/myasthenia gravis, Guillain-Barré or meningoencephalitis**	Permanently discontinue tiragolumab	Permanently discontinue atezolizumab
Investigations	**Aspartate aminotransferase** or **Alanine aminotransferase increased** Grade 1	Continue, but monitor LFTs weekly	Continue, but monitor LFTs weekly
	Grade 2 (AST/ALT >3.0-5.0 x ULN)	Omit treatment<br>Monitor LFTs weekly<br>Resume when symptoms resolve to ≤Grade 1	Omit treatment<br>Monitor LFTs weekly<br>Resume when symptoms resolve to ≤Grade 1
	Grade 3 (AST/ALT >5.0-10.0 x ULN)	Omit and initiate systemic steroid treatment (see Section 9.0) Consult Hepatology if not improving within 1 week<br>Resume when symptoms resolve to ≤Grade 1	Omit and initiate systemic steroid treatment (see Section 9.0) Consult Hepatology if not improving within 1 week<br>Resume when symptoms resolve to ≤Grade 1
	Grade 3+ (AST/ALT >10.0 x ULN)	Discontinue<br>Consult Hepatology	Discontinue<br>Consult Hepatology
Investigations	**Blood bilirubin increased**<br>Total bilirubin >2 x ULN<br>For patients with Gilbert's syndrome: modify dose only if direct bilirubin is >2 x ULN	Discontinue<br>Consult Hepatology	Discontinue<br>Consult Hepatology
Investigations	**Lymphocyte count decreased**<br>Grade 4	Omit until ≤Grade 2<br>Resume once ≤Grade 2	Omit until ≤Grade 2<br>Resume once ≤Grade 2
Renal and urinary disorders	**Acute kidney injury**<br>Grade 2	Omit until ≤Grade 1<br>Resume when symptoms resolve to ≤Grade 1	Omit until ≤Grade 1<br>Resume when symptoms resolve to ≤Grade 1

CTCAE System/Organ/Class (SOC)	ADVERSE EVENTS/SYMPTOMS	ACTIONS	
		**Tiragolumab**	**Atezolizumab**
Renal and urinary disorders	Grade ≥3	Discontinue	Discontinue
Respiratory, thoracic and mediastinal disorders	**Pneumonitis**<br>Grade 2	Omit until ≤Grade 1<br>Resume once symptoms resolve to ≤Grade 1	Omit until ≤Grade 1<br>Resume once symptoms resolve to ≤Grade 1
	Grade ≥3	Discontinue	Discontinue
Skin and subcutaneous tissue disorders	**Erythema multiforme**<br>Grade ≥3	Omit and initiate supportive care per Section 9.0<br>Refer to Dermatology	Omit and initiate supportive care per Section 9.0<br>Refer to Dermatology
Skin and subcutaneous tissue disorders	**Rash acneiform**<br>Grade 2	Continue<br>Initiate supportive care per Section 9.0<br>Refer to Dermatology if does not improve to ≤Grade 1 within 2 weeks	Continue<br>Initiate supportive care per Section 9.0<br>Refer to Dermatology if does not improve to ≤Grade 1 within 2 weeks
	Grade 3	Omit and initiate supportive care per Section 9.0<br>Refer to Dermatology<br>Resume once ≤Grade 1	Omit and initiate supportive care per Section 9.0<br>Refer to Dermatology<br>Resume once ≤Grade 1
	Grade 4	Omit and initiate supportive care per Section 9.0<br>Refer to Dermatology<br>Resume once ≤Grade 1	Omit and initiate supportive care per Section 9.0<br>Refer to Dermatology<br>Resume once ≤Grade 1
Skin and subcutaneous tissue disorders	**Rash maculo-papular**<br>Grade 2	Continue<br>Initiate supportive care per Section 9.0<br>Refer to Dermatology if does not improve to ≤Grade 1 within 2 weeks	Continue<br>Initiate supportive care per Section 9.0<br>Refer to Dermatology if does not improve to ≤Grade 1 within 2 weeks
	Grade 3	Omit and initiate supportive care per Section 9.0<br>Refer to Dermatology<br>Resume when symptoms resolve to ≤Grade 1	Omit and initiate supportive care per Section 9.0<br>Refer to Dermatology<br>Resume when symptoms resolve to ≤Grade 1
	Grade 4	Discontinue	Discontinue
Skin and subcutaneous tissue disorders	**Stevens-Johnson syndrome**<br>≥Grade 3	Discontinue and initiate supportive care per Section 9.0<br>Refer to Dermatology	Discontinue and initiate supportive care per Section 9.0<br>Refer to Dermatology

CTCAE System/Organ/Class (SOC)	ADVERSE EVENTS/SYMPTOMS	ACTIONS	
		**Tiragolumab**	**Atezolizumab**
Skin and subcutaneous tissue disorder	**Toxic epidermal necrolysis (TEN)**<br>Grade 4	Discontinue and initiate supportive care per Section 9.0<br>Refer to Dermatology	Discontinue and initiate supportive care per Section 9.0<br>Refer to Dermatology
Skin and subcutaneous tissue disorder	Skin and subcutaneous tissue disorder – Other<br>**Drug rash with eosinophilia and systemic symptoms (DRESS)**	Discontinue and initiate supportive care per Section 9.0<br>Refer to Dermatology	Discontinue and initiate supportive care per Section 9.0<br>Refer to Dermatology
Other Adverse Events	Grade 3	Omit until ≤Grade 1<br>Resume once symptoms resolve to ≤Grade 1	Omit until ≤Grade 1<br>Resume once symptoms resolve to ≤Grade 1
	Grade 4	Discontinue	Discontinue

8.3 Adjuvant Dose Modifications Based on Adverse Events

If atezolizumab is held for more than 12 weeks due to adverse events during the adjuvant period, the patient should discontinue atezolizumab and enter the observation phase of the trial.

CTCAE System/Organ/Class (SOC)	ADVERSE EVENTS/SYMPTOMS	Atezolizumab
Cardiac disorders	**Heart failure** OR<br>**Left ventricular systolic dysfunction**<br>Clinically symptomatic	Hold until myocarditis is ruled out by Cardiology
Endocrine disorders	**Hyperglycemia**<br>Grade 3-4	Hold and initiate insulin under endocrinologist's care<br>Resume when symptoms resolve to ≤Grade 1
Endocrine disorders	**Hyperthyroidism**<br>Grade 3-4	Hold and initiate hormonal therapy under endocrinologist's care.<br>Resume when symptoms resolve to ≤Grade 1<br>Refer to Endocrinology
Endocrine disorders	**Endocrine disorders, other specify**<br>**Symptomatic Hypophysitis**	Hold and refer to Endocrinology<br>Resume when symptoms resolve to ≤Grade 1
Eye disorders	**Blurred vision**<br>**Eye pain**<br>**Uveitis**<br>**Eye disorders-other specify: iritis**<br>(immune-related)<br>Grade 2	Hold and refer to Ophthalmology<br>Resume when symptoms resolve to ≤Grade 1
	Grade 3-4	Discontinue and refer to Ophthalmology
Eye disorders	**Retinopathy (serous retinopathy, chorioretinopathy) or**<br>**Retinal detachment (RPED)**<br>Grade 2-4	Continue and refer to Ophthalmology
Eye disorders	**Retinal vascular disorder (retinal vein occlusion)**<br>Any grade	Continue and refer to Ophthalmology
Gastrointestinal Disorders	**Diarrhea/Colitis**<br>Grade 2-3	Hold and resume when symptoms resolve to ≤Grade 1<br>Discontinue if does not resolve to ≤Grade 1 within 12 weeks
	Grade 4	Discontinue
Gastrointestinal disorders	**Pancreatitis**<br>(symptomatic and amylase/lipase increased)<br>Grade 2	Continue at provider's discretion and monitor amylase and lipase weekly<br>Resume if symptoms resolve to ≤Grade 2

CTCAE System/Organ/Class (SOC)	ADVERSE EVENTS/SYMPTOMS	Atezolizumab
Gastrointestinal disorders	Grade 3	Hold<br>Monitor amylase/lipase every other day<br>Initiate supportive care per Section 9.0<br>Refer to Gastroenterology<br>Resume when symptoms resolve to ≤Grade 2<br>If pancreatitis recurs, permanently discontinue atezolizumab
	Grade 4	Discontinue
General disorders and administration site conditions	**Infusion-related reaction**<br>Grade 3-4	Discontinue
Immune system disorders	**Allergic reaction or Anaphylaxis**<br>Grade 2	Hold and resume when symptoms resolve to ≤Grade 1<br>Pre-treatment required prior to subsequent doses (Section 9.0)
	Grade 3-4	Discontinue
Immune system disorders	**Immune system disorders, other (specify):**<br>Immune-related myasthenic syndrome/myasthenia gravis, Guillain-Barré, or meningoencephalitis	Permanently discontinue atezolizumab
Investigations	**Aspartate aminotransferase increased** or **Alanine aminotransferase increased**<br>Grade 1	Continue, and monitor LFTs weekly
	Grade 2 (AST/ALT >3.0-5.0 x ULN)	Hold treatment.<br>Monitor LFTs weekly<br>Resume when symptoms resolve to ≤Grade 1<br>Consider Hepatology consult
	Grade 3 (AST/ALT >5.0-10.0 x ULN)	Hold and initiate systemic steroid treatment (see Section 9.0).<br>Resume when symptoms resolve to ≤Grade 1<br>Refer to Hepatology
	Grade 3+ (AST/ALT >10.0 x ULN)	Discontinue<br>Refer to Hepatology
Investigations	**Blood bilirubin increased**<br>Total bilirubin >2 x ULN<br>For patients with Gilbert's syndrome: modify dose only if direct bilirubin is >2 x ULN	Discontinue<br>Refer to Hepatology
Renal and urinary disorders	**Acute kidney injury**<br>Grade 2	Hold and resume when symptoms resolve to ≤Grade 1
	Grade 3-4	Discontinue

CTCAE System/Organ/Class (SOC)	ADVERSE EVENTS/SYMPTOMS	Atezolizumab
Respiratory, thoracic and mediastinal disorders	**Pneumonitis**<br>Grade 2	Hold and resume when symptoms resolve to ≤Grade 1
	Grade 3-4	Discontinue
Skin and subcutaneous tissue disorders	**Rash acneiform**<br>Grade 3	Hold and initiate supportive care (per Section 9.0)<br>Resume when symptoms resolve to ≤Grade 1
	Grade 4	Hold until ≤Grade 1 Resume at same dose once ≤Grade 1
Skin and subcutaneous tissue disorders	**Rash maculo-papular**<br>Grade 2	Continue and initiate supportive care per Section 9.0
	Grade 3	Hold and initiate systemic steroids (per Section 9.0) and resume when symptoms resolve to ≤Grade 1
	Grade 4	Discontinue
Other Adverse Events	Grade 3	Hold and resume when symptoms resolve to ≤Grade 1
	Grade 4	Discontinue

9.0 Ancillary Treatment/Supportive Care

9.1 Full supportive care

Patients should receive full supportive care while on this study. This includes blood product support, antibiotic treatment, and treatment of other newly diagnosed or concurrent medical conditions. All blood products and concomitant medications such as antidiarrheals, analgesics, and/or antiemetics received from the first day of study treatment administration until 28 days after the final dose will be recorded in the medical records.
NOTE: Acetaminophen (Tylenol®) is prohibited starting 7 days prior to and for 30 days after neoadjuvant treatment (See Appendix III).

9.2 Hypersensitivity reaction

Patients do not require premedication prior to study treatment, as hypersensitivity reaction is not expected. In the unlikely event of a hypersensitivity reaction, treatment with antihistamines, H2 blockers, and corticosteroids is recommended. Patients should be pre-medicated with the typical regimen for subsequent cycles.

If anaphylaxis is suspected, follow institutional guidelines, including discontinuing treatment infusion and instituting appropriate supportive measures.

9.3 Concurrent enrollment in other studies

Patients may not enroll in a different clinical study, including Cancer Control studies, in which investigational procedures or agents are being used, while participating in this study.

9.4 Concurrent radiation therapy

Patients must terminate study treatment if they are to receive radiation therapy for palliative reasons as it impacts evaluation of study endpoints.

9.5 General patient monitoring and supportive care guidelines

9.51 Patients should be carefully monitored during the treatment phase and then followed appropriately. Decisions for retreatment or dose modifications/interruption should follow the guidelines in Sections 8.2-8.3.

9.52 Patients who have an ongoing study agent-related serious adverse event upon study completion or at discontinuation from the study will be contacted by the treating physician or his/her designee at least every 2 weeks until the event is resolved or determined to be irreversible.

9.6 Side effect management for atezolizumab adverse events

These are to be regarded as guidelines for managing toxicity that occurs with atezolizumab therapy and should not replace clinical judgement (i.e. patients with Grade 1 rash may require systemic steroids).

9.61 Diarrhea/Colitis

All events of diarrhea or colitis should be thoroughly evaluated for more common etiologies other than drug-induced effects.

- For events of significant duration or severity or associated with signs of systemic inflammation or acute-phase reactants, check for immune-related colitis.

9.611 Grade 1- without abdominal pain/or blood in stool and symptoms. Infectious etiologies should be ruled out. Discontinue medications that may exacerbate colitis (e.g., NSAIDS) while investigating etiology. Patients may be managed symptomatically. Instruct patients to report any increase in stools.

9.612 Grade 2- without abdominal pain/or blood in stool and symptoms <1 week, and resolve to Grade 0 or 1. Continue to monitor. Infectious etiologies should be ruled out. Discontinue medications that may exacerbate colitis (e.g., NSAIDS) while investigating etiology.

9.613 Grade 2- symptoms >1 week, should be started on steroid therapy –first choice is budesonide at 12 mg once daily (if unable to obtain budesonide and/or patent continues to have diarrhea after 72 hours of use start systemic steroids at 0.5 mg/kg/day prednisone or equivalent- can be given in two doses- especially for patients that have nocturnal diarrhea).** Infectious etiologies should be ruled out. Discontinue medications that may exacerbate colitis (e.g., NSAIDS) while investigating etiology.

9.614 Grade 3 or greater- who have other etiologies ruled out should be started on systemic steroids at 1-2 mg/kg/day prednisone or equivalent (may be given in two daily doses-especially for patients that have nocturnal diarrhea).** Assess for dehydration. Patients may require hospitalization for IV steroids (1-2 mg/kg/day methylprednisolone). Discontinue medications that may exacerbate colitis (e.g., NSAIDS) while investigating etiology.

** Once patients have improvement of symptoms to Grade 0 or 1 taper of steroids should occur over at least 1 month. If patients have been started on budesonide in addition to systemic steroids, start tapering the prednisone FIRST.

Do NOT administer loperamide in patients with ≥Grade 2 diarrhea as this may cause toxic megacolon and/or perforation.

If at any time patients experience diarrhea with the following symptoms: fever or abdominal pain patients should have a CT scan of the abdomen to rule out perforation. Emergent surgical evaluation should be performed if perforation is found. If a patient has bloody diarrhea, a Gastroenterology consult should be obtained. A Gastroenterology consult should be obtained if provider is considering infliximab for treatment of colitis.

For all patients- assess hydration status and monitor electrolytes, including magnesium.

9.62 Skin and subcutaneous tissue

disorders 9.621 Rash Acneiform

Consider doxycycline, minocycline, and/or topical steroids (Grade 2), or systemic steroids (Grade 3).

9.622 Rash Maculopapular

Grade 1 or 2- start oral non-sedating daily antihistamine (e.g., cetirizine (Zyrtec®) or loratidine (Claritin®)) and use topical

hydrocortisone cream to areas of rash with pruritis. If rash continues to progress, start systemic steroids at 0.5 mg/kg daily.

Grade 3 or greater. Initiate systemic steroid therapy at 1-2 mg/kg/day of prednisone or equivalent.

9.623 Severe Cutaneous Adverse Reactions (SCARs)

Severe Cutaneous Adverse Reactions (SCARs) are rare but potentially fatal skin toxicities frequently associated with drug use including the immune checkpoint inhibitor class. These include erythema multiforme, acute generalized exanthematous pustulosis, Stevens-Johnson syndrome (SJS), Toxic Epidermal Necrolysis (TEN), and drug rash with eosinophilia and systemic symptoms (DRESS).

For suspected SCARS, patients should be referred to a dermatologist for further diagnosis and management.

Atezolizumab should be withheld for suspected SJS or TEN

Atezolizumab should be permanently discontinued for any grade of confirmed SJS or TEN

Exercise caution in patients who have previously experienced a severe or life-threatening skin adverse reaction on prior treatment with other immune-stimulatory anti-cancer agents.

9.63 Skin lesion concerning for malignancy

Refer to Dermatology for biopsy.

9.64 Hyperthyroidism

Hyperthyroidism is usually self-limiting short-lived, and does not require intervention, unless symptomatic (e.g. tachycardia). If patient experiences tachycardia, treatment with low-dose beta blockade would be indicated. Refer patient to Endocrinology.

9.65 Hypothyroidism

Isolated hypothyroidism may be managed with replacement therapy without treatment interruption and without corticosteroids

9.66 Immune-Mediated Hypophysitis/Adrenal Insufficiency

Refer patient to Endocrinology for treatment and monitoring.

9.67 Pneumonitis

Mild to moderate events of pneumonitis have been reported with atezolizumab and cobimetinib. All pulmonary events should be thoroughly evaluated for other commonly reported etiologies such as pneumonia/infection, lymphangitic carcinomatosis, pulmonary embolism, heart failure, chronic obstructive pulmonary disease, or pulmonary hypertension. For events of pneumonitis, consider comprehensive infectious evaluation including viral etiologies.

9.671 Grade 1: No change in treatment

9.672 Grade 2: Initiate steroids at 0.5-1 mg/kg/day of prednisone or equivalent.

9.673 Grade 3 or greater: Consider hospitalization, and initiate systemic steroids

9.68 Liver Dysfunction

9.681 AST or ALT ≤3 ULN and total bilirubin <2 x ULN: No change in treatment.

9.682 AST or ALT >3 but <5 ULN or Grade 2 and total bilirubin <2 x ULN: Initiate systemic steroids at 0.5-1mg/kg/day of prednisone or equivalent. Monitor LFTs at least weekly and consider referral to Hepatology/biopsy.

9.683 AST or ALT ≥5-10 x ULN (≥Grade 3) and total bilirubin <2 x ULN: Initiate systemic steroid therapy at 1-2 mg/kg/day of prednisone or equivalent. Monitor LFTs at least weekly and consider referral to Hepatology/biopsy.

9.684 AST or ALT ≤10 x ULN and bilirubin ≥2 x ULN: Initiate intravenous corticosteroids 1-2 mg/kg/day methylprednisolone or equivalent and convert to 1-2 mg/kg/day oral prednisone or equivalent with improvement. If no improvement within 48 hours consider addition of immunosuppressive agent. Monitor LFTs at least every other day.

9.69 a Pancreatitis symptomatic (Amylase/Lipase elevations)

9.69a1 Grade 2 – elevations >3 weeks consider 10 mg/day oral prednisone or equivalent. Monitor amylase/lipase weekly.

9.69a2 Grade ≥3 - Monitor amylase/lipase every other day. Initiate 1-2 mg/kg/day oral prednisone or equivalent.

9.69b Ocular adverse events

9.69b1 Ophthalmologist should evaluate visual complaints. Complete ophthalmologic evaluation should be performed that includes visual acuity testing, intra-ocular pressure measurements, slitlamp ophthalmoscopy, indirect ophthalmoscopy, visual field testing and optical coherence tomography (OCT). Fluoroscein or indocyanine green angiogram can be considered as clinically indicated.

9.69b1 Uveitis – Initiate topical corticosteroids or topical immunosuppressive therapy.

9.7 Side effect management for combination atezolizumab/tiragolumab adverse events

These are to be regarded as guidelines for managing toxicity that occurs with atezolizumab and tiragolumab therapy and should not replace clinical judgement.

9.71 Cytokine Release Syndrome (CRS)

NCI CTCAE v5.0 and the ASTCT CRS Consensus Grading Scale should be used when reporting severity of CRS on the Adverse Event eCRF. NCI CTCAE v5.0 should be used when reporting severity of organ toxicities associated with CRS on the dedicated Cytokine Release Syndrome eCRF. Organ toxicities associated with CRS should not influence overall CRS grading.

**ASTCT CRS Consensus Grading**	
Grade	Symptoms
1	• Fever (≥38°C) with or without constitutional symptoms<br>• No hypotension<br>• No hypoxia

ASTCT CRS Consensus Grading	
Grade	Symptoms
2	• Fever combined with at least one of the following:<br>– Hypotension not requiring vasopressors<br>– Hypoxia requiring low-flow oxygen (≤6 L/min) by nasal cannula or blow-by
3	• Fever combined with at least one of the following:<br>– Hypotension requiring a vasopressor (with or without vasopressin)<br>– Hypoxia requiring high-flow oxygen (> 6 L/min) by nasal cannula, face mask, non-rebreather mask, or Venturi mask
4	• Fever combined with at least one of the following:<br>– Hypotension requiring multiple vasopressors (excluding vasopressin)<br>– Hypoxia requiring oxygen by positive pressure (e.g., CPAP, BiPAP, intubation and mechanical ventilation)
5	• Death due to CRS in which another cause is not the principal factor leading to this outcome

See 9.72 for Management of CRS.

9.72 Infusion-Related Reactions (IRRs) and Cytokine Release Syndrome

Event	Management
Grade 1 fever ≥38°C with or without constitutional symptoms	• Immediately interrupt infusion.<br>• Upon symptom resolution, wait for 30 minutes and then restart infusion at half the rate being given at the time of event onset.<br>• If the infusion is tolerated at the reduced rate for 30 minutes, the infusion rate may be increased to the original rate.<br>• If symptoms recur, discontinue infusion of this dose.<br>• Administer symptomatic treatment, including maintenance of IV fluids for hydration.<br>• In case of rapid decline or prolonged CRS (> 2 days) or in patients with significant symptoms and/or comorbidities, consider managing as per Grade 2.<br>• For subsequent infusions, consider administration of oral premedication with antihistamines, anti-pyretics, and/or analgesics, and monitor closely for CRS.
Grade 2 fever with at least one of the following:<br>• Hypotension not requiring vasopressors<br>• Hypoxia requiring low-flow oxygen (≤ 6 L/min) by nasal cannula or blow-by	• Immediately interrupt infusion.<br>• Upon symptom resolution, wait for 30 minutes and then restart infusion at half the rate being given at the time of event onset.<br>• If symptoms recur, discontinue infusion of this dose.<br>• Administer symptomatic treatment.<br>• For hypotension, administer IV fluid bolus as needed.<br>• Monitor cardiopulmonary and other organ function closely (in the ICU, if appropriate). Administer IV fluids as clinically indicated, and manage constitutional symptoms and organ toxicities as per institutional practice.<br>• Rule out other inflammatory conditions that can mimic CRS (e.g., sepsis). If no improvement within 24 hours, initiate workup and assess for signs and symptoms of HLH or MAS as described in this appendix.

Event	Management
	• Consider IV corticosteroids (e.g., methylprednisolone 2 mg/kg/day or dexamethasone 10 mg every 6 hours).<br>• Consider anti-cytokine therapy.<br>• Consider hospitalization until complete resolution of symptoms. If no improvement within 24 hours, manage as per Grade 3, that is, hospitalize patient (monitoring in the ICU is recommended), permanently discontinue atezolizumab or tiragolumab,<br>• If symptoms resolve to Grade 1 or better for 3 consecutive days, the next dose of atezolizumab or tiragolumab may be administered. For subsequent infusions, consider administration of oral premedication with antihistamines, anti-pyretics, and/or analgesics and monitor closely for CRS.<br>• If symptoms do not resolve to Grade 1 or better for 3 consecutive days
Grade 3 Fever with at least one of the following:<br>• Hypotension requiring a vasopressor (with or without vasopressin)<br>• Hypoxia requiring high-flow oxygen by nasal cannula, face mask, non-rebreather mask, or Venturi mask	• Permanently discontinue atezolizumab or tiragolumab<br>• Administer symptomatic treatment.<br>• For hypotension, administer IV fluid bolus and vasopressor as needed.<br>• Monitor cardiopulmonary and other organ function closely; monitoring in the ICU is recommended. Administer IV fluids as clinically indicated, and manage constitutional symptoms and organ toxicities as per institutional practice.<br>• Rule out other inflammatory conditions that can mimic CRS (e.g., sepsis). If no improvement within 24 hours, initiate workup and assess for signs and symptoms of HLH or MAS as described in this appendix.<br>• Administer IV corticosteroids (e.g., methylprednisolone 2 mg/kg/day or dexamethasone 10 mg every 6 hours).<br>• Consider anti-cytokine therapy.<br>• Hospitalize patient until complete resolution of symptoms. If no improvement within 24 hours, manage as per Grade 4, that is, admit patient to ICU and initiate hemodynamic monitoring, mechanical ventilation, and/or IV fluids and vasopressors as needed; for patients who are refractory to anti-cytokine therapy, experimental treatments may be considered at the discretion of the investigator and in consultation with the Sponsor/Principal Investigator
Grade 4 Fever with at least one of the following:<br>• Hypotension requiring multiple vasopressors (excluding vasopressin)<br>• Hypoxia requiring oxygen by positive pressure (e.g., CPAP, BiPAP,	• Permanently discontinue atezolizumab or tiragolumab<br>• Administer symptomatic treatment<br>• Admit patient to ICU and initiate hemodynamic monitoring, mechanical ventilation, and/or IV fluids and vasopressors as needed. Monitor other organ function closely. Manage constitutional symptoms and organ toxicities as per institutional practice.<br>• Rule out other inflammatory conditions that can mimic CRS (e.g., sepsis). If no improvement within 24 hours, initiate workup and assess for signs and symptoms of HLH or MAS as described in this appendix.<br>• Administer IV corticosteroids (e.g., methylprednisolone 2 mg/kg/day or dexamethasone 10 mg every 6 hours).<br>• Consider anti-cytokine therapy - For patients who are refractory to anti-cytokine therapy, experimental treatments may be considered at the discretion of the investigator and in consultation with the Sponsor/Principal Investigator

Event	Management
intubation and mechanical ventilation)	• Hospitalize patient until complete resolution of symptoms.

9.73 **Hemophagocytic lymphohistiocytosis and Macrophage Activation Syndrome**

Immune-mediated reactions may involve any organ system and may lead to hemophagocytic lymphohistiocytosis (HLH) and macrophage activation syndrome (MAS).

Clinical and laboratory features of severe CRS overlap with HLH, and HLH should be considered when CRS presentation is atypical or prolonged.

9.731 Patients with suspected HLH should be diagnosed according to published criteria by McClain and Eckstein (2014)[50]. A patient should be classified as having HLH if five of the following eight criteria are met:

- Fever ≥ 38.5°C
- Splenomegaly
- Peripheral blood cytopenia consisting of at least two of the following:
  - Hemoglobin < 90 g/L (9 g/dL)
  - Platelet count < 100 × 10^9/L (100,000/μL)
  - ANC < 1.0 × 10^9/L (1000/μL)
- Fasting triglycerides > 2.992 mmol/L (265 mg/dL) and/or fibrinogen < 1.5 g/L (150 mg/dL)
- Hemophagocytosis in bone marrow, spleen, lymph node, or liver
- Low or absent natural killer cell activity
- Ferritin > 500 mg/L (500 ng/mL)
- Soluble IL-2 receptor (soluble CD25) elevated ≥ 2 standard deviations above age-adjusted laboratory-specific norms

9.732 Patients with suspected MAS should be diagnosed according to published criteria for systemic juvenile idiopathic arthritis by Ravelli et al. (2016)[51]. A febrile patient should be classified as having MAS if the following criteria are met:

- Ferritin >684 mg/L (684 ng/mL)
- At least two of the following:
  - Platelet count ≤181 × 10^9/L (181,000/μL)
  - AST ≥ 48 U/L
  - Triglycerides > 1.761 mmol/L (156 mg/dL)
  - Fibrinogen ≤ 3.6 g/L (360 mg/dL)

[50] McClain KL, Eckstein O. Clinical features and diagnosis of hemophagocytic lymphohistiocytosis. Up to Date [resource on the Internet]. 2014 [updated 29 October 2018; cited: 17 May 2019]. Available from: https://www.uptodate.com/contents/clinical-features-and-diagnosis-of-hemophagocytic-lymphohistiocytosis.

[51] Ravelli A, Minoia F, Davi S, et al. 2016 classification criteria for macrophage activation syndrome complicating systemic juvenile idiopathic arthritis: a European League Against Rheumatism/American College of Rheumatology/Paediatric Rheumatology International Trials Organisation Collaborative Initiative. Ann Rheum Dis 2016;75:481[illegible]9.

9.733 If either HLH or MAS is suspected, management guidelines are as follows:

- Permanently discontinue atezolizumab and tiragolumab
- Consider patient referral to hematologist.
- Initiate supportive care, including intensive care monitoring if indicated per institutional guidelines.
- Consider initiation of IV corticosteroids, an immunosuppressive agent, and/or anti-cytokine therapy.
- If event does not respond to treatment within 24 hours, initiate treatment as appropriate according to published guidelines (La Rosée 2015[52]; Schram and Berliner 2015[53]; La Rosée et al. 2019)[54].
- If event resolves to Grade 1 or better, taper corticosteroids over ≥ 1 month.

9.8 Contraception

The treatments used in this study may have adverse effects on a fetus in utero. Furthermore, it is not known if the treatment has transient adverse effects on the composition of sperm.

For this trial, male patients will be considered to be of non-reproductive potential if they have azoospermia (whether due to having had a vasectomy or due to an underlying medical condition).

Female patients will be considered of non-reproductive potential if they are either:

(1) postmenopausal (defined as at least 12 months with no menses without an alternative medical cause; in women <45 years of age a high follicle stimulating hormone (FSH) level in the postmenopausal range may be used to confirm a post-menopausal state in women not using hormonal contraception or hormonal replacement therapy. In the absence of 12 months of amenorrhea, a single FSH measurement is insufficient.);

OR

(2) have had a hysterectomy and/or bilateral oophorectomy, bilateral salpingectomy or bilateral tubal ligation/occlusion, at least 6 weeks prior to screening;

OR

(3) have a congenital or acquired condition that prevents childbearing.

Female and male patients of reproductive potential must agree to avoid becoming pregnant or impregnating a partner, respectively, while receiving study drug and for 5 months after the last dose of study drug by complying with one of the following:

(1) practice abstinence† from heterosexual

activity; OR

(2) use (or have their partner use) acceptable contraception during heterosexual

activity. Acceptable methods of contraception are‡:

Single method (one of the following is acceptable):

[52] La Rosée P. Treatment of hemophagocytic lymphohistiocytosis in adults. Hematology Am Soc Hematol Educ Program 2015;1:190–6.

[53] Schram AM, Berliner N. How I treat hemophagocytic lymphohistiocytosis in the adult patient. Blood 2015;125:2908–14.

[54] La Rosée P, Horne A, Hines M, et al. Recommendations for the management of hemophagocytic lymphohistiocytosis in adults. Blood 2019;133:2465–77.

- intrauterine device (IUD)
- vasectomy of a female subject's male partner
- contraceptive rod implanted into the skin

Combination method (requires use of two of the following):

- diaphragm with spermicide (cannot be used in conjunction with cervical cap/spermicide)
- cervical cap with spermicide (nulliparous women only)
- contraceptive sponge (nulliparous women only)
- male condom or female condom (cannot be used together)
- hormonal contraceptive: oral contraceptive pill (estrogen/progestin pill or progestin- only pill), contraceptive skin patch, vaginal contraceptive ring, or subcutaneous contraceptive injection

†Abstinence (relative to heterosexual activity) can be used as the sole method of contraception if it is consistently employed as the subject's preferred and usual lifestyle and if considered acceptable by local regulatory agencies and ERCs/IRBs. Periodic abstinence (e.g., calendar, ovulation, sympto-thermal, post-ovulation methods, etc.) and withdrawal are not acceptable methods of contraception.

‡If a contraceptive method listed above is restricted by local regulations/guidelines, then it does not qualify as an acceptable method of contraception for patients participating at sites in this country/region.

Patients should be informed that taking the study medication may involve unknown risks to the fetus (unborn baby) if pregnancy were to occur during the study. In order to participate in the study patients of childbearing potential must adhere to the contraception requirement (described above) from the day of study medication initiation (or 14 days prior to the initiation of study medication for oral contraception) throughout the study period up to 5 months after the last dose of trial therapy. If there is any question that a patient of childbearing potential will not reliably comply with the requirements for contraception, that patient should not be entered into the study.

9.8 Use in Pregnancy and Nursing

If a patient inadvertently becomes pregnant while on treatment on this study, the patient will immediately be removed from the study. The site will contact the patient at least monthly and document the patient's status until the pregnancy has been completed or terminated. The outcome of the pregnancy will be reported to Mayo Clinic without delay and within 24 hours to Mayo Clinic if the outcome is a serious adverse experience (e.g., death, abortion, congenital anomaly, or other disabling or life-threatening complication to the mother or newborn).

The study investigator will make every effort to obtain permission to follow the outcome of the pregnancy and report the condition of the fetus or newborn. If a male subject impregnates his female partner the study personnel at the site must be informed immediately and the pregnancy reported to Mayo Clinic and followed as described above.

Since many drugs are excreted in human milk, and because of the potential for serious adverse reactions in the nursing infant, patients who are breast-feeding are not eligible for enrollment

10.0 Adverse Event (AE) Monitoring and Reporting

The site principal investigator is responsible for reporting any/all serious adverse events to the sponsor as described within the protocol, regardless of attribution to study agent or treatment procedure.

The sponsor/sponsor-investigator is responsible for notifying FDA and all participating investigators in a written safety report of any of the following:

Any suspected adverse reaction that is both serious and unexpected.

- Any findings from laboratory animal or *in vitro* testing that suggest a significant risk for human subjects, including reports of mutagenicity, teratogenicity, or carcinogenicity.
- Any findings from epidemiological studies, pooled analysis of multiple studies, or clinical studies, whether or not conducted under an IND and whether or not conducted by the sponsor, that suggest a significant risk in humans exposed to the drug
- Any clinically important increase in the rate of a serious suspected adverse reaction over the rate stated in the protocol or Investigator's Brochure (IB).

Summary of SAE Reporting for this study
(please read entire section for specific instructions):

WHO:	WHAT form:	WHERE to send:
All sites	Pregnancy Reporting<br>http://ctep.cancer.gov/protocolDevelopment/electronic_applications/docs/PregnancyReportFormUpdated.pdf	Genentech: usds_aereporting-d@gene.com<br>Mayo Sites – attach to MCCC Electronic SAE Reporting Form<br>Non Mayo sites – complete and forward to RSTP2CSAES@mayo.edu
Mayo Clinic Sites	Mayo Clinic Cancer Center SAE Reporting Form<br>http://livecycle2.mayo.edu/workspace/?startEndpoint=MC4158-56/Processes/MC4158-56-Process.MC4158-56<br>AND attach<br>MedWatch 3500A:<br>http://www.fda.gov/downloads/AboutFDA/ReportsManualsForms/Forms/UCM048334.pdf	Genentech: usds_aereporting-d@gene.com<br>Will automatically be sent to CANCERCROSAFETYIN@mayo.edu
Non-Mayo Clinic Sites	MedWatch 3500A:<br>http://www.fda.gov/downloads/AboutFDA/ReportsManualsForms/Forms/UCM048334.pdf	Genentech: usds_aereporting-d@gene.com<br>RSTP2CSAES@mayo.edu<br>and<br>MCCCCRO@mayo.edu

Definitions

Adverse Event

Any untoward medical occurrence associated with the use of a drug in humans, whether or not considered drug related.

Suspected Adverse Reaction

Any adverse event for which there is a reasonable possibility that the drug caused the adverse event.

Expedited Reporting

Events reported to sponsor within 24 hours, 5 days or 10 days of study team becoming aware of the event.

Routine Reporting

Events reported to sponsor via case report forms

Events of Interest

Events that would not typically be considered to meet the criteria for expedited reporting, but that for a specific protocol are being reported via expedited means in order to facilitate the review of safety data (may be requested by the FDA or the sponsor).

Unanticipated Adverse Device Event (UADE)

Any serious adverse effect on health or safety or any life-threatening problem or death caused by, or associated with, a device, if that effect, problem, or death was not previously identified in nature, severity, or degree of incidence in the investigational plan or application (including a supplementary plan or application), or any other unanticipated serious problem associated with a device that relates to the rights, safety, or welfare of subjects

10.1 Adverse Event Characteristics

CTCAE term (AE description) and grade: The descriptions and grading scales found in the revised NCI Common Terminology Criteria for Adverse Events (CTCAE) version

4.0 will be utilized for AE reporting. All appropriate treatment areas should have access to a copy of the CTCAE version 4.0. A copy of the CTCAE version 4.0 can be downloaded from the CTEP web site: (http://ctep.cancer.gov/protocolDevelopment/electronic_applications/ctc.htm)

a. Identify the grade and severity of the event using the CTCAE version 4.0.

b. Determine whether the event is expected or unexpected (see Section 10.2).

c. Determine if the adverse event is related to the study intervention (agent, treatment or procedure) (see Section 10.3).

d. Determine whether the event must be reported as an expedited report. If yes, determine the timeframe/mechanism (see Section 10.4).

e. Determine if other reporting is required (see Section 10.5).

f. Note: All AEs reported via expedited mechanisms must also be reported via the routine data reporting mechanisms defined by the protocol (see Sections 10.6 and 18.0).

NOTE: A severe AE is NOT the same as a serious AE, which is defined in Section 10.4.

10.2 Expected vs. Unexpected Events

Expected events - are those described within the Section 15.0 of the protocol, the study specific consent form, package insert (if applicable), and/or the investigator brochure, (if an investigator brochure is not required, otherwise described in the general investigational plan).

Unexpected adverse events or suspected adverse reactions are those not listed in Section

15.0 of the protocol, the study specific consent form, package insert (if applicable), or in the investigator brochure (or are not listed at the specificity or severity that has been observed); if an investigator brochure is not required or available, is not consistent with the risk information described in the general investigational plan.

Unexpected also refers to adverse events or suspected adverse reactions that are mentioned in the investigator brochure as occurring with a class of drugs but have not been observed with the drug under investigation.

An investigational agent/intervention might exacerbate the expected AEs associated with a commercial agent. Therefore, if an expected AE (for the commercial agent) occurs with a higher degree of severity or specificity, expedited reporting is required.

NOTE: *The consent form may contain study specific information at the discretion of the Principal Investigator; it is possible that this information may NOT be included in the protocol or the investigator brochure. Refer to protocol or IB for reporting needs.

10.3 Attribution to agent(s) or procedure

When assessing whether an adverse event (AE) is related to a medical agent(s) medical or procedure, the following attribution categories are utilized:

Definite - The AE *is clearly related* to the agent(s)/procedure.

Probable - The AE *is likely related* to the agent(s)/procedure.

Possible - The AE *may be related* to the agent(s)/procedure.

Unlikely - The AE *is doubtfully related* to the agent(s)/procedure.

Unrelated - The AE *is clearly NOT related* to the agent(s)/procedure.

10.31 EXPECTED Serious Adverse Events: Protocol Specific Exceptions to Expedited Reporting

For this protocol only, the following Adverse Events/Grades are expected to occur within this population and do not require Expedited Reporting. These events must still be reported via Routine Reporting (see Section 10.6).*

*Report any clinically important increase in the rate of a serious suspected adverse reaction (at your study site) over that which is listed in the protocol or investigator brochure as an expedited event.

*Report an expected event that is greater in severity or specificity than expected as an expedited event.

CTCAE System/Organ/Class (SOC)	Adverse event/ Symptoms	CTCAE Grade at which the event will not be reported in an expedited manner[1]
Blood and lymphatic system disorders	Anemia	≤Grade 3
	Febrile neutropenia	≤Grade 3
Cardiac Disorders	Myocardial infarction	≤Grade 3

CTCAE System/Organ/Class (SOC)	Adverse event/ Symptoms	CTCAE Grade at which the event will not be reported in an expedited manner[1]
Endocrine disorders	Hypothyroidism	≤Grade 3
	Hyperthyroidism	≤Grade 3
	Hypophysitis	≤Grade 3
Gastrointestinal disorders	Abdominal pain	≤Grade 3
	Diarrhea	≤Grade 3
	Gastric hemorrhage	≤Grade 3
	Nausea	≤Grade 3
	Vomiting	≤Grade 3
General disorders and administrations site conditions	Fatigue	≤Grade 3
	Fever	≤Grade 3
Immune system disorders	Allergic reaction	≤Grade 3
	Anaphylaxis	≤Grade 3
Infections and infestations	Hepatitis viral	≤Grade 3
Injury, poisoning, and procedural complications	Wound dehiscence	≤Grade 3
Investigations	Alkaline phosphatase increased	≤Grade 3
	Aspartate aminotransferase increased	≤Grade 3
	Blood bilirubin in creased	≤Grade 3
	Neutrophil count decreased	≤Grade 4
	Platelet count decreased	≤Grade 3
	White blood cell decreased	≤Grade 4
Musculoskeletal and connective tissue disorders	Arthralgia	≤Grade 3
	Myalgia	≤Grade 3
Respiratory, thoracic and mediastinal disorders	Pneumonitis	≤Grade 3
Nervous system disorders	Intracranial hemorrhage	≤Grade 3
	Leukoencephalopathy	≤Grade 3
	Peripheral sensory neuropathy	≤Grade 3
Renal and urinary disorders	Proteinuria	≤Grade 4
Respiratory, thoracic and mediastinal disorders	Bronchopulmonary hemorrhage	≤Grade 3
Skin and subcutaneous tissue disorders	Rash acneiform	≤Grade 4
	Rash maculo-papular	≤Grade 4
Vascular disorders	Hypertension	≤Grade 3
	Thromboembolic event	≤Grade 3

[1] These exceptions only apply if the adverse event does not result in hospitalization. If the adverse event results in hospitalization, then the standard expedited adverse events reporting requirements must be followed.

Specific protocol exceptions to expedited reporting should be reported expeditiously by investigators **ONLY** if they exceed the expected grade of the event.

The following hospitalizations are not considered to be SAEs because there is no "adverse event" (*i.e.*, there is no untoward medical occurrence) associated with the hospitalization:

- Hospitalizations for respite care
- Planned hospitalizations required by the protocol
- Hospitalization planned before informed consent (where the condition requiring the hospitalization has not changed post study drug administration)
- Hospitalization for elective procedures unrelated to the current disease and/or treatment on this trial
- Hospitalization for administration of study drug or insertion of access for administration of study drug
- Hospitalization for routine maintenance of a device (*e.g.*, battery replacement) that was in place before study entry
- Hospitalization, or other serious outcomes for signs and symptoms of progression of the cancer.

10.4 Expedited Reporting Requirements for IND/IDE Agents

10.41 Phase 1 and Early Phase 2 Studies: Expedited Reporting Requirements for Adverse Events that Occur on Studies under an IND/IDE within 30 Days of the Last Administration of the Investigational Agent/Intervention[1, 2]

FDA REPORTING REQUIREMENTS FOR SERIOUS ADVERSE EVENTS (21 CFR Part 312)

NOTE: Investigators **MUST** immediately report to the sponsor **ANY** Serious Adverse Events, whether or not they are considered related to the investigational agent(s)/intervention (21 CFR 312.64)

An adverse event is considered serious if it results in **ANY** of the following outcomes:

- Death
- A life-threatening adverse event
- An adverse event that results in inpatient hospitalization or prolongation of existing hospitalization for ≥24 hours
- A persistent or significant incapacity or substantial disruption of the ability to conduct normal life functions
- A congenital anomaly/birth defect.
- Important Medical Events (IME) that may not result in death, be life threatening, or require hospitalization may be considered serious when, based upon medical judgment, they may jeopardize the patient or subject and may require medical or surgical intervention to prevent one of the outcomes listed in this definition. (FDA, 21 CFR 312.32; ICH E2A and ICH E6).

ALL SERIOUS adverse events that meet the above criteria **MUST** be immediately reported to the sponsor within the timeframes detailed in the table below.

Hospitalization	Grade 1 and Grade 2 Timeframes	Grade 3-5 Timeframes
Resulting in Hospitalization ≥24 hrs	7 Calendar Days	24-Hour 3 Calendar Days
Not resulting in Hospitalization ≥24 hrs	Not required	

**Expedited AE reporting timelines are defined as:**<br>○ "24-Hour; 3 Calendar Days" - The AE must initially be reported within 24 hours of learning of the AE, followed by a complete expedited report within 3 calendar days of the initial 24-hour report.<br>○ **"7 Calendar Days" - A complete expedited report on the AE must be submitted within 7 calendar days of learning of the AE.**
[1]Serious adverse events that occur more than 30 days after the last administration of investigational agent/intervention and have an attribution of possible, probable, or definite require reporting as follows:<br>**Expedited 24-hour notification followed by complete report within 3 calendar days for:**<br>• All Grade 4, and Grade 5 AEs<br>**Expedited 7 calendar day reports for:**<br>• Grade 2 adverse events resulting in hospitalization or prolongation of hospitalization<br>• Grade 3 adverse events<br>[2] For studies using PET or SPECT IND agents, the AE reporting period is limited to 10 radioactive half-lives, rounded UP to the nearest whole day, after the agent/intervention was last administered. Footnote "1" above applies after this reporting period.<br>Effective Date: May 5, 2011Effective Date: May 5, 2011

NOTE: Refer to Section 10.32 for exceptions to Expedited Reporting

10.42 General reporting instructions

The Mayo IND Coordinator will assist the sponsor-investigator in the processing of expedited adverse events and forwarding of suspected unexpected serious adverse reactions (SUSARs) to the FDA and IRB.

Mayo Clinic Sites: Use Mayo Expedited Event Report form http://livecycle2.mayo.edu/workspace/?startEndpoint=MC4158-56/Processes/MC4158-56-Process.MC4158-56 for investigational agents or commercial/investigational agents on the same arm.

For commercial agents:

Submit form MedWatch 3500A to the FDA, 5600 Fishers Lane, Rockville, MD 20852-9787, by fax at 1-800-332-0178 or online at http://www.fda.gov/Safety/MedWatch/HowToReport/default.htm

And send a copy to Genentech Drug Safety:
Fax: 650-238-6067
Email: usds_aereporting-d@gene.com

Non-MCCC Institutions: Submit copies to MCCCCRO@mayo.edu and RSTP2CSAES@mayo.edu

Submit form MedWatch 3500A to the FDA, 5600 Fishers Lane, Rockville, MD 20852-9787, by fax at 1-800-332-0178 or online at http://www.fda.gov/Safety/MedWatch/HowToReport/default.htm

And send a copy to Genentech Drug Safety:
Fax: 650-238-6067
Email: usds_aereporting-d@gene.com

10.43 Reporting of re-occurring SAEs

ALL SERIOUS adverse events that meet the criteria outlined in table10.41 MUST be immediately reported to the sponsor within the timeframes detailed in the corresponding table. This reporting includes, but is not limited to SAEs that re-occur again after resolution.

10.5 Other Required Reporting

10.51 Unanticipated Problems Involving Risks to Subjects or Others (UPIRTSOS)

Unanticipated Problems Involving Risks to Subjects or Others (UPIRTSOS) in general, include any incident, experience, or outcome that meets **all** of the following criteria:

- Unexpected (in terms of nature, severity, or frequency) given (a) the research procedures that are described in the protocol-related documents, such as the IRB-approved research protocol and informed consent document; and (b) the characteristics of the subject population being studied;
- Related or possibly related to participation in the research (in this guidance document, possibly related means there is a reasonable possibility that the incident, experience, or outcome may have been caused by the procedures involved in the research); and
- Suggests that the research places subjects or others at a greater risk of harm (including physical, psychological, economic, or social harm) than was previously known or recognized.

Some unanticipated problems involve social or economic harm instead of the physical or psychological harm associated with adverse events. In other cases, unanticipated problems place subjects or others at increased *risk* of harm, but no harm occurs.

Note: If there is no language in the protocol indicating that pregnancy is not considered an adverse experience for this trial, and if the consent form does not indicate that subjects should not get pregnant/impregnate others, then any pregnancy in a subject/patient or a male patient's partner (spontaneously reported) which occurs during the study or within 120 days of completing the study should be reported as a UPIRTSO.

Mayo Clinic Cancer Center (MCCC) Institutions:

If the event meets the criteria for IRB submission as a Reportable Event/UPIRTSO, provide the Reportable Event coversheet and appropriate documentation to CANCERCROSAFETYIN@mayo.edu. The Mayo Regulatory Affairs Office will review and process the submission to the Mayo Clinic IRB.

10.52 Death

Note: A death on study requires both routine and expedited reporting regardless of causality, unless as noted below. Attribution to treatment or other cause must be provided.

Any death occurring within 30 days of the last dose, regardless of attribution to an agent/intervention under an IND/IDE requires expedited reporting within 24-hours.

Any death occurring greater than 30 days with an attribution of possible, probable, or definite to an agent/intervention under an IND/IDE requires expedited reporting within 24-hours.

Reportable categories of Death

- Death attributable to a CTCAE term.
- Death Neonatal: A disorder characterized by cessation of life during the first 28 days of life.

- Death NOS: A cessation of life that cannot be attributed to a CTCAE term associated with Grade 5.
- Sudden death NOS: A sudden (defined as instant or within one hour of the onset of symptoms) or an unobserved cessation of life that cannot be attributed to a CTCAE term associated with Grade 5.
- Death due to progressive disease should be reported as Grade 5 "Neoplasms benign, malignant and unspecified (including cysts and polyps) – Other (Progressive Disease)" under the system organ class (SOC) of the same name. Evidence that the death was a manifestation of underlying disease (e.g., radiological changes suggesting tumor growth or progression: clinical deterioration associated with a disease process) should be submitted.

10.53 Secondary Malignancy

- **A *secondary malignancy*** is a cancer caused by treatment for a previous malignancy (e.g., treatment with investigational agent/intervention, radiation or chemotherapy). A secondary malignancy is not considered a metastasis of the initial neoplasm.
- All secondary malignancies that occur following treatment with an agent under an IND/IDE will be reported. Three options are available to describe the event:
    - Leukemia secondary to oncology chemotherapy (e.g., Acute Myeloctyic Leukemia [AML])
    - Myelodysplastic syndrome (MDS)
    - Treatment-related secondary malignancy
- Any malignancy possibly related to cancer treatment (including AML/MDS) should also be reported via the routine reporting mechanisms outlined in each protocol.

10.54 Second Malignancy

A second malignancy is one unrelated to the treatment of a prior malignancy (and is NOT a metastasis from the initial malignancy). Second malignancies require ONLY routine reporting unless otherwise specified.

10.55 Pregnancy, Fetal Death, and Death Neonatal

If a female subject (or female partner of a male subject) taking investigational product becomes pregnant, the subject taking should notify the Investigator, and the pregnant female should be advised to call her healthcare provider immediately. The patient should have appropriate follow-up as deemed necessary by her physician. If the baby is born with a birth defect or anomaly, a second expedited report is required.

Prior to obtaining private information about a pregnant woman and her infant, the investigator must obtain consent from the pregnant woman and the newborn infant's parent or legal guardian before any data collection can occur. A consent form will need to be submitted to the IRB for these subjects if a pregnancy occurs. If informed consent is not obtained, no information may be collected.

In cases of fetal death, miscarriage or abortion, the mother is the patient. In cases where the child/fetus experiences a serious adverse event other than fetal death, the child/fetus is the patient.

NOTE: When submitting Mayo Expedited Adverse Event Report reports for "Pregnancy", "Pregnancy loss", or "Neonatal loss", the potential risk of exposure

of the fetus to the investigational agent(s) or chemotherapy agent(s) should be documented in the "Description of Event" section. Include any available medical documentation. Include this form: http://ctep.cancer.gov/protocolDevelopment/electronic_applications/docs/PregnancyReportFormUpdated.pdf

10.551 Pregnancy

Pregnancy should be reported in an expedited manner as **Grade 3 "Pregnancy, puerperium and perinatal conditions - Other (pregnancy)"** under the Pregnancy, puerperium and perinatal conditions SOC. Pregnancy should be followed until the outcome is known.

10.552 Fetal Death

Fetal death is defined in CTCAE as "A disorder characterized by death in utero; failure of the product of conception to show evidence of respiration, heartbeat, or definite movement of a voluntary muscle after expulsion from the uterus, without possibility of resuscitation."

Any fetal death should be reported expeditiously, as **Grade 4 "Pregnancy, puerperium and perinatal conditions - Other (pregnancy loss)"** under the Pregnancy, puerperium and perinatal conditions SOC.

10.553 Death Neonatal

Neonatal death, defined in CTCAE as "A disorder characterized by cessation of life occurring during the first 28 days of life" that is felt by the investigator to be at least possibly due to the investigational agent/intervention, should be reported expeditiously.

A neonatal death should be reported expeditiously as **Grade 4 "General disorders and administration - Other (neonatal loss)"** under the General disorders and administration SOC.

10.56 AEs of Special Interest (AESIs)

AEs of Special Interest are defined as a potential safety problem, identified as a result of safety monitoring of the Product. AESIs are a subset of Events to Monitor (EtMs) of scientific and medical concern specific to the product, for which ongoing monitoring and rapid communication by the Investigator to the Sponsor is required. Such an event might require further investigation in order to characterize and understand it. Depending on the nature of the event, rapid communication by the trial Sponsor to other parties (e.g., Regulatory Authorities) may also be warranted.

Adverse events of special interest for this study include the following:

- Cases of potential drug-induced liver injury that include an elevated ALT or AST in combination with either an elevated bilirubin or clinical jaundice, as defined by Hy's law:
    - Treatment-emergent ALT or AST >3 × ULN in combination with total bilirubin >2 × ULN
    - Treatment-emergent ALT or AST >3 × ULN in combination with clinical jaundice

- Data related to a suspected transmission of an infectious agent by the study drug (STIAMP), as defined below:

  Any organism, virus, or infectious particle (e.g., prion protein transmitting transmissible spongiform encephalopathy), pathogenic or non-pathogenic, is considered an infectious agent. A transmission of an infectious agent may be suspected from clinical symptoms or laboratory findings that indicate an infection in a patient exposed to a medicinal product. This term applies only when a contamination of the study drug is suspected

10.561 **Adverse Events of Special Interest specific to Atezolizumab and/or Tiragolumab:**

- Pneumonitis
- Colitis
- Endocrinopathies: diabetes mellitus, pancreatitis, adrenal insufficiency, hyperthyroidism, and hypophysitis
- Hepatitis, including AST or ALT > 10 × ULN
- Systemic lupus erythematosus
- Neurological disorders: Guillain-Barré syndrome, myasthenic syndrome or myasthenia gravis, and meningoencephalitis
- Events suggestive of hypersensitivity, infusion-related reactions, cytokine release syndrome, influenza-like illness, macrophage activating syndrome, and hemophagocytic lymphohistiocytosis
- Nephritis
- Ocular toxicities (e.g., uveitis, retinitis, optic neuritis)
- Myositis
- Myopathies, including rhabdomyolysis
- Grade ≥2 cardiac disorders (e.g., atrial fibrillation, myocarditis, pericarditis)
- Vasculitis
- Autoimmune hemolytic anemia
- Severe cutaneous reactions (e.g. Stevens-Johnson Syndrome, dermatitis bullous, toxic epidermal necrolysis)

10.562 **Adverse events of Special Interest Specific to Vemurafenib:**

- Acute kidney injury
- Cutaneous squamous cell carcinomas
- Liver injury
- Non cutaneous squamous cell carcinomas
- Pancreatitis
- Progression of RAS mutant malignancies
- QT prolongation

10.563 **Adverse events of Special Interest Specific to Cobimetinib:**

- Any grade serous retinopathy, including events of retinal detachment, retinal pigment epithelium detachment, neurosensory retinal detachment or central serous chorioretinopathy
- Significant liver toxicity: AST and/or ALT > 10 X upper limit of normal

- Symptomatic heart failure / Grade ≥ 2 left ventricular dysfunction
- Grade ≥ 3 CPK elevation or rhabdomyolysis
- Grade ≥ 3 diarrhea
- Grade ≥ 3 rash

10.6 Required Routine Reporting

10.61 Baseline and Adverse Events Evaluations

Pretreatment symptoms/conditions to be graded at baseline and adverse events to be graded at each evaluation.
Grading is per CTCAE v4.0 **unless** alternate grading is indicated in the table below:

CTCAE System Organ Class (SOC)	Adverse event/Symptoms	Baseline	Each evaluation
Blood and lymphatic system disorders	Anemia	X	X
Gastrointestinal disorders	Baseline # stools	X	
	Diarrhea		X
General disorders	Fatigue	X	X
	Fever		X
Immune system disorders	Allergic reaction		X
	Anaphylaxis		X
Investigations	Blood bilirubin increased	X	X
Respiratory, thoracic and mediastinal disorders	Dyspnea	X	X
Skin and subcutaneous tissue disorders	Rash, acneiform	X	X
	Rash, maculo-papular	X	X

10.62 Other routine reporting

Submit via appropriate MCCC Case Report Forms (i.e., paper or electronic, as applicable) the following AEs experienced by a patient and not specified in Section 10.6:

10.621 Grade 2 AEs deemed *possibly, probably, or definitely* related to the study treatment or procedure.

10.622 Grade 3 and 4 AEs regardless of attribution to the study treatment or procedure.

10.623 Grade 5 AEs (Deaths)

10.6231 Any death within 30 days of the patient’s last study treatment or procedure regardless of attribution to the study treatment or procedure.

10.6232 Any death more than 30 days after the patient’s last study treatment or procedure that is felt to be at least possibly treatment related must also be submitted as a Grade 5 AE, with a CTCAE type and attribution assigned.

10.7 Late Occurring Adverse Events

Refer to the instructions in the Forms Packet (or electronic data entry screens, as applicable) regarding the submission of late occurring AEs following completion of the Active Monitoring Phase (i.e., compliance with Test Schedule in Section 4.0).

10.8 **Genentech Additional Event Reporting**

Instructions SAFETY REPORTING OF

ADVERSE EVENTS

10.81 ASSESSMENT OF SAFETY

Specification of Safety Variables

Safety assessments will consist of monitoring and reporting adverse events (AEs) and serious adverse events

(SAEs) per protocol. This includes all events of death, and any study specific issue of concern.

Adverse Events

An AE is any unfavorable and unintended sign, symptom, or disease temporally associated with the use of an investigational medicinal product (IMP) or other protocol-imposed intervention, regardless of attribution.

This includes the following:

- AEs not previously observed in the subject that emerge during the protocol-specified AE reporting period, including signs or symptoms associated with [insert condition being studied] that were not present prior to the AE reporting period.
- Complications that occur as a result of protocol-mandated interventions (e.g., invasive procedures such as cardiac catheterizations)
- If applicable, AEs that occur prior to assignment of study treatment associated with medication washout, no treatment run-in, or other protocol-mandated intervention.
- Preexisting medical conditions (other than the condition being studied) judged by the investigator to have worsened in severity or frequency or changed in character during the protocol-specified AE reporting period.

Serious Adverse Events

An AE should be classified as an SAE if the following criteria are met:

- It results in death (i.e., the AE actually causes or leads to death).
- It is life threatening (i.e., the AE, in the view of the investigator, places the subject at immediate risk of death. It does not include an AE that, had it occurred in a more severe form, might have caused death.).
- It requires or prolongs inpatient hospitalization.
- It results in persistent or significant disability/incapacity (i.e., the AE results in substantial disruption of the subject's ability to conduct normal life functions).
- It results in a congenital anomaly/birth defect in a neonate/infant born to a mother exposed to the IMP.

- It is considered a significant medical event by the investigator based on medical judgment (e.g., may jeopardize the subject or may require medical/surgical intervention to prevent one of the outcomes listed above).

10.82 METHODS AND TIMING FOR ASSESSING AND RECORDING SAFETY VARIABLES

The investigator is responsible for ensuring that all AEs and SAEs that are observed or reported during the study, are collected and reported to the FDA, appropriate IRB(s), and Genentech, Inc. in accordance with CFR312.32 (IND Safety Reports).

Adverse Event Reporting Period

The study period during which all AEs and SAEs must be reported begins after informed consent is obtained and initiation of study treatment and ends 30 days following the last administration of study treatment or study discontinuation/termination, whichever is earlier. After this period, investigators should only report SAEs that are attributed to prior study treatment.

Assessment of Adverse Events

All AEs and SAEs whether volunteered by the subject, discovered by study personnel during questioning, or detected through physical examination, laboratory test, or other means will be reported appropriately. Each reported AE or SAE will be described by its duration (i.e., start and end dates), regulatory seriousness criteria if applicable, suspected relationship to the Atezolizumab and/or Tiragolumab and/or Vemurafenib and/or Cobimetinib (see following guidance), and actions taken.

To ensure consistency of AE and SAE causality assessments, investigators should apply the following general guideline:

Yes

There is a plausible temporal relationship between the onset of the AE and administration of Atezolizumab and/or Tiragolumab and/or Vemurafenib and/or Cobimetinib, and the AE cannot be readily explained by the subject's clinical state, intercurrent illness, or concomitant therapies; and/or the AE follows a known pattern of response to Atezolizumab and/or Tiragolumab and/or Vemurafenib and/or Cobimetinib; and/or the AE abates or resolves upon discontinuation of Atezolizumab and/or Tiragolumab and/or Vemurafenib and/or Cobimetinib or dose reduction and, if applicable, reappears upon re-challenge.

No

Evidence exists that the AE has an etiology other than the Atezolizumab and/or Tiragolumab and/or Vemurafenib and/or Cobimetinib (e.g., preexisting medical condition, underlying disease, intercurrent illness, or concomitant medication); and/or the AE has no plausible temporal relationship to Atezolizumab and/or Tiragolumab and/or Vemurafenib and/or Cobimetinib administration (e.g., cancer diagnosed 2 days after first dose of Atezolizumab and/or Vemurafenib and/or Cobimetinib.

For patients receiving combination therapy, causality will be assessed individually for each protocol-mandated therapy.

Expected adverse events are those adverse events that are listed or characterized in the Package Insert (P.I) or current Investigator Brochure (IB).

Unexpected adverse events are those not listed in the P.I or current I.B or not identified. This includes adverse events for which the specificity or severity is not consistent with the description in the P.I. or I.B. For example, under this definition, hepatic necrosis would be unexpected if the P.I. or I.B. only referred to elevated hepatic enzymes or hepatitis.

10.83 PROCEDURES FOR ELICITING, RECORDING, AND REPORTING ADVERSE EVENTS

Eliciting Adverse Events

A consistent methodology for eliciting AEs at all subject evaluation time points should be adopted. Examples of non-directive questions include:

- "How have you felt since your last clinical visit?"
- "Have you had any new or changed health problems since you were last here?"

Specific Instructions for Recording Adverse Events

Investigators should use correct medical terminology/concepts when reporting AEs or SAEs. Avoid colloquialisms and abbreviations.

a. Diagnosis vs. Signs and Symptoms

If known at the time of reporting, a diagnosis should be reported rather than individual signs and symptoms (e.g., record only liver failure or hepatitis rather than jaundice, asterixis, and elevated transaminases). However, if a constellation of signs and/or symptoms cannot be medically characterized as a single diagnosis or syndrome at the time of reporting, it is acceptable to report the information that is currently available. If a diagnosis is subsequently established, it should be reported as follow-up information.

b. Deaths

All deaths that occur during the protocol-specified AE reporting period (see Section I), regardless of attribution, will be reported to the appropriate parties. When recording a death, the event or condition that caused or contributed to the fatal outcome should be reported as the single medical concept. If the cause of death is unknown and cannot be ascertained at the time of reporting, report "Unexplained Death".

c. Preexisting Medical Conditions

A preexisting medical condition is one that is present at the start of the study. Such conditions should be reported as medical and surgical history. A preexisting medical condition should be re-assessed throughout the trial and reported as an AE or SAE only if the frequency, severity, or character of the condition worsens during the study. When reporting such events, it is important to convey the concept that the preexisting condition has changed by including applicable descriptors (e.g., "more frequent headaches").

d. Hospitalizations for Medical or Surgical Procedures

Any AE that results in hospitalization or prolonged hospitalization should be documented and reported as an SAE. If a subject is hospitalized to undergo a medical or surgical procedure as a result of an AE, the event responsible for the procedure, not the procedure itself, should be reported as the SAE. For example, if a subject is hospitalized to undergo coronary bypass surgery, record the heart condition that necessitated the bypass as the SAE.

Hospitalizations for the following reasons do not require reporting:

- Hospitalization or prolonged hospitalization for diagnostic or elective surgical procedures for preexisting conditions
- Hospitalization or prolonged hospitalization required to allow efficacy measurement for the study or
- Hospitalization or prolonged hospitalization for scheduled therapy of the target disease of the study

e. Assessment of Severity of Adverse Events

The adverse event severity grading scale for the NCI CTCAE (v5.0) will be used for assessing adverse event severity. Below table should be used for assessing severity for adverse events that are not specifically listed in the NCI CTCAE.

Adverse Event Severity Grading Scale for Events Not Specifically Listed in NCI CTCAE

Grade	Severity
1	Mild; asymptomatic or mild symptoms; clinical or diagnostic observations only; or intervention not indicated
2	Moderate; minimal, local, or non-invasive intervention indicated; or limiting age-appropriate instrumental activities of daily living [a]
3	Severe or medically significant, but not immediately life-threatening; hospitalization or prolongation of hospitalization indicated; disabling; or limiting self-care activities of daily living [b,c]
4	Life-threatening consequences or urgent intervention indicated d
5	Death related to adverse event [d]

NCI CTCAE = National Cancer Institute Common Terminology Criteria for Adverse Events.

Note: Based on the most recent version of NCI CTCAE (v4.0), which can be found at: http://ctep.cancer.gov/protocolDevelopment/electronic_applications/ctc.htm

a. Instrumental activities of daily living refer to preparing meals, shopping for groceries or clothes, using the telephone, managing money, etc.

b. Examples of self-care activities of daily living include bathing, dressing and undressing, feeding oneself, using the toilet, and taking medications, as performed by patients who are not bedridden.

c. If an event is assessed as a "significant medical event," it must be reported as a serious adverse event

d. Grade 4 and 5 events must be reported as serious adverse events

f. Pregnancy

If a female subject becomes pregnant while receiving the Atezolizumab and/or Tiragolumab and/or Vemurafenib and/or Cobimetinib or within 90 days after the last dose of and/or Vemurafenib and/or Cobimetinib or within 5 months after the last dose of Atezolizumab and/or Tiragolumab and/or Vemurafenib, a report should be completed and expeditiously submitted to Genentech, Inc. Follow-up to obtain the outcome of the pregnancy should also occur. Abortion, whether accidental, therapeutic, or spontaneous, should always be classified as serious, and expeditiously reported as an SAE. Similarly, any congenital anomaly/birth defect in a child born to a female subject exposed to Atezolizumab and/or Tiragolumab should be reported as an SAE.

- **Pregnancies in Female Partners of Male Patients**

Male patients will be instructed through the Informed Consent Form to immediately inform the investigator if their partner becomes pregnant during the study or within

30 days after the last dose of study drug.

g. Post-Study Adverse Events

The investigator after the end of the adverse event reporting period (defined as 30 days after the last dose of study drug) should report all deaths, (regardless of cause), and any serious adverse event including development of cancer or a congenital anomaly in a subsequently conceived offspring of a female subject including pregnancy occurring in the partner of a male study subject who participated in the study that is believed to be related to prior exposure to Atezolizumab and/or Tiragolumab.

Reconciliation will be performed by both parties during this period Half-Yearly to ensure successful transmission of Single case reports

h. Product complaints

A Product Complaint is defined as any written or oral information received from a complainant that alleges deficiencies related to identity, quality, safety, strength, purity, reliability, durability, effectiveness, or performance of a product after it has been released and distributed to the commercial market or clinical trial.

i. Other Special Situations Reports

The following other Special Situations Reports should be collected even in the absence of an Adverse Event and transmitted to Genentech:

- Data related to the Product usage during breastfeeding
- Data related to overdose, abuse, misuse or medication error (including potentially exposed or intercepted medication errors)
- In addition, reasonable attempts should be made to obtain and submit the age or age group of the patient, in order to be able to identify potential safety signals specific to a particular population

j. Reconciliation

The Sponsor agrees to conduct reconciliation for the product. Genentech and the Sponsor will agree to the reconciliation periodicity and format, but agree at minimum to exchange quarterly line listings of cases received by the other party.

The periodic line-listing will be exchanged within seven (7) calendar days of the end of the agreed time period. Confirmation of receipt should be received within the time period mutually agreed upon.

If discrepancies are identified, the Sponsor and Genentech will cooperate in resolving the discrepancies. The responsible individuals for each party shall handle the matter on a case-by-case basis until satisfactory resolution. The sponsor shall receive reconciliation guidance documents within the ‘Activation Package’.

Following Case Transmission Verification, single case reports which have not been received by Genentech shall be forwarded by Sponsor/Investigator to Genentech within five (5) calendar days from request by Genentech.

At the end of the study, a final cumulative Case Transmission Verification report will be sent to Genentech

10.85 Adverse Event Reporting

<u>Exchange of Single Case Reports</u>

The Investigator will be responsible for collecting all protocol-defined Adverse Events (AEs)/Serious Adverse Events (SAEs), AEs of Special Interest (AESIs), Special Situation Reports (including pregnancy reports) and Product Complaints (with or without an AE) originating from the Study for the Product.

Investigators must report all the above-mentioned single case reports adequately to Genentech within one (1) business day of the awareness datet. The completed MedWatch or CIOMS I form or Genentech approved reporting form should be faxed/emailed immediately upon completion to Genentech Drug Safety at the following contacts

All protocol-defined AEs, SAEs, AESIs, Special Situation Reports (including pregnancy reports) and Product Complaints with an AE should be sent to:

Fax: 650-238-6067
Email: usds_aereporting-d@gene.com

All Product Complaints *without* an AE should call via:

PC Hotline Number: (800) 334-0290 (M-F : 5 am to 5 pm PST)

It is understood and agreed that the Sponsor will be responsible for the evaluation of AEs/SAEs, AESIs, Special Situation Reports (including pregnancy reports) and Product Complaints (with or without an AE) originating from the study.

These single case reports will be exchanged between the parties as outlined below so that regulatory obligations are met.

Serious adverse events (SAEs), AEs of special interest (AESIs), pregnancy reports (including pregnancy occurring in the partner of a male study subject), other Special Situation Reports, and Product Complaints (with or without an AE), where the patient has been exposed to the Genentech Product, will be sent on a MedWatch form or CIOMS I form or on Genentech approved reporting forms to Genentech Drug Safety.

MEDWATCH 3500A REPORTING GUIDELINES

In addition to completing appropriate patient demographic and suspect medication information, the report should include the following information within the Event Description (section 5) of the MedWatch 3500A form:

- Protocol description (and number, if assigned)
- Description of event, severity, treatment, and outcome if known
- Supportive laboratory results and diagnostics
- Investigator's assessment of the relationship of the adverse event to each investigational product and suspect medication

Follow-Up Information

Additional information may be added to a previously submitted report by any of the following methods:

- Adding to the original MedWatch 3500A report and submitting it as follow-up
- Adding supplemental summary information and submitting it as follow-up with the original
- MedWatch 3500A form
- Summarizing new information and faxing it with a cover letter including patient identifiers (i.e. D.O.B. initial, patient number), protocol description and number, if assigned, brief adverse event description, and notation that additional or

follow-up information is being submitted (The patient identifiers are important so that the new information is added to the correct initial report)

Occasionally Genentech may contact the reporter for additional information, clarification, or current status of the patient for whom an adverse event was reported. For questions regarding SAE reporting, you may contact Genentech Drug Safety or the MSL assigned to the study. Relevant follow-up information should be submitted to Genentech Drug Safety as soon as it becomes available and/or upon request.

MedWatch 3500A (Mandatory Reporting) form is available at http://www.fda.gov/downloads/AboutFDA/ReportsManualsForms/Forms/UCM048334.pdf

Additional Reporting Requirements for IND Holders (if applicable):

For Investigator-Initiated IND Studies, some additional reporting requirements for the FDA apply in accordance with the guidance set forth in 21 CFR §600.80 expedited IND Safety Reports according to the following guidance and timelines:

7 Calendar Day Telephone or Fax Report:

The Investigator is required to notify the FDA of any fatal or life-threatening adverse event that is unexpected and assessed by the Investigator to be possibly related to the use of Atezolizumab and/or Tiragolumab and/or Vemurafenib and/or Cobimetinib. An unexpected adverse event is one that is not already described in the Atezolizumab and/or Tiragolumab and/or Vemurafenib and/or Cobimetinib Investigator Brochure. Such reports are to be telephoned or faxed to the FDA and Genentech within 7 calendar days of first learning of the event.

15 Calendar Day Written Report

The Investigator is also required to notify the FDA and all participating investigators, in a written IND Safety Report, of any serious, unexpected AE that is considered reasonably or possibly related to the use of Atezolizumab and/or Tiragolumab and/or Vemurafenib and/or Cobimetinib. An unexpected adverse event is one that is not already described in the Atezolizumab and/or Tiragolumab and/or Vemurafenib and/or Cobimetinib investigator brochure.

Written IND Safety reports should include an Analysis of Similar Events in accordance with regulation 21 CFR§ 312.32. All safety reports previously filed by the investigator with the IND concerning similar events should be analyzed and the significance of the new report in light of the previous, similar reports commented on.

Written IND safety reports with Analysis of Similar Events are to be submitted to the FDA, Genentech, and all participating investigators within 15 calendar days of first learning of the event. The FDA prefers these reports on a MedWatch 3500 form, but alternative formats are acceptable (e.g., summary letter).

FDA fax number for IND Safety Reports:

Fax: 1 (800) FDA 0178

All written IND Safety Reports submitted to the FDA by the Investigator must also be faxed to

Genentech Drug Safety:

Fax: (650) 225-4682 or (650) 225-4630
Email: usds_aereporting-d@gene.com

And to the Site IRB:

Submit to Mayo Clinic Cancer Center CRO Safety for submission to Mayo Clinic IRB.

For questions related to safety reporting, please contact Genentech Drug Safety:
Tel: (888) 835-2555
Fax: (650) 225-4682 or (650) 225-4630

IND ANNUAL REPORTS

Copies to Genentech:
All IND annual reports submitted to the FDA by the Sponsor-Investigator should be copied to Genentech. Copies of such reports should be emailed to Genentech at:
Genentech Drug Safety CTV mail box: ctvist_drugsafety@gene.com

AGGREGATE REPORTS

Sponsor/Investigator will forward a copy of the Publication to Genentech upon completion of the Study.

Sponsor/Investigator, as the Sponsor of the Study, will be responsible for the preparation of their own Development Safety Update Report (DSUR) for the Study and for the submission of the report to the regulatory authorities and Ethics Committees of the concerned Member States, where applicable Sponsor/Investigator, agrees to share a copy of their own DSUR with Genentech as soon as reasonably possible after completion. Genentech agrees to forward to Sponsor/Investigator an executive summary of the Genentech DSUR upon request. Furthermore, Genentech agrees that Sponsor/Investigator may cross-reference the executive summary of the Genentech DSUR, as applicable.

STUDY CLOSE-OUT

Any study report submitted to the FDA by the Sponsor-Investigator should be copied to Genentech. This includes all IND annual reports and the Clinical Study Report (final study report). Additionally, any literature articles that are a result of the study should be sent to Genentech. Copies of such reports should be mailed to the assigned Clinical Operations contact for the study:

Atezolizumab IIS Clinical Operations

Email: anti-pdl-1-mpd3280a-gsur@gene.com

QUERIES

Queries related to the Study will be answered by Sponsor-Investigator. However, responses to all safety queries from regulatory authorities or for publications will be discussed and coordinated between the Parties. The Parties agree that Genentech shall have the final say and control over safety queries relating to the Product. Sponsor-Investigator agrees that it shall not answer such queries from regulatory authorities and other sources relating to the Product independently but shall redirect such queries to Genentech.

Both Parties will use all reasonable effort to ensure that deadlines for responses to urgent requests for information or review of data are met. The Parties will clearly indicate on the request the reason for urgency and the date by which a response is required.

SAFETY CRISIS MANAGEMENT

In case of a safety crisis, e.g., where safety issues have a potential impact on the indication(s), on the conduct of the Study, may lead to labeling changes or regulatory actions that limit or restrict the way in which the Product is used, or where there is media involvement, the Party where the crisis originates will contact the other Party as soon as possible.

The Parties agree that Genentech shall have the final say and control over safety crisis management issues relating to the Product. Sponsor-Investigator agrees that it shall not answer such queries from media and other sources relating to the Product but shall redirect such queries to Genentech.

11.0 Treatment Evaluation

11.1 Neoadjuvant phase

Physical examination and history will be performed at each cycle evaluation. Documentation of the size and location of the affected nodes (if palpable) is required at each visit. Prior to operation imaging will be repeated as clinically indicated and consist of PET-CT (required) with or without ultrasound of the affected regional nodal basin or basins.

11.2 Surgery

At operation the resected lymph nodes will be evaluated by permanent section histology. Treatment response will be assessed by histopathology evaluation. All surgical pathology reports must contain the following information: number of positive lymph nodes, total number of lymph nodes removed, diameter of largest diameter in mm and a comment regarding the presence or absence of signs of treated tumor, e.g., necrosis, histiocytosis and fibrosis per lymph node removed.

11.3 Adjuvant treatment phase

Patients will undergo physical exam at each evaluation during treatment and observation. Patients will undergo PET/CT imaging every 12 weeks during adjuvant treatment and observation. Recurrences will be documented.

12.0 Descriptive Factors

12.1 Disease status: recurrent nodal disease vs. concurrent diagnosis of primary melanoma with nodal involvement

12.2 Location: 1 nodal basin vs. >1 nodal basin

12.3 Baseline tissue PD-L1 staining: None versus low versus high

12.4 BRAF mutation status: wild type or mutated

13.0 Treatment/Follow–up Decision at Evaluation of Patient

13.1 Neoadjuvant phase

13.11 Patients who have not had progression of disease or intolerable toxicity may continue on study per protocol.

13.12 Patients who have not had disease progression but develop intolerable toxicity during neoadjuvant treatment may be eligible for retreatment at a lower dose (Section 8.0) or proceed to surgery.

13.13 Patients who progress during neoadjuvant treatment and are still surgical candidate may proceed to surgery.

13.14 Patients who progress during neoadjuvant treatment and are no longer a surgical candidate will complete on-study, neoadjuvant treatment, and end of protocol treatment form. Survival follow-up is required. Subsequent treatment is at the discretion of the patient’s medical team.

13.15 Patients who are no longer a surgical candidate after discontinuation of neoadjuvant treatment for reasons other than disease progression will complete on-study, neoadjuvant treatment, and end of protocol treatment form. Survival follow-up is required. Subsequent treatment is at the discretion of the patient’s medical team.

13.16 Patients who discontinue neoadjuvant treatment and are refusing all remaining protocol treatment (that is, surgery and adjuvant treatment) will complete on-

study, neoadjuvant treatment, and end of protocol treatment form. Survival follow-up is required. Subsequent treatment is at the discretion of the patient's medical team.

13.17 Patients who discontinue neoadjuvant treatment due to other medical conditions will complete on-study, neoadjuvant treatment, and end of protocol treatment form. Survival follow-up is required. Subsequent treatment is at the discretion of the patient's medical team.

13.2 Surgery

Patients who were not rendered NED will go to the survival follow-up (event monitoring) phase of the study where progression and survival information will be collected every 6 months for a maximum of 5years post-registration. Subsequent treatment is at the discretion of the patient's medical team.

Patients who are rendered NED but refuse to begin the protocol-specific adjuvant treatment will go survival follow-up (event monitoring) phase of the study where progression and survival information will be collected every 3 months until disease progression and then every 6 months for a maximum of 5 years post-registration. Subsequent treatment is at the discretion of the patient's medical team

13.3 Adjuvant Treatment Phase

13.31 Patients who have not had disease recurrence and have not developed intolerable toxicity may continue treatment per protocol

13.32 Patients who have not had a disease recurrence but develop intolerable toxicity or are refusing further protocol treatment will go to observation phase of the study.

13.33 Patients who discontinue adjuvant treatment due to disease progression or other medical condition will go to the survival follow-up (event monitoring) period where disease and survival information will be collected every 3 months until disease progression and then every 6 months for a maximum of 5 years post registration.

13.34 Patients who have completed 8 cycles of adjuvant treatment will enter the observation phase of the study.

13.4 Observation Phase

Patients who discontinue observation phase due to refusal, disease progression, or initiation of a non-protocol anti-cancer therapy will go to the survival follow-up (event monitoring) period where disease and survival information will be collected every 3 months until disease progression and then every 6 months for a maximum of 5 years post registration.

13.5 Ineligible

A patient is deemed *ineligible* if after registration, it is determined that at the time of registration, the patient did not satisfy each and every eligibility criteria for study entry.

Protocol treatment is to be discontinued. No further follow-up is required. Subsequent treatment is at the discretion of the patient's medical team.

- If the patient received treatment, all data up until the point of confirmation of ineligibility must be submitted.
- If the patient never received treatment, on-study material must be submitted.

13.6 Cancel

A patient is deemed a *cancel* if he/she is removed from the study for any reason before any study treatment is given. On-study material and the End of Active Treatment/Cancel Notification Form must be submitted. No further data submission is necessary.

14.0 Body Fluid Biospecimens

14.1 Summary Table of Research Blood and Body Fluid Specimens to be Collected for this Protocol

Correlative Study (Section 14.4 for more information)	Mandatory or Optional	Blood or Body Fluid being Collected	Type of Collection Tube (color of tube top)	Volume to collect per tube (# of tubes to be collected)	After registration <u>and</u> prior to start of treatment	After each cycle of neoadj treatment	At end of all neoadj treatment	At time of surgery	≤7 days prior to start of adjuvant treatment	At end of the 4th and 8th Cycles of Adjuvant Tx and every 3 months during observation and at disease progression	Process at site? (Yes or No)	Temperature Conditions for Shipping /Storage
Bim, T cell receptor sequencing, mass cytometry, and sPD-L1 studies	Mandatory	Plasma, Peripheral blood mono-nuclear cells (PBMCs)	Sodium Heparin (Green)	10 mL(5)	X	X	X		X	X	No	Ambient for initial transport/ ≤-65°C for storage
cfDNA mutation testing	Mandatory	Blood	Foundation ACT tubes	10 mL (2)	X		X		X		No	Ambient for initial transport
Microbiome	Mandatory	Stool	Stool collection kit	N/A	X		X			X[55]	No	Frozen
	Mandatory	Buccal (cheek) swab	Swab collection kit	2 swabs	X		X				No	Frozen
	Mandatory	Skin swab	Swab collection kit	30 strokes	X		X	X[56]			No	Frozen
RNA-Seq†	Mandatory	Frozen tissue	N/A	N/A				X			No	Frozen

†NOTE: RNA-Seq is described in Section 17.3 with other tissue (it is included here because frozen tissue is collected/shipped through BAP)

---

[55] Microbiome stool collection ≤2 weeks after last dose of study drug and at time of disease progression

[56] Skin swab only is collected during surgery

14.2 Collection and Processing

14.21 Blood

Blood samples will be collected at the clinical phlebotomy areas in the Mayo Clinic and University of Minnesota. Peripheral blood must be kept at room temperature until processing.

Sites outside of Mayo Clinic Rochester must ship immediately overnight (on the same day drawn) at ambient temperature per Section 14.3.
All blood samples will be routed to:

Dr Matthew S Block's Laboratory
Attn: Courtney Erskine
Guggenheim 3-23
Mayo Clinic
200 First St SW
Rochester MN 55905

At Mayo Clinic in Rochester, sodium heparin blood will be processed over Ficoll to separate PBMCs from plasma. Processed PBMCs and plasma will be stored in Dr. Block's laboratory: Attn: Courtney Erskine, Guggenheim 3-23, Mayo Clinic, Rochester, MN 55905.

14.22 Microbiome

Kits will be provided.

Follow instructions in each kit.

Ship all items frozen per kit instructions.

Samples will be processed and stored in Dr Block's lab, Guggenheim 3-23, Mayo Clinic, 200 First St SW, Rochester MN 55905

14.3 Shipping and handling

14.31 Kits will be provided.

14.32 Shipping Specimens (sites outside of Mayo Clinic Rochester, MN only)

Shipping of the specimens will be requested at completion of each stage of enrollment. Please notify the principal investigators (Drs. Block and Hieken) prior to shipping.

14.322 Stool, buccal, and skin specimens will be shipped according to kit instructions.

14.321 Blood specimens from AZ/FL/UofM will be shipped to:

Laboratory of Matthew S Block MD PhD
Attn: Courtney Erskine, Guggenheim 3-23
Mayo Clinic
200 First St SW
Rochester, MN 55902

14.33 Handling Specimens

Microbiome samples will be processed and stored in Dr. Block's Laboratory.

Blood samples will be transferred to Dr Block's lab for processing and storage.

14.4 Background and methodology

14.41 Bim assays

The pro-apoptotic molecule Bim is upregulated in tumor-related T cells upon engagement of PD-1. We have demonstrated that high levels of Bim on pre-treatment tumor-related T cells (reflective of a high level of PD-1 engagement) are associated with clinical responses to anti-PD-1(Dronca 2016).

Using our previously published flow cytometry technique (Dronca 2016), we will label cells with CD8, CD11b, PD-1, and intracellular Bim. Tumor-related T cells will be defined as CD8+11b+PD-1+. We will determine the frequency of Bim+ tumor-related T cells both prior to treatment and after neoadjuvant therapy, as well as before and after adjuvant therapy. We will correlate baseline and changes in Bim with RFS and pCR.

14.42 Soluble PD-L1 assays

In addition to being expressed as a cell surface ligand in the tumor microenvironment, PD-L1/B7-H1 can be secreted and measured in plasma; soluble PD-L1 (sPD-L1) is associated with prognosis in several tumor types (Frigola 2011). Moreover, in contrast to intratumoral PD-L1, sPD-L1 is associated with a lack of responsiveness to anti-PD-1 therapy in melanoma (Dronca RS et al 2017, and manuscript in preparation).

Using our previously published enzyme-linked immunosorbent assay (ELISA) for sPD-L1 (Frigola 2011), we will quantitate sPD-L1 concentrations in patient plasma prior to initiation of neoadjuvant therapy and after neoadjuvant therapy. We will correlate baseline sPD-L1 concentration and the change in PD-L1 concentration from baseline to post-neoadjuvant therapy with response at the time of surgery (pCR versus no pCR) and with RFS.

14.43 Microbiome Studies

Alteration in the gut microbiome has been reported in association with response to immune checkpoint inhibition in both animal models and preliminary reports from human neoadjuvant melanoma trials (Sivan 2015, Wargo 2017). To explore microbiome associations with targeted and immunotherapy response we will collect stool, buccal, skin, and tissue samples before and after treatment. 16S rRNA hypervariable tag sequencing (Jumpstart 2012) will be done to generate microbiome taxa profiles using the DNA samples to partially amplify the microbial (V3-V5 region) 16S rRNA gene through PCR. The PCR product will be purified and quantified before sequencing. Barcoding samples prior to sequencing will yield >20,000 reads/samples, ensuring detection of both dominant (core microbiome) and poorly represented taxa (variable microbiome). We will use quantitatively optimal protocols (Sipos 2010). Reads will be aligned using custom multiple alignment tools (TORNADO v2.0) that merge paired end reads into a single multiple alignment and obtains taxa calls for correlation analysis and utilizing bioinformatic pipelines such as QIIME (Jeraldo 2014, Caporaso, Kuczynski 2012, Caporoso, Lauber 2012). We will assess the proportion of samples with a given organism present (at abundance >0.1%) and report relative abundance and diversity indices in association with treatment response and other variables of interest.

14.44 Cell-free DNA (cfDNA)

To assess for changes in circulating tumor DNA, blood samples from patients will be sent to Foundation Medicine for FoundationACT testing. Briefly, multiple commonly mutated genes will be assessed qualitatively and quantitatively in patient cfDNA via multigene sequencing.
Foundation Medicine
150 Second Street
Cambridge, MA 02141

14.45 T cell receptor sequencing

T cells undergo somatic gene rearrangement of the T cell receptor Valpha and Vbeta regions during differentiation. In this way, each T cell clone has a uniquely rearranged T cell receptor; this allows different T cells to recognize distinct antigens. By performing sequencing of individual T cell receptor Valpha and/or Vbeta regions, the diversity of T cell clones in a given tissue, as well as the degree of clonal expansion of individual clones, can be determined. To assess for the diversity and clonality of T cell receptors in peripheral blood, processed PBMCs (at least 5×10^5) will be sent to Adaptive Biotechnologies for T cell receptor sequencing.

Adaptive Biotechnologies
1551 Eastlake Ave E, Ste 200
Seattle, WA 98102

In addition, by stimulating PBMCs with candidate neoantigen peptides, we can identify TCR sequences from neoantigen-specific T cells and track the neoantigen-specific T cells at each time point in both blood and tissue. First, somatic mutations will be identified by comparing whole exome sequencing of tumor with whole genome sequencing of blood. Patient HLA class I and class II alleles will be identified from sequencing results as well. Peptides spanning each somatic mutation will be assessed in silico for predicted binding to patient-specific HLA alleles. Peptides that a) contain at least one somatic mutation, and b) are predicted to bind to one of the patient's HLA alleles will be considered candidate neoantigens. Candidate neoantigen peptides will be used to stimulate PBMCs, and TCR sequences from peptide-activated PBMCs will be considered neoantigen-specific TCRs.

14.46 Peripheral blood immune phenotype analyses with mass cytometry

Mass cytometry allows quantitation of multiple immune cell subsets in peripheral blood via multiparametric analysis using heavy metal-conjugated monoclonal antibodies to cell surface and intracellular markers if immune cell differentiation. We will perform mass cytometry (CyTOF™) to assess for changes in peripheral blood immune cell subsets that occur during neoadjuvant therapy, after surgery, and during adjuvant therapy. Analyses will be performed by the Mayo Clinic Immune Monitoring Core Facility.

15.0 Drug Information

15.1 Atezolizumab (MPDL3280A, RO5541267, Tecentriq®)

15.11 Background

Atezolizumab is a human immunoglobulin (IgG1) monoclonal antibody that is produced in Chinese hamster ovary (CHO) cells. Atezolizumab targets programmed death-ligand 1 (PD-L1) on immune cells or tumor cells and prevents interaction with either programmed death−1 (PD-1) receptor or B7.1 (CD80), both of which function as inhibitory receptors expressed on T cells. Interference of the PD-L1:PD-1 and PD-L1:B7.1 interactions may enhance the magnitude and quality of the tumor-specific T-cell response through increased T-cell priming, expansion, and/or effector function. Atezolizumab shows anti-tumor activity in various nonclinical models and is being investigated as a potential therapy for cancer patients with locally advanced or metastatic malignancies.

15.12 Formulation

Atezolizumab is supplied in a single use vial as a colorless-to-slightly-yellow, sterile, preservative-free clear liquid solution intended for IV administration. The vial is designed to deliver 20 mL (1200 mg) of atezolizumab solution but may contain more than the stated volume to enable delivery of the entire 20 mL volume. The atezolizumab drug product is formulated as 60 mg/mL atezolizumab in 20 mM histidine acetate, 120 mM sucrose, 0.04% polysorbate 20, pH 5.8.

15.13 Preparation and storage

Atezolizumab must be refrigerated at 2°C−8°C (36°F−46°F) upon receipt until use. No preservative is used in atezolizumab drug product; therefore, the vial is intended for single use only. Discard any unused portion of drug left in a vial. Vial contents should not be frozen or shaken and should be protected from direct sunlight. Atezolizumab is administered using 0.9% sodium chloride 250 mL IV bags and infusion lines equipped with 0.2 micron in-line filters (filter membrane of polyethersulfone [PES]). Bags may be constructed of polyvinyl chloride (PVC), polyethylene, or polyolefin (PO). The final diluted concentration must be between 2.4 mg/mL and 9.6 mg/mL. For flat or fixed dosing (e.g. 800 mg, 840 mg, or 1200 mg) in 250 mL IV infusion bags, the prepared solution of atezolizumab may be stored at 2°C−8°C (36°F−46°F) for 24 hours or at ambient temperature ≤25°C (77°F) for 8 hours. For weight-based dosing using a 250-mL IV bag or smaller, the dose solution may be stored for 24 hours at 2°C−8°C (36°F−46°F) including no more than 4 hours at room temperature prior to administration. If the dose solution is stored at 2°C−8°C (36°F−46°F), it should be removed from refrigeration and allowed to reach room temperature prior to administration. Do not shake or freeze infusion bags containing the dose solution.

15.14 Administration

The initial dose of atezolizumab will be delivered over 60 (±15) minutes. If the first infusion is tolerated without infusion-associated adverse effects, the second infusion may be delivered over 30 (± 10) minutes. If the 30-minute infusion is well tolerated, all subsequent infusions may be delivered over 30 (± 10) minutes.

15.15 Pharmacokinetic

information Distibution: V_{dss}:

6.9 L

Half-life elimination: 27 days
Hepatic Impairment: No clinically relevant effect with mild hepatic impairment. The effect of moderate or severe hepatic impairment on the pharmacokinetics of atezolizumab is unknown.
Renal Impairment: No clinically relevant effect with renal impairment.

15.16 Potential Drug Interactions

No formal PK drug-drug interaction studies have been conducted with atezolizumab. The drug interaction potential of atezolizumab is unknown.

15.17 Known potential adverse events

Consult the package insert and investigator's brochure for the most current and complete information.

Common known potential adverse events, >10%:
Cardiovascular: Peripheral edema
Central nervous system: Fatigue
Dermatologic: Skin rash, pruritus
Endocrine & metabolic: Hyponatremia
Gastrointestinal: Decreased appetite, nausea, constipation, colitis, diarrhea, abdominal pain, vomiting
Genitourinary: Urinary tract infection, hematuria
Hematologic & oncologic: Lymphocytopenia
Infection: Infection
Neuromuscular & skeletal: Back pain, neck pain, arthralgia
Respiratory: Dyspnea, cough
Miscellaneous: Fever

Less common known potential adverse events, 1% - 10%:
Cardiovascular: Venous thromboembolism
Central nervous system: Guillain-Barre syndrome, meningoencephalitis, myasthenia, myasthenia gravis, confusion
Endocrine & metabolic: Hyperglycemia, hypothyroidism, hypoalbuminemia, hyperthyroidism
Gastrointestinal: Increased serum amylase, increased serum lipase, pancreatitis, intestinal obstruction
Genitourinary: Urinary tract obstruction
Hematologic & oncologic: Anemia
Hepatic: Increased serum ALT/AST, hepatitis, increased serum alkaline phosphatase, serum bilirubin
Infection: Sepsis
Ophthalmic: Intraocular inflammation
Renal: Increased serum creatinine, acute renal failure
Respiratory: Pneumonitis, pneumonia
Miscellaneous: Infusion related reaction

Rare known potential adverse events, <1% (Limited to important or life-threatening):

Adrenocortical insufficiency, diabetes mellitus (with ketoacidosis), hypophysitis

15.18 Drug procurement

Supplied by Genentech to each site.

Each participating treating location will be responsible for monitoring the supply and will use the Drug Order Request Form to order additional supplies as needed.

Outdated or remaining drug is to be destroyed on-site per procedures in place at each institution.

15.19 Nursing Guidelines

15.191 Anti PD-L1 side effects vary greatly from those of traditional chemotherapy and can vary in severity from mild to life threatening. Instruct patients to report any side effects to the study team immediately. Side effects may be immediate or delayed up to months after discontinuation of therapy. Most side effects are reversible with prompt intervention of corticosteroids

15.192 Diarrhea can be common and can be very severe, leading to colonic perforation. Instruct patients to report ANY increase in the number of stools and/or change in baseline, blood in the stool, abdominal pain to the study team immediately.

15.193 Rash/pruritus/dermatitis is seen. Patients should report any rash to the study team. Treat per section 8.0 and monitor for effectiveness.

15.194 Monitor LFTs closely as elevations in these levels could indicate early onset autoimmune hepatitis. Patients should also be instructed to report any jaundice, or right upper quadrant pain to the study team immediately.

15.195 Pneumonitis can be seen and may be mild (only seen on imaging) to severe. Patients should be instructed to report any SOB, dyspnea, cough, chest pain, etc. to the study team immediately. Patients reporting these symptoms should have a pulse ox checked and consider immediate imaging per the treating MD.

15.196 Endocrinopathies (including hypopituitarism, hypothyroidism, hypophysitis, and adrenal insufficiency) are a concern given the mechanism of action of this agent. Patients may present only with the vague sense of fatigue and “not feeling well”. Additional symptoms may be that of nausea, sweating and decreased activity tolerance. Instruct patients to report these signs or symptoms immediately and obtain appropriate labs as ordered by MD.

15.197 Pancreatitis is possible with anti PD-L1 therapy based on mechanism of action. Instruct patients to report abdominal pain, nausea and vomiting to the study team.

15.198 Patients who are started on steroid therapy for any side effects of anti PD-L1 toxicity should be instructed to take the steroids as ordered, and not to discontinue abruptly as symptoms may return and be severe. Patients may be on steroid therapy for weeks. Instruct patients to report any increase or change in side effects with any dosage decrease as patients may need a slower taper.

15.2 Cobimetinib (Cotellic®) (RO5514041)

15.21 Background

Cobimetinib is a kinase inhibitor indicated for the treatment of patients with unresectable or metastatic melanoma with a BRAF V600E or V6060K mutation, in combination with vemurafenib.

15.22 Formulation

Cobimetinib is available as 20 mg film coated tablets. Each cobimetinib tablet contains 22 mg cobimetinib fumerate (which corresponds to 20 mg of the cobimetinib free base) and the following inactive ingredients: Tablet core: microcrystalline cellulose, lactose monohydrate, croscarmellose sodium, magnesium stearate. Coating: polyvinyl alcohol, titanium dioxide, polyethylene glycol 3350, talc. Cobimetinib is available as bottles of 63 tablets.

15.23 Storage

Store at room temperature at 59°F−86°F (15°C−30°C).

15.24 Administration

Cobimetinib is taken orally once daily Days 1 through 21 of a 28-day cycle with or without food. If a dose of cobimetinib is missed (i.e., 6 hours or more late for cobimetinib), do not make up that dose; resume dosing with the next scheduled dose.

15.25 Pharmacokinetic information

Absorption: Bioavailability was 46% (90 CI 40-53%) in healthy subjects. A high-fat meal had no effect on cobimetinib AUC and Cmax after a single 20 mg cobimetinib dose in healthy subjects.
Distribution: 95% bound to human plasma proteins. Vd 806 L.
Metabolism: CYP3A oxidation and UGT2B7 glucuronidation.
Half-life, elimination: 44 hours (range 23-70).

Time to peak: Median time to Tmax 2.4 (range 1-24) hours.
Excretion: Feces (76%; ~7 as unchanged drug); Urine (~18%; ~2% as unchanged drug)

15.26 Potential Drug Interaction

Coadministration of cobimetinib with itraconazole (a strong CYP3A4 inhibitor) increased cobimetinib systemic exposure by 6.7-fold. Avoid concurrent use of cobimetinib and strong or moderate CYP3A inhibitors.

Coadministration of cobimetinib with a strong CYP3A inducer may decrease cobimetinib systemic exposure by more than 80% and reduce its efficacy. Avoid concurrent use of cobimetinib and strong or moderate CYP3A4 inducers including but not limited to carbamazepine, efavirenz, phenytoin, rifampin, and St. John's wort.

15.27 Known potential adverse events

Greater than 10%:
Cardiovascular: Decrease left ventricular ejection fraction, hypertension
Dermatologic: Skin photosensitivity, acneiform eruption
Endocrine & metabolic: Hypophosphatemia, increased gamma-glutamyl transferase, hypoalbuminemia, hyponatremia, hyperkalemia, hypokalemia, hypocalcemia
Gastrointestinal: Diarrhea, nausea, vomiting, stomatitis
Hematologic & oncologic: Lymphocytopenia, anemia, thrombocytopenia, hemorrhage (includes bruise, eccymoses, epistaxis, gingival hemorrhage, hematemesis, hematochezia, hemoptysis, hemorrhoidal bleeding, hypermenorrhea, melena, menometrorrhagia, nail bed bleeding, pulmonary hemorrhage, purpura, rectal hemorrhage, rupture of ovarian cyst, subarachnoid

hemorrhage, subgaleal hematoma, traumatic hematoma, uterine hemorrhage, and vaginal hemorrhage)
Investigations: Increased AST, increased serum alkaline phosphatase, increased serum ALT
Neuromuscular & skeletal: Increased creatinine phosphokinase
Ophthalmic: Visual impairment, chorioretinopathy, retinal detachment
Renal: Increased serum creatinine
Respiratory: Cough
Miscellaneous: Fever

1 to 10%:
Central nervous system: Chills
Dermatologic: Skin rash
Gastrointestinal: Gastrointestinal hemorrhage
Genitourinary: Genitourinary tract hemorrhage, hematuria
Hematologic & oncologic: Keratoacanthoma, squamous cell carcinoma of the skin, basal cell carcinoma
Hepatic: Abnormal bilirubin levels

Less than 1% (limited to important or life-threatening): Cerebral hemorrhage, malignant melanoma (second primary), malignant neoplasm (non-cutaneous)

15.28 Drug procurement

Supplied by Genentech to each site.

Each participating treating location will be responsible for monitoring the supply and will use the Drug Order Request Form to order additional supplies as needed.

Outdated or remaining drug is to be destroyed on-site per procedures in place at each institution.

15.29 Nursing Guidelines:

15.291 Agent can cause decreased LVEF function. Instruct patients to report any lower extremity swelling, shortness of breath, and/or chest pain to study team.

15.292 Diarrhea and other gastrointestinal side effects are common. Treat symptomatically and monitor for effectiveness of intervention.

15.293 Rash is common and is usually acneiform in nature. Instruct patient to report any rash immediately.

15.294 Cytopenias can be seen. Monitor CBC w/diff closely. Instruct patient to report any signs or symptoms of infection and/or unusual bruising or bleeding to the study team,

15.295 Bleeding including serious and/or fatal hemorrhage can be seen. Instruct patient to report any bleeding to study team.

15.296 Monitor LFTs.

15.297 Rarely interstitial lung disease can be seen. Instruct patient to report any cough, dyspnea to the study team.

15.298 Rhabdomyolysis is rare but serious side effect of this agent. Instruct patients to report any weakness, muscle pain, or just feelings of unwellness to the study team immediately.

15.299a Instruct patient that it is important to only take agent on Days 1-21 of the cycle. If any days in the 21 days are missed, they should not be made up and patients should cease taking agent on Day 21.

15.299b Rarely ocular complications can occur, including chorioretinopathy, retinal detachment and other visual disturbances. Instruct patients to report any visual disturbances and/or eye pain to the study team immediately.

15.299c In combination with vemurafenib, patients may experience pyrexia. Instruct patients to report any fever or chills to the study team.

15.3 **Vemurafenib** (Zelboraf®) (RO5185426, PLX4032)

15.31 Background

Vemurafenib is a low molecular weight, orally available, inhibitor of some mutated forms of BRAF serine-threonine kinase, including BRAF V600E. Vemurafenib also inhibits other kinases in vitro such as CRAF, ARAF, wild-type BRAF, SRMS, ACK1, MAP4K5 and FGR at similar concentrations. Some mutations in the BRAF gene including V600E result in constitutively activated BRAF proteins, which can cause cell proliferation in the absence of growth factors what would normally be required for proliferation. Vemurafenib has anti-tumor effects in cellular and animal models of melanomas with mutated BRAF V600E.

15.32 Formulation

Vemurafenib is available as 240 mg film coated tablets. Each vemurafenib tablet contains 240 mg vemurafenib and inactive ingredients include: Tablet core: hypromellose acetate succinate, croscarmellose sodium, colloidal silicon dioxide, magnesium stearate, and hydroxypropyl cellulose. Coating: pinkish white: poly (vinyl alcohol), titanium dioxide, polyethylene glycol 3350, talc, and iron oxide red. Vemurafenib is available as bottles of 120 tablets.

15.33 Storage

Store at room temperature 20ºC to 25 ºC (68 ºF to 77 ºF); excursions permitted between 15ºC and 30ºC (59ºF and 86ºF).

15.34 Administration

Vemurafenib is taken orally twice daily with or without food. Doses should be administered in the morning and evening, approximately 12 hours apart. If a dose of vemurafenib is missed (ie 3 hours or more late for vemurafenib), do not make up that dose; resume dosing with the next scheduled dose. If vomiting occurs after a dose is taken, do not take an additional dose; continue with the next scheduled dose. Swallow tablets whole with a glass of water, do not crush or chew.

15.35 Pharmacokinetic information

Absorption: Bioavailability not determined.

Distribution: Vd ~ 106L. Plasma protein binding is > 99% to albumin and α1-acid glycoprotein.
Metabolism: Following oral administration of 960 mg of vemurafenib, mean data showed that vemurafenib and its metabolites represented 95% and 5% of the components in plasma over 48 hours respectively.
Half-life, elimination: 57 hours (range: 30 to 120 hours)

Time to peak: ~ 3 hours

Excretion: Feces ~94%, urine ~1%

15.36 Potential drug interactions

Vemurafenib is a substrate of CYP3A4 based on in vitro data; therefore, coadministration of strong CYP3A4 inhibitors or inducers may alter vemurafenib concentrations. Avoid the co-administration of vemurafenib with strong CYP3A4 inhibitors (i.e. ketoconazole, itraconazole, clarithromycin, atazanavir, nefazodone, saquinavir, telithromycin, ritonavir, indinavir, nelfinavir, voriconazole) or strong inducers (i.e. phenytoin, carbamazepine, rifampin, rifabutin, rifapentine, phenobarbital) and replace these drugs with alternatives when possible.

Vemurafenib may increase concentrations of CYP1A2 substrates. Coadministration of tizanidine, a sensitive CYP1A2 substrate, increased tizanidine systemic exposure by 4.7-fold. Avoid the concomitant use of vemurafenib and CYP1A substrates with a narrow therapeutic window. If co-administration cannot be avoided, monitor closely for toxicities and consider a dose reduction of CYP1A2 substrates.

Coadministration of vemurafenib with digoxin, a sensitive P-glycoprotein substrate, increased digoxin systemic exposure by 1.8-fold. Avoid the concurrent use of P-glycoprotein substrates known to have narrow therapeutic indices. If use of these medications is unavoidable, consider a dose reduction of the P-glycoprotein substrate with narrow therapeutic indices.

15.37 Known potential adverse events

Coadministration of vemurafenib with digoxin, a sensitive P-glycoprotein substrate, increased digoxin

Greater than 10%:
Cardiovascular: Peripheral edema
Central nervous system: Fatigue, headache
Dermatologic: Skin rash, skin photosensitivity, alopecia, pruritus, hyperkeratosis, maculopapular rash, xeroderma, sunburn, erythema, papular rash
Gastrointestinal: Nausea, diarrhea, vomiting, decreased appetite, constipation, dysgeusia
Hematologic/oncologic: Cutaneous papilloma, squamous cell carcinoma of the skin
Hepatic: Increased gamma-glutamyl transferase
Neuromuscular & skeletal: Arthralgia, myalgia, limb pain, back pain, musculo-skeletal pain, weakness
Renal: Increased serum creatinine
Respiratory: Cough
Miscellaneous: Fever

1 to 10%:
Cardiovascular: Atrial fibrillation, hypotension, prolonged QT interval on ECG, retinal vein occlusion, vasculitis
Central nervous system: Cranial nerve palsy, dizziness, peripheral neuropathy
Dermatologic: Erythema nodosum, folliculitis, palmar-plantar erythrodysesthesia, Stevens Johnson syndrome, toxic epidermal necrolysis
Endocrine & metabolic: Weight loss
Hematologic & oncologic: Basal cell carcinoma, malignant melanoma, squamous cell carcinoma
Hepatic: Increased serum alkaline phosphatase, increased serum ALT, increased serum AST, increased serum bilirubin

Hypersensitivity: Anaphylaxis, hypersensitivity
Neuromuscular & skeletal: Arthritis
Ophthalmic: Blurred vision, iritis, photophobia, uveitis

Less than 1% (limited to important or life-threatening): Acute interstitial nephritis, acute renal failure, acute tubular necrosis, chronic myelomonocytic leukemia with NRAS mutation (progression of preexisting condition), drug reaction with eosinophilia and systemic symptoms (DRESS syndrome), febrile neutropenia, hepatic failure, neutropenia, pancreatitis, panniculitis, recall skin sensitization

15.38 Drug procurement

Supplied by Genentech to each site.

Each participating treating location will be responsible for monitoring the supply and will use the Drug Order Request Form to order additional supplies as needed.

Outdated or remaining drug is to be destroyed on-site per procedures in place at each institution.

15.39 Nursing Guidelines

15.391 Instruct patients to take doses by mouth, approximately 12 hours apart in am and pm. Can be taken regardless of food intake. If patient misses a dose by more than 3 hours, do not make up that dose. Swallow whole, do not crush or chew.

15.392 Instruct patient to report any rash, which is common. Patients that have rash and/or blisters accompanied by fever and/or oral or anogenital lesions, should be seen emergently to rule out Steven's Johnson Syndrome.

15.393 Diarrhea and nausea are common. Treat symptomatically and monitor for effectiveness

15.394 Photosensitivity is common and can be quite severe with blistering burns, with little sun exposure. Instruct patients to photo protect with sunscreen, protective clothing, and avoidance of intense sun exposure

15.395 Warn patients of possibility of secondary skin cancers. Instruct patients to report any new lesions to study team immediately

15.396 Warn patient of possible alopecia

15.397 Monitor LFTs, especially in combination therapy.

15.398 Agent may cause arthralgias. Treat symptomatically and monitor for effectiveness

15.399 a Hand-foot syndrome has been seen. Instruct patients to report any pain and/or redness, thickening of skin and/or skin peeling of hands or feet to the study team.

15.399b Agent causes QTc prolongation. Instruct patients to discuss any new medications with the study team, prior to starting such agents

15.399c Agent can cause peripheral edema.

15.399d Rarely ocular toxicity can occur including retinitis, uveitis, and retinal vain occlusion. Instruct patients to report any vision changes and/or eye pain to the study team.

15.4 Tiragolumab (RO7092284, MTIG7192A)

15.41 Background

Tiragolumab is a fully human monoclonal antibody that binds to the inhibitory immunoreceptor TIGIT (T-cell Immunoreceptor with Immunoglobulin and Immunoreceptor Tyrosine-Based Inhibition Motif domains). Therapeutic blockade of TIGIT by tiragolumab represents an attractive strategy for cancer therapy and is expected to enhance the magnitude and quality of the tumor specific T-cell responses and to enhance NK cell-mediated anti-tumor immunity, which may result in improved meaningful anti-tumor activity when tiragolumab is used as a single agent or in combination with other cancer immunotherapies.

15.42 Formulation

Tiragolumab is provided in two configurations:

- Tiragolumab (F01-01): Provided in single-dose 20 mL glass vials containing 20 mL of tiragolumab drug product and buffered in histidine solution containing polysorbate 20, sucrose, and water for injection. The approximate concentration of tiragolumab antibody in the vials is 20 mg/mL. A matching placebo is available in single-dose glass vials containing 20 mL of histidine solution containing polysorbate 20, sucrose, and water for injection.
- Tiragolumab (F03-01): Provided in single-dose 15 mL glass vials containing 10 mL of tiragolumab drug product buffered in histidine solution containing polysorbate 20, sucrose, L-methionine, and water for injection. The approximate concentration of tiragolumab antibody in the vials is approximately 60 mg/mL. A matching placebo is available in single-dose 15 mL glass vials containing 10 mL of histidine solution containing polysorbate 20, sucrose, L-methionine, and water for injection.

The recommended storage conditions for tiragolumab and matching placebo is 2°C – 8°C, protected from light. Tiragolumab must not be frozen.

15.43 Preparation and storage

Tiragolumab may be administered undiluted or after dilution in 0.9% NaCL to a concentration between 0.2 mg/mL – 12 mg/mL. The dose solution should be used immediately. If not used immediately, the total storage time of the dose solution prior to administration should not exceed 24 hours to limit the risk of microbial growth in case of accidental contamination. The recommended storage condition for the dose solution is 2°C – 8°C, but dose solutions may be stored at room temperature for up to a maximum of 4 hours.

The compatibility with diluents other than 0.9% NaCl is unknown.

15.44 Administration

Tiragolumab is delivered through an IV administration set with a 0.2 micron inline filter over 60 minutes. Tiragolumab must not be infused into the same line or cannula concomitantly with other drug infusions, including parenteral nutrition. Infusions of blood products and any electrolyte supplementation must not occur simultaneously with infusions of tiragolumab.

15.45 Pharmacokinetic information

Elimination half-life: Elimination half-life is approximately 15 days

15.46 Potential drug interactions

No specific drug interaction studies have been performed with tiragolumab

15.47 Known potential adverse events

Infusion-related reactions (IRRs) are well recognized side effects of monoclonal antibody infusions. The incidence and severity of IRRs vary according to the target antigen. Based on clinical trial experience with single-agent tiragolumab, acute IRRs were experienced in 11.9% of patients. Serious IRRs were not reported. Most of IRRs were with Grade 1 and 2 symptoms, such as pyrexia (fever), chills, hypertension, and nausea. Based on clinical trial experience with tiragolumab combined with atezolizumab in a phase 1b and a phase II study, IRRs were experienced in 10.0% and 28.4% of patients, respectively. All IRRs in the combination studies that were related to tiragolumab were Grade 1 and 2, and considered non-serious. Pyrexia was the most commonly reported symptom, but pruritus, anxiety, chills, dyspnea, joint stiffness, rash, temperature intolerance, and wheeze were also reported.

15.48 Drug procurement

Tiragolumab is supplied by Genentech, Inc., to each site for study use.

15.49 Nursing Guidelines:

<Nursing will provide the guidelines separately>

16.0 Statistical Considerations and Methodology

16.1 Study Design and analysis plan

16.11 Neoadjuvant Phase (Pre-surgery)

16.111 Primary Endpoint

16.1111 Arms A and B

The primary objective of the neoadjuvant portion of this trial is to obtain an point and interval estimate of pathologic complete response rate in patients with Stage III BRAFm melanoma after 12 weeks of neoadjuvant vemurafenib/ cobimetinib/ atezolizumab therapy as well as point and interval estimate of pathologic complete response rate in patients with Stage III BRAFwt melanoma after 12 weeks of neoadjuvant atezolizumab therapy.

Pathologic complete response (ypCR) rate defined as the percentage of patients with no residual disease found in the surgical specimen among the patients who began neoadjuvant protocol treatment.

For each patient cohort, estimate of pCR to neoadjuvant treatment rate is calculated as follows: 100% times the number of patients who have no residual disease found on pathologic evaluation of the surgery specimen among the patients who started protocol treatment. An interval estimate will be obtained using the formula for a 90% binomial confidence interval for one sample proportion.

With a sample size of 15 patients per patient cohort, the 90% binomial confidence interval for ypCR rate is given below for a number of possible outcomes.

number of patients with no residual disease found in surgical specimen	ypCR (90% confidence interval for the ypCR rate)
1	6.7% (0.32-27.9%)
4	26.7% (9.7-51.1%)
5	33.3 (14.2-57.7)%
7	46.7% (24.4-70.0)%
10	66.7% (42.2-86.8%)
13	86.7 (63.66-97.6%)
15	100% (81.9-100%)

16.1112 Arm C

The evaluation of Arm C will be identical to Arms A and B for the primary objectives of this pilot study. The primary objective of the <u>neoadjuvant</u> treatment phase of Arm C will be to estimate the percentage of patients with Stage III melanoma that achieves a

pathologic complete response (pCR) after 12 weeks of therapy with atezolizumab and tiragolumab. The primary objective of the adjuvant treatment phase of Arm C will be to assess recurrence-free survival (RFS) in patients with Stage III melanoma after neoadjuvant atezolizumab and tiragolumab, surgery, and adjuvant atezolizumab. Secondary objectives include estimating the pCR rate and RFS amongst patients with BRAF-mutated and BRAF-wild-type melanoma and determining the frequency of adverse events among patients with Stage III melanoma treated with neoadjuvant atezolizumab/tiragolumab followed by surgery and adjuvant atezolizumab.

Arm C will enroll 32 patients to initiate neoadjuvant treatment. All patients initiating neoadjuvant treatment will be used to estimate pCR. Similar to what is defined in the protocol and assuming a sample size of 32, the 90% binomial confidence interval for the pathologic complete response rate (pCR) is given below for a number of possible outcomes:

number of patients with no residual disease found in surgical specimen	pCR (90% confidence interval for the pCR rate)
1	3.1% (0.2 – 14%)
2	6.3% (1.1 – 18.4%)
5	15.6% (6.4 – 30.1%)
6	18.8% (8.5 – 33.7%)
7	9.4% (10.7 – 37.2%)
10	31.3 (18 – 47.2%)
11	34.4% (20.6 – 50.4%)
12	37.5% (23.3 – 53.6%)
15	48.9% (31.5 – 62.7%)
16	50% (34.1 – 65.5%)
17	53.1% (37.3 – 68.5%)
20	62.5% (46.4 – 76.7%)
21	65.6% (49.6 – 79.4%)
22	68.8% (52.8 – 82%)
26	81.3% (66.3 – 91.5%)
27	84.4% (70 – 93.6%)
28	87.5% (73.6 – 95.6%)
31	96.9% (86 – 99.8%)
32	100% (91.1 – 100%)

16.112 Secondary Endpoints

Secondary Endpoints include safety profile of these regimens and difference in the uptake on PET/CT scan taken at the completion of neoadjuvant treatment and the uptake on PET/CT scan taken prior to the start of neoadjuvant treatment.

For each patient cohort, the percent change in the uptake on PET/CT scan taken at the completion of neoadjuvant treatment from that on PET/CT scan taken prior to the start of neoadjuvant treatment will be determined. Also, the appearance of new sites of uptake in the post

neoadjuvant treatment scan and lack of uptake in the areas where uptake was seen in the pre-treatment scan will be noted.

For each patient cohort, the frequency and severity of toxicities will be documented using the CTCAE criteria and tabulated for the neoadjuvant phase and post-operative visit separately.

A secondary endpoint is determining the pCR rate in BRAF-mutated and BRAF-wild-type cohorts. Assuming a 60/40 mix of patients, we anticipate enrolling 19 BRAF-wild-type and 13 BRAF-mutated patients. The rate of pCR will be estimated within each of these groups by providing a point estimate with a 90% binomial confidence interval. See the tables below.

BRAF-wild-type (n=19)

number of patients with no residual disease found in surgical specimen	pCR (90% confidence interval for the pCR rate)
1	5.3% (0.3 – 22.6%)
3	15.8% (4.5 – 35.9%)
6	31.6% (14.8 – 53%)
7	36.8% (18.8 – 58.2%)
8	42.1% (23 – 63.2%)
11	57.9% (36.8 – 77%)
12	33.3% (41.8 – 81.3%)
13	40.6% (47 – 85.3%)
17	89.5% (70.4 – 98.1%)
18	94.7% (77.4 – 99.7%)
19	100% (85.4 – 100%)

BRAF-mutated (n=13)

number of patients with no residual disease found in surgical specimen	pCR (90% confidence interval for the pCR rate)
1	7.7% (0.4 – 31.6%)
2	15.4% (2.8 – 41%)
5	38.5% (16.6 – 64.5%)
6	46.2% (22.4 – 71.3%)
7	53.8% (28.7 – 77.6%)
10	76.9% (50.5 – 93.4%)
11	84.6% (60 – 97.2%)
12	92.3% (68.4 – 99.6%)
13	100% (79.4 – 100%)

16.12 Adjuvant Phase (Post-surgery)

The primary endpoint is the median recurrence-free (RFS) rate. Recurrence-free survival is defined as the time from surgery to radiographic or histologic evidence of local, regional, or distant recurrence of melanoma or death due to any cause

All patients who began adjuvant treatment will be included in the analysis of RFS. Those without evidence of disease recurrence will be censored at the time of their last disease evaluation. For each regimen, the distribution of recurrence-free survival times [denoted as RFS(t)] will be estimated using the Kaplan-Meier method. A 90% confidence interval for V(t) = log [-log (RFS(t)] will be constructed and then the inverse transformation exp[-exp v(t)] will be used to obtain a confidence for RFS(t). We will estimate RFS for all patients and separately for BRAF-mutated and BRAF-wild-type patients.

16.13 Study Duration

16.131 Arms A and B

It is anticipated that the accrual period will be 15-18 months. All patients who complete surgery will be followed until disease progression or a maximum of 5 years after registration. Thus, study duration will be appropriately 5.5 years from opening (enrollment period 15-18 months; neoadjuvant treatment and surgical period after close of enrollment for last patient enrollment being approximately 3 months, then adjuvant treatment and follow-up period after last patient completes surgery being 3 years, and then data analysis and manuscript preparation being approximately 6 months).

16.132 Arm C

We anticipate enrollment of 1 patient per month. An additional 4 patients may be necessary to replace patients refusing to initiate study treatment. That is, maximum enrollment may reach 36 patients (estimated 36 month enrollment period). (At the present rate of enrollment at Mayo Clinic and University of Minnesota, the enrollment period would be approximately 36 months.)

16.2 Correlative endpoints

16.21 Pretreatment sPD-L1, pretreatment tumor PD-L1, and pretreatment intracellular Bim in tumor-related T cells

To gather preliminary data as to whether the RFS of patients with stage III melanoma receiving neoadjuvant vem/cobi/atezo or atezo, followed by surgery and adjuvant atezo differs with respect to iether pretreatment sPD-L1 or pretreatment intracellular Bim in tumor-related T cells, stratified Cox modeling will be conducted with patient cohort as the strata to obtain a point and interval estimate of the hazard of recurrence among those with biomarker levels thought to be delirious relative to the hazard of recurrence among those with biomarker levels thought not to be delirious.

16.22 Other correlative evaluations

Evaluate associations between molecular features of melanomas and the tumor immune microenvironment in responders (pCR) versus non-responders (non-pCR) with mIHC and RNASeq in patients with stage III melanoma after neoadjuvant vem/cobi/atezo or atezo, followed by surgery and adjuvant atezo. Graphs will be constructed to visually compare and contrast the levels of these biomarkers between those who achieve a pCR and those who did not. Binomial confidence intervals for the difference in two independent proportions or t confidence intervals for the difference in two independent means will be used to gain preliminary insights into the difference in these biomarkers among those who achieve a pCR and those who did not.

Changes in the concentration of circulating tumor DNA during neoadjuvant treatment will be correlated with pCR. Graphs will be constructed to visually compare and contrast the level of tumor DNA between those who achieve a pCR and those who do not.

For evaluation of changes in T cell receptor clonality, the percent change in the clonality after post neoadjuvant therapy from pre-neoadjuvant therapy levels will be determined for the intra-tumoral T cells from the primary lesion and for peripheral blood T cells. A waterfall plot of the percent change will be constructed by pCR status. A two sample Wilcoxon rank sum test will be used to assess whether the percent change in intra-tumoral T cells from the primary lesion or in peripheral blood T cells differ among those who have a pCR and those who do not. Also, the proportion of patients who have a change in intra-tumoral T cell clonality and the proportion of patients who have a change in peripheral blood T cell clonality will be determined. For each of these parameters, a 95% binomial confidence interval for the difference in the parameter between those who have a pCR and those who do not will be constructed. Finally, the ratio of the intra-tumoral T cell clonality in the involved lymph node to the intra-tumoral T cell clonality in the uninvolved lymph node will be determined. This ratio will be summarized descriptively using median and inter-quartile range.

For evaluation of changes in peripheral blood immune cell subsets, we will quantitate the percentage of PBMCs comprising each immune cell type at each time point. The change in each immune cell subset frequency from pre- to post-neoadjuvant therapy, post-neoadjuvant therapy to post-surgery, and post-surgery (pre-adjuvant therapy) to post-adjuvant therapy will be calculated. Pre-treatment immune subset frequencies and changes in frequencies that occur during treatment will be correlated with pCR/near pCR. Graphs will be constructed to visually compare and contrast immune cell subset changes between those who achieve a pCR and those who do not.

We plan to perform the same correlative studies for Arm C of MC1776 as for Arms A and B. These include determining the associations between soluble and tissue PD-L1 with RFS, determining the association between intracellular Bim levels on tumor-associated T cells with RFS, evaluating associations between molecular features of melanoma (assessed by RNA-Seq) and the tumor immune microenvironment (assessed by multiplexed immunofluorescence) with RFS, evaluating changes in cell-free DNA over time and correlating these with clinical outcomes, and evaluating T cell receptor diversity and T cell neoantigen responses in peripheral blood and treated lymph nodes. We also plan to study the host microbiome at serial time points in association with the above as well as local and systemic immunity assays and treatment response.

In addition, to further characterize changes in peripheral blood mononuclear cells (PBMCs), we plan to perform CyTOF analysis of peripheral blood mononuclear cells at multiple time points and correlate changes in PBMC subsets with other biomarkers (as noted above) and with RFS.

16.3 Data & Safety Monitoring

16.31 Safety review

The principal investigator(s) and the study statistician will review the study monthly to identify accrual, adverse event, and any endpoint problems that might

be developing. The Mayo Clinic Cancer Center (MCCC) Data Safety Monitoring Board (DSMB) is responsible for reviewing accrual and safety data for this trial at least biannually, based on reports provided by the MCCC Statistical Office.

16.32 Adverse Event Stopping Rules

16.321 Neoadjuvant

Enrollment will be temporarily halted during the neoadjuvant period if 2 or more of the first 6 patients enrolled or 30% or more thereafter develop any Grade 4 adverse event except cytopenias (anemia, thrombocytopenia, leukopenia, neutropenia); if the adverse event is possibly, probably, or definitely related to study treatment. All adverse event data will be reviewed by the study team and if appropriate, a protocol amendment will be drafted for IRB approval.

Enrollment to either study arm will be permanently halted if 2 or more out of first 6 patients or more than 30% of patients enrolled thereafter to that arm progress on therapy and as a result, their disease becomes unresectable.

16.322 Adjuvant

Enrollment will be temporarily halted during the adjuvant period if 2 or more of the first 6 patients receiving adjuvant treatment or 30% or more thereafter develop any Grade 4 adverse event except cytopenias (anemia, thrombocytopenia, leukopenia, neutropenia); if the adverse event is possibly, probably, or definitely related to study treatment. All adverse event data will be reviewed by the study team and if appropriate, a protocol amendment will be drafted for IRB approval.

16.4 Subset Analyses for Minorities

16.41 Study availability

This study will be available to all eligible patients, regardless of gender, race or ethnic origin.

16.42 Statistical analysis by subset

There is no information currently available regarding differential effects of this regimen in subsets defined by race, gender, or ethnicity, and there is no reason to expect such differences to exist. Therefore, although the planned analyses will look for differences in treatment effect based on racial groupings, the sample size is not increased in order to provide additional power for subset analyses.

16.43 Regional population

The geographical region served by MCCC has a population which includes approximately 3% minorities. Expected sizes of racial by gender subsets are shown in the following table:

Accrual Targets			
	Sex/Gender		
**Ethnic Category**	Females	Males	Total
Hispanic or Latino	0	3	3
Not Hispanic or Latino	27	36	63
Ethnic Category: Total of all subjects	**27**	**39**	**66**
**Racial Category**			
American Indian or Alaskan Native	0	0	0
Asian	0	0	0
Black or African American	0	0	0
Native Hawaiian or other Pacific Islander	0	0	0
White	27	39	66
**Racial Category: Total of all subjects**	**27**	**39**	**66**

Ethnic Categories:

Hispanic or Latino – a person of Cuban, Mexican, Puerto Rican, South or Central American, or other Spanish culture or origin, regardless of race. The term "Spanish origin" can also be used in addition to "Hispanic or Latino."

Not Hispanic or Latino

Racial Categories:

American Indian or Alaskan Native – a person having origins in any of the original peoples of North, Central, or South America, and who maintains tribal affiliations or community attachment.

Asian – a person having origins in any of the original peoples of the Far East, Southeast Asia, or the Indian subcontinent including, for example, Cambodia, China, India, Japan, Korea, Malaysia, Pakistan, the Philippine Islands, Thailand, and Vietnam. (Note: Individuals from the Philippine Islands have been recorded as Pacific Islanders in previous data collection strategies.)

Black or African American – a person having origins in any of the black racial groups of Africa. Terms such as "Haitian" or "Negro" can be used in addition to "Black or African American."

Native Hawaiian or other Pacific Islander – a person having origins in any of the original peoples of Hawaii, Guam, Samoa, or other Pacific Islands.

White – a person having origins in any of the original peoples of Europe, the Middle East, or North Africa.

17.0 Pathology Considerations/Tissue Biospecimens

17.1 Summary Table of Research Tissue Specimens to be Collected for this Protocol

Correlative Study	Mandatory or Optional	Type of Tissue to Collect	Block, Slides, Core, etc. (# of each to submit)	After consenting to pre-registration phase	At the time of surgery	At the time of progression/ recurrence*	Prepare at site? (Yes or No)	Temperature/ Conditions for Storage /Shipping
Multiplex and PD-L1 IHC	Mandatory[1]	Formalin Fixed Paraffin	Core needle biopsy (6 slides)	X	---		Yes	Ambient
	Mandatory	Formalin Fixed Paraffin	Surgical specimen (12 slides)	---	X[2]		Yes	Ambient
	Optional	Formalin Fixed Paraffin	Core needle biopsy or surgical specimen (6 slides)	--	--	X	Yes	Ambient
T Cell Receptor Sequencing	Mandatory	Formalin Fixed Paraffin	Surgical specimens** (5 scrolls/specimen)		X		Yes	Ambient
RNA-Seq	Mandatory	Frozen†	Excess surgical tissue		X[2]			Frozen
	Mandatory	Formalin Fixed Paraffin	Core needle biopsy (6 slides)	X			Yes	Ambient
	Mandatory	Formalin Fixed Paraffin	Surgical specimen (6 slides)		X[2]		Yes	Ambient

After pre-reg sample, patient may refuse subsequent samples and eligibility is not affected.

*Neoadjuvant or adjuvant study period

1. Archival specimen acceptable
2. If surgery will not be performed, then a research biopsy should be obtained including FFPE and frozen cores (2-3 cores each type)

**For T cell receptor sequencing studies, surgical specimens should include both tumor/involved lymph node AND uninvolved lymph node. The 5 tissue scrolls should be 5 microns thick each.

†NOTE: Frozen tissue collection is described in Section 14.0, due to the separate collection/shipping process through BAP.

17.2 Paraffin Embedded Tissue

Submit six formalin fixed paraffin-embedded (FFPE) tumor slides. Specimens will be shipped to Dr. Block's laboratory: Attn: Courtney Erskine, Guggenheim 3-23, Mayo Clinic, 200 First St SW, Rochester MN 55905.

For patients who submit tissue for pre-registration determination of BRAF status, and do not subsequently enroll in the study, research tissue will be returned or destroyed.

17.3 Central Pathology Review

Central Pathology Review of H&E from surgical specimens will be performed at Mayo Clinic. Specimens will be reviewed for the areas involved by viable tumor, melanosis/necrosis, and fibrosis/inflammation.

17.4 Background and methodology

17.41 PD-L1 IHC

Validated in lung cancer as a biomarker of response, PD-L1 expression assessed by IHC in tumor tissue has been reported to correlate with treatment response in a recently published melanoma adjuvant therapy studies (Weber 2017). However, validation of cut-points and the best approach to evaluate heterogeneous staining remains to be determined (Scognamiglio 2016). We will test biopsy samples from the time of diagnosis and samples tested at the time of operation to evaluate the association, if any, to neoadjuvant therapy response and other endpoints. FFPE sections will be stained using a commercially available FDA-approved kit and percent mem brane staining assessed qualitatively.

Immunohistochemistry for PD-L1 will be performed using the FDA-approved kit VENTANA PD-L1 (SP263) Assay which is a qualitative immunohistochemical assay using rabbit monoclonal anti-PD-L1 clone SP263 intended for use in the assessment of the PD-L1 protein in formalin-fixed, paraffin-embedded (FFPE) tissue.

PD-L1 status will be determined by the percentage of tumor cells with any membrane staining above background or by the percentage of tumor-associated immune cells with staining (IC+) at any intensity above background. The percent of tumor area occupied by any tumor-associated immune cells (Immune Cells Present, ICP) is used to determine IC+, which is the percent area of ICP exhibiting PD-L1 positive immune cell staining. Staining will be assessed semi-quantitatively based on percent membrane staining.

17.42 Multiplex IF

To understand the complex interactions that take place in the melanoma tumor microenvironment (TME), we need to not only identify and quantify the key cellular and molecular mediators of immunity and immune escape, but also elucidate how the different entities interact with one another. To this end, we have developed a multiplex immunohistochemistry platform that can a) quantify the immune infiltrate of metastatic lesions, b) calculates the localization and frequency with which each immune cell type contacts other immune cells and tumor cell, c) reveal the proliferation versus death of tumor cell. We are interested primarily in infiltration by 1) lymphocytes: CD8+ T cells, CD4+ T cells, Foxp3+ regulatory T-cells, CD56 and CD16 NK cells, B220+ B-cells, 2) activated or exhausted status of these lymphocytes as revealed by expression of CD28 co-T-cell-receptor, ICOS T-cell activation marker, PD1, PD-L1, CTLA4, inhibitory molecules, 3) inflammation as shown by presence of

CD33+CD15+DR- as well as CD66b neutrophils and CD33+CD14+DR- monocytes, 4) cytoxicity and tumor cell death as measured by TUNEL, perforin, and GrzB, 5) expression of major histocompatibility antigens that report presence of antigen presenting cells and visibility of tumor cells to immune cells, namely HLA class 1 and HLA class 2 . We will perform multiplex IF on both pre-treatment biopsies and post-neoadjuvant surgical specimens. In this way, we will develop a preliminary set of baseline immune cells and interactions and changes in immune cells/interactions that correlate strongly with clinical responses to neoadjuvant therapy.

17.43 RNA-Seq

Recognizing that markers of clinical response or lack of response to targeted and immunotherapies is of great current interest and clinical relevance we propose further analysis with whole-transcriptome sequencing (RNA-Seq). A greater understanding of the mechanisms underpinning clinical response may lead to relevant biomarker discovery and provide a scientific rationale for future combinational therapies to improve response and clinical outcomes. We propose extraction of total RNA from pre- and post-treatment samples followed by mRNA isolation, cDNA and library prep using the established Illumina platforms, followed by qPCR and sequencing on the HiSeq platform. Bioinformatics with statistical tools such as edgeR will be performed for normalization, quantitative analysis and differential expression (Robinson 2010). We are particularly interested in associations of the combined visual and spatial data from multiplex IHC with genomic data and postulate that this will provide a rich discovery tool for understanding the clinical responses to neoadjuvant therapy.

17.44 T cell receptor sequencing

T cells undergo somatic gene rearrangement of the T cell receptor Valpha and Vbeta regions during differentiation. In this way, each T cell clone has a uniquely rearranged T cell receptor; this allows different T cells to recognize distinct antigens. By performing sequencing of individual T cell receptor Valpha and/or Vbeta regions, the diversity of T cell clones in a given tissue, as well as the degree of clonal expansion of individual clones, can be determined. To assess for the diversity and clonality of T cell receptors in tumor/involved lymph nodes and uninvolved lymph nodes, 5 paraffin tissue scrolls (5 microns each) will be sent to Adaptive Biotechnologies for T cell receptor sequencing

Adaptive Biotechnologies
1551 Eastlake Ave E, Ste 200
Seattle, WA 98102

In addition, by stimulating PBMCs with candidate neoantigen peptides, we can identify TCR sequences from neoantigen-specific T cells and track the neoantigen-specific T cells at each time point in both blood and tissue. First, somatic mutations will be identified by comparing whole exome sequencing of tumor with whole genome sequencing of blood. Patient HLA class I and class II alleles will be identified from sequencing results as well. Peptides spanning each somatic mutation will be assessed in silico for predicted binding to patient-specific HLA alleles. Peptides that a) contain at least one somatic mutation, and b) are predicted to bind to one of the patient's HLA alleles will be considered candidate neoantigens. Candidate neoantigen peptides will be used to stimulate PBMCs, and TCR sequences from peptide-activated PBMCs will be considered neoantigen-specific TCRs.

18.0 Records and Data Collection Procedures

18.1 Submission Timetable

Data submission instructions for this study can be found in the Case Report Form packet.

18.2 Event monitoring/Survival and Disease Status Follow-up

See Section 4.0 and Data Submission Table in the Case Report Form packet for the event monitoring schedule.

18.3 CRF completion

This study will use Medidata Rave® for remote data capture (rdc) of all study data.

18.4 Site responsibilities

Each site will be responsible for insuring that all materials contain the patient's initials, MCCC registration number, and MCCC protocol number. Patient's name must be removed.

18.5 Supporting documentation

This study requires pathology report to confirm Stage III melanoma and BRAFV600 results at baseline and supporting documentation (pathology report, imaging, or clinical note) for evidence of recurrence/progression after study therapy.

To submit these materials, they can be uploaded into the Supporting Documentation form in Medidata Rave®. These reports should be submitted within 14 days of registration for baseline and within 14 days of discovery for recurrence/progression on study.

18.6 Incomplete materials

Any data entered into a form will result in that form being marked as "received." However, missing data will be flagged by edit checks in the database.

18.7 Overdue lists

A list of overdue materials is automatically available to each site at any time. A list of overdue materials and forms for study patients will be generated monthly. The listings will be sorted by location and will include the patient study registration number. The appropriate co-sponsor/participant will be responsible to obtain the overdue material.

18.8 Corrections forms

If a correction is necessary the QAS will query the site. The query will be sent to the appropriate site to make the correction in the database and respond back to the QAS.

19.0 Budget

19.1 Costs charged to patient: routine clinical care

19.2 Tests to be research funded: research blood draws, research biopsies, BRAF testing, infusion of study drug; and correlative research studies

19.3 Other budget concerns: Study drugs to be provided by Genentech, additional testing required by Genentech for ophthalmic exams and liver function testing, to be paid by Genentech

20.0 References

Ascierto PA, et al. Cobimetinib combined with vemurafenib in advanced BRAFV600-mutant melanoma (coBRIM): updated efficacy results from a randomised, double-blind, phase 3 trial. Lancet Oncol 2016; 17(9):1248-1260.

Adashek ML, Feldman M. Cytokine release syndrome resulting from anti–programmed death-1 antibody: Raising awareness among community oncologists. J Oncol Pract 2019;15:502–4.

Balar AV, et al. Atezolizumab as first-line treatment in cisplatin-ineligible patients with locally advanced and metastatic urothelial carcinoma: a single-arm, multicentre, phase 2 trial. Lancet. 2017;389(10064):67-76.

Balch CM, et al. Multivariate analysis of prognostic factors among 2,313 patients with stage III melanoma: comparison of nodal micrometastases versus macrometastases. J Clin Oncol. 2010;28(14):2452-9.

Blank CU, et al. Open-label, multicentre safety study of vemurafenib in 3219 patients with BRAF^{V600} mutation-positive metastatic melanoma: 2-year follow-up data and long-term responders' analysis. Eur J Cancer. 2017 Jul;79:176-184. doi: 10.1016/j.ejca.2017.04.007

Blank, C.U., et al., *Neoadjuvant versus adjuvant ipilimumab plus nivolumab in macroscopic stage III melanoma.* Nat Med, 2018. **24**(11): p. 1655-1661

Blessin, N.C., et al., *Patterns of TIGIT Expression in Lymphatic Tissue, Inflammation, and Cancer.* Dis Markers, 2019. **2019**: p. 5160565.

Boni A, Cogdill AP, Dang P, et al. Selective BRAFV600E inhibition enhances T-cell recognition of melanoma without affecting lymphocytes function. Cancer Res 2010;70:5213-9.

Capirci C, et al. Prognostic Value of Pathologic Complete Response After Neoadjuvant Therapy in Locally Advanced Rectal Cancer: Long-Term Analysis of 566 ypCR Patients. International Journal of Radiation Oncology*Biology*Physics; 72(1): 2008; 99-107.

Caporaso JG, Kuczynski J, Stombaugh J, et al. QIIME allows analysis of high-throughput community sequencing data. Nat Methods. 2010;7(5):335-336

Caporaso JG, Lauber CL, Walters WA, et al. Ultra-high-throughput microbial community analysis on the Illumina HiSeq and MiSeq platforms. ISME J. 2012;6(8):1621-1624.

Chang LH, Pan SL, Lai CY, et al. Activated PAR-2 regulates pancreatic cancer progression through ILK/HIF-α-induced TGF-α expression and MEK/VEGF-A-mediated angiogenesis. Am J Pathol 2013;183(2):566–75

Ciuffreda L, Del Bufalo D, Desideri M, et al. Growth-inhibitory and antiangiogenic activity of the MEK inhibitor PD0325901 in malignant melanoma with or without BRAF mutations. Neoplasia 2009;11:720–31.

Clinical Trials.gov. Melanoma studies. https://clinicaltrials.gov/ct2/results?cond=Melanoma&term=neoadjuvant+therapy&cntry1=NA%3AUS&state1= accessed 9/20/17

Cortazar P, Geyer CE. Pathological complete response in neoadjuvant treatment of breast cancer. Annals Surg Oncol 2015 May 1;22(5):1441-6.].

Davies H, et al. Mutations of the BRAF gene in human cancer. Nature 2002;417:949−54.

Donohoe CL, Reynolds JV. Neoadjuvant treatment of locally advanced esophageal and junctional cancer: The evidence-base, current key questions and clinical trials. J Thorac Dis. 2017 Jul;9(Suppl 8):S697-S704. doi: 10.21037/jtd.2017.03.159.

Dronca RS, et al. T cell Bim levels reflect responses to anti-PD-1 cancer therapy H. JCI Insight. 2016 May 5;1(6). pii: e86014. PMID: 27182556

Dronca RS, et al. Bim and soluble PD-L1 (sPD-L1) as predictive biomarkers of response to anti-PD-1 therapy in patients with melanoma and lung carcinoma. DOI: 10.1200/JCO.2017.35.15_suppl.11534 Journal of Clinical Oncology 35, no. 15_suppl (May 2017) 11534-11534

Ebert PJ, Cheung J, Yang Y, et al. MAP Kinase Inhibition Promotes T Cell and Anti-tumor Activity in Combination with PD-L1 Checkpoint Blockade. Immunity 2016;44(3):609–21.

Eggermont AM et al, Adjuvant ipilimumab versus placebo after complete resection of high-risk stage III melanoma (EORTC 18071): a randomised, double-blind, phase 3 trial. Lancet Oncol. 2015 May;16(5):522-30. doi: 10.1016/S1470-2045(15)70122-1].

Faries MB. The impact on morbidity and length of stay of early versus delayed complete lymphadenectomy in melanoma: results of the Multicenter Selective Lymphadenectomy Trial (I) Ann Surg Oncol 2010; 17:3324–3329].

Fehrenbacher L, et al. Atezolizumab versus docetaxel for patients with previously treated non-small-cell lung cancer (POPLAR): a multicentre, open-label, phase 2 randomised controlled trial. Lancet. 2016;387(10030):1837-46.].

Frederick DT, Piris A, Cogdill AP, et al. BRAF inhibition is associated with enhanced melanoma antigen expression and a more favorable tumor microenvironment in patients with metastatic melanoma. Clin Cancer Res 2013;19:1225-31.

Fridericia LS (1920) Die Systolendauer im Elektrokardiogramm bei normalen Menschen und bei Herzkranken. [The duration of systole in the electrocardiogram of normal subjects and of patients with heart disease.] Acta Medica Scandinavica 53:469–486.

Frigola X, et al. Identification of a soluble form of B7-H1 that retains immunosuppressive activity and is associated with aggressive renal cell carcinoma. Clin Cancer Res. 2011 Apr 1;17(7):1915-23.

Grinshtein N, Bridle B, Wan Y, Bramson JL. Neoadjuvant vaccination provides superior protection against tumor relapse following surgery compared with adjuvant vaccination. Cancer Res. 2009 May 1;69(9):3979-85.

Grob JJ et al. Comparison of dabrafenib and trametinib combination therapy with vemurafenib monotherapy on health-related quality of life in patients with unresectable or metastatic cutaneous BRAF Val600-mutation-positive melanoma (COMBI-v): results of a phase 3, open-label, randomised trial. Lancet Oncol. 2015 Oct;16(13):1389-98. doi: 10.1016/S1470-2045(15)00087-X.

Guy P et al. Vital Signs: Melanoma Incidence and Mortality Trends and Projections — United States, 1982–2030. MMWR Weekly Report 2015; 64:21,591-96

Haass NK et al. The mitogen-activated protein/extracellular signal-regulated kinase kinase inhibitor AZD6244 (ARRY-142886) induces growth arrest in melanoma cells and tumor regression when combined with docetaxel. Clin Cancer Res 2008;14:230-9

Haddad TC, Goetz MP. Landscape of neoadjuvant therapy for breast cancer. Annals Surg Oncol 2015 May 1;22(5):1408-15. AND

Hong DS, Vence L, Falchook G, et al. BRAF(V600) inhibitor GSK2118436 targeted inhibition of mutant BRAF in cancer patients does not impair overall immune competency. Clin Cancer Res 2012;18:2326–35.

Hu-Lieskovan S, Mok S, Homet Moreno B, et al. Improved antitumor activity of immunotherapy with BRAF and MEK inhibitors in BRAF(V600E) melanoma. Sci Transl Med 2015;7:279ra41.

Jeraldo P, Kalari K, Chen X, et al. IM-TORNADO: a tool for comparison of 16S reads from paired-end libraries. PloSOne. 2014;9(12):e114804

Johnston, R.J., et al., *The immunoreceptor TIGIT regulates antitumor and antiviral CD8(+) T cell effector function.* Cancer Cell, 2014. **26**(6): p. 923-937.

Jumpstart Consortium Human Microbiome Project Data Generation Working Group. Evaluation of 16S rDNA-based community profiling for human microbiome research. PloSOne. 2012;7(6):e39315.

Kakavand H, Wilmott JS, Menzies AM, et al. PD-L1 Expression and Tumor-Infiltrating Lymphocytes Define Different Subsets of MAPK Inhibitor-Treated Melanoma Patients. Clin Cancer Res 2015;21:3140-8.

Larkin J, et al. Combined vemurafenib and cobimetinib in BRAF-mutated melanoma. N Engl J Med. 2014;371(20):1867-76.

La Rosée P. Treatment of hemophagocytic lymphohistiocytosis in adults. Hematology Am Soc Hematol Educ Program 2015;1:190–6.

La Rosée P, Horne A, Hines M, et al. Recommendations for the management of hemophagocytic lymphohistiocytosis in adults. Blood 2019;133:2465–77.

Lee DW, Santomasso BD, Locke FL, et al. ASTCT consensus grading for cytokine release syndrome and neurologic toxicity associated with immune effector cells. Biol Blood Marrow Transplant 2019;25:625–38.

Liu I, Mayes PA, Eastman S, et al. The BRAF and MEK inhibitors dabrafenib and trametinib: effects on immune function and ombination with immunomodulatory antibodies targeting PD-1, PD-L1, and CTLA-4. Clin Cancer Res 2015;21:1639-51.

Liu J. Improved efficacy of neoadjuvant compared to adjuvant immunotherapy to eradicate metastatic disease. Cancer Discover 2016; 6:1382-1399.

Loi S, Dushyanthen S, Beavis PA, et al. RAS/MAPK activation is associated with reduced tumor-infiltrating lymphocytes in triple-negative breast cancer: therapeutic cooperation between MEK and PD-1/PD-L1 immune checkpoint inhibitors. Clin Cancer Res 2016;22:1499–509.

Manieri, N.A., E.Y. Chiang, and J.L. Grogan, *TIGIT: A Key Inhibitor of the Cancer Immunity Cycle.*

Trends Immunol, 2017. **38**(1): p. 20-28.

McClain KL, Eckstein O. Clinical features and diagnosis of hemophagocytic lymphohistiocytosis. Up to Date [resource on the Internet]. 2014 [updated 29 October 2018; cited: 17 May 2019]. Available from: https://www.uptodate.com/contents/clinical-features-and-diagnosis-of-hemophagocytic-lymphohistiocytosis.

Menzies AM, et al. Preliminary results from the international neoadjuvant melanoma consortium (INMC). J Clin Oncol 2017; 35(15_suppl) 9581

Merad M, Martin JC. Pathological inflammation in patients with COVID-19: a key role for monocytes and macrophages. Nat Rev Immunol 2020;20:355–62.

Mohan S, Vander Broek R, Shah S, Eytan DF, Pierce ML, Carlson SG, Coupar JF, Zhang J, Cheng H, Chen Z, Van Waes C. MEK Inhibitor PD-0325901 Overcomes Resistance to PI3K/mTOR Inhibitor PF-5212384 and Potentiates Antitumor Effects in Human Head and Neck Squamous Cell Carcinoma. Clin Cancer Res 2015;21:3946-56.

Morton D et al. Final trial report of sentinel-node biopsy versus nodal observation in melanoma. New Engl J Med 2014; 370:599-609

Phan VT, Wu X, Cheng JH, et al. Oncogenic RAS pathway activation promotes resistance to anti-VEGF therapy through G-CSF-induced neutrophil recruitment. Proc Natl Acad Sci USA 2013;110:6079-84.

Prieto PA, et al. Novel neoadjuvant targeted therapy trial with dabrafenib + trametinib yields high response rates and improved relapase free survival over standard of care (SOC) therapy in patients with high-risk resectable BRAF-mutant melanoma (abstract). Ann Surg Oncol 2017; 24(S1):S7

Ravelli A, Minoia F, Davi S, et al. 2016 classification criteria for macrophage activation syndrome complicating systemic juvenile idiopathic arthritis: a European League Against Rheumatism/American College of Rheumatology/Paediatric Rheumatology International Trials Organisation Collaborative Initiative. Ann Rheum Dis 2016;75:481–9.

Riegler LL, Jones GP, Lee DW. Current approaches in the grading and management of cytokine release syndrome after chimeric antigen receptor T-cell therapy. Ther Clin Risk Manag 2019;15:323–35.

Robinson MD, et al. edgeR; A Bioconductor conductor package for differential expression analysis of digital gene expression data. Bioinforma Oxf Engl 2010; 26:139-40

Rodriguez-Abreu D., et al., Primary analysis of a randomized, double-blind, phase II study of the anti-TIGIT antibody tiragolumab (tira) plus atezolizumab (atezo) versus placebo plus atezo as first-line (1L) treatment in patients with PD-L1-selected NSCLC (CITYSCAPE). J Clin Oncol, 2020. **38S :** abstr 9503.

Romano E, et al. Site and Timing of First Relapse in Stage III Melanoma Patients: Implications for Follow-Up Guidelines. J Clin Oncol 2010; 28;18:3042-7.

Rosenberg JE, et al. Atezolizumab in patients with locally advanced and metastatic urothelial carcinoma who have progressed following treatment with platinum-based chemotherapy: a single-arm, multicentre, phase 2 trial. Lancet 2016;387:1909-20

Rotte, A., J.Y. Jin, and V. Lemaire, Mechanistic overview of immune checkpoints to support the rational design of their combinations in cancer immunotherapy. Annals of Oncology, 2018. **29**(1): p. 71-83.

Rotz SJ, Leino D, Szabo S, et al. Severe cytokine release syndrome in a patient receiving PD-1-directed therapy. Pediatr Blood Cancer 2017;64:e26642.

Schram AM, Berliner N. How I treat hemophagocytic lymphohistiocytosis in the adult patient. Blood 2015;125:2908–14.

Scognamiglio et al Int. J. Mol. Sci. **2016**, 17, 790; doi:10.3390/ijms17050790

Shah DJ, Dronca RS. Latest advances in chemotherapeutic, targeted, and immune approaches in the treatment of metastatic melanoma. Mayo Clin Proc 2014 Apr;89(4):504-19.

Silverberg E, Holleb AI. Cancer statistics 1973. CA: Cancer J Clinicians 1973; 23(1):2-27.

Siegel RL, Miller KD, Jemal A. Cancer Statistics, 2017. CA Cancer J Clin 2017; 67 (1), 7-30.

Sipos M, Geraldo P, Chia N, et al. Robust computational analysis of rRNA hypervariable tag datasets. PLoSOne. 2010;5(12): e15220. doi:10.1371/journal.pone.0015220.

Sivan et al, Science 2015; 27 NOVEMBER 2015 • VOL 350 ISSUE 6264

Solit DB, et al. BRAF mutation predicts sensitivity to MEK inhibition. Nature 2006;(439):358-62.

Song, Y., et al., *Neoadjuvant Versus Adjuvant Immune Checkpoint Blockade in the Treatment of Clinical Stage III Melanoma.* Ann Surg Oncol, 2020. **27**(8): p. 2915-2926.

Sullivan RJ, Hamid O, Gonzalez R, al. Safety and clinical activity of atezolizumab + cobimetinib + vemurafenib in BRAFV600-mutant metastatic melanoma (abstract) . Presented at: Society for Melanoma Research Annual Meeting; Boston, Massachusetts, November 6-9, 2016 and updated in June 2017

Sullivan RJ, et al. Atezolizumab (A) + cobimetinib (C) + vemurafenib (V) in BRAFV600-mutant metastatic melanoma (mel): Updated safety and clinical activity (abstract). J Clin Oncol 2017; 35(15_suppl):3063-3063

Sumimoto H, Imabayashi F, Iwata T, Kawakami Y. The BRAF-MAPK signaling pathway is essential for cancer-immune evasion in human melanoma cells. J Exp Med 2006;203:1651-6.

Svedman et al, Stage-specific survival and recurrence in patients with cutaneous malignant melanoma in Europe – a systematic review of the literature. Clin Epidemiol 2016; 8:109-122

Vansteenkiste J et al. Prospects and progress of atezolizumab in non-small cell lung cancer. Expert Opin Biol Ther. 2017 Jun;17(6):781-789.

Wargo J et al. Neoadjuvant BRAF (dabrafenib) and MEK (trametinib) inhibition for high-risk resectable stage III and IV melanoma (abstract). J Clin Oncol 2015; 33(15_suppl):9091.

Wargo et al DOI: 10.1200/JCO.2017.35.15_suppl.3008 Journal of Clinical Oncology 35, no. 15_suppl (May 2017) 3008-3008.

Weber et al, Adjuvant Nivolumab versus Ipilimumab in Resected Stage III or IV Melanoma, New Engl J Med 2017, 377:1824-35. DOI: 10.1056/NEJMoa1709030

Wilmott JS, Long GV, Howle JR, et al. Selective BRAF inhibitors induce marked T-cell infiltration into human metastatic melanoma. Clin Cancer Res. 2012;18:1386-94.

Zhu Z, Liu W, Gotlieb V. The rapidly evolving therapies for advanced melanoma -- Towards immunotherapy, molecular targeted therapy, and beyond. Crit Rev Oncol Hematol 2016;99:91-9.

Appendix I ECOG Performance Status

ECOG PERFORMANCE STATUS*	
Grade	ECOG
0	Fully active, able to carry on all pre-disease performance without restriction
1	Restricted in physically strenuous activity but ambulatory and able to carry out work of a light or sedentary nature, e.g., light house work, office work
2	Ambulatory and capable of all selfcare but unable to carry out any work activities. Up and about more than 50% of waking hours
3	Capable of only limited selfcare, confined to bed or chair more than 50% of waking hours.
4	Completely disabled. Cannot carry on any selfcare. Totally confined to bed or chair.
5	Dead

*As published in Am. J. Clin. Oncol.:
Oken, M.M., Creech, R.H., Tormey, D.C., Horton, J., Davis, T.E., McFadden, E.T., Carbone, P.P.: Toxicity And Response Criteria Of The Eastern Cooperative Oncology Group. Am J Clin Oncol 5:649-655, 1982.

The ECOG Performance Status is in the public domain therefore available for public use. To duplicate the scale, please cite the reference above and credit the Eastern Cooperative Oncology Group, Robert Comis M.D., Group Chair.

From http://www.ecog.org/general/perf_stat.html

Appendix II MC1776 Patient Medication Diary

Neoadjuvant Treatment Only (Arms A and B only)

Name ___________________________ Study ID Number __________________________

Please complete this diary on a daily basis. Write in the amount of the doses of vemurafenib and/or cobimetinib that you took in the appropriate "Day" box.

NOTE: If you have BRAFwt melanoma, please ignore the vemurafenib lines and complete only the cobimetinib rows.

On the days that you do not take any study drug, please write in "0". If you forget to take your daily dose, please write in "0", but remember to take your prescribed dose at the next regularly scheduled time.

Both vemurafenib and cobimetinib may be taken at the same for the morning dose. Both agents may be taken with or without food. Do not chew or crush tablets.

If you miss a dose or vomit a dose, do not make up the dose. Resume treatment with next scheduled dose.

If you experience any health/medical complaints, please record this

information. Week of: ________________

**Study Drug**		**Day 1**	**Day 2**	**Day 3**	**Day 4**	**Day 5**	**Day 6**	**Day 7**
Vemurafenib	am							
	pm							
Cobimetinib	am							

Week of: ___________________________

**Study Drug**		**Day 8**	**Day 9**	**Day 10**	**Day 11**	**Day 12**	**Day 13**	**Day 14**
Vemurafenib	am							
	pm							
Cobimetinib	am							

Week of: ___________________________

**Study Drug**		**Day 15**	**Day 16**	**Day 17**	**Day 18**	**Day 19**	**Day 20**	**Day 21**
Vemurafenib	am							
	pm							
Cobimetinib	am							

Week of: ___________________________

**Study Drug**		**Day 22**	**Day 23**	**Day 24**	**Day 25**	**Day 26**	**Day 27**	**Day 28**
Vemurafenib	am							
	pm							

Cobimetinib	am							

Week of: ___________________________*

Study Drug		Day 29	Day 30	Day 31	Day 32	Day 33	Day 34	Day 35
Vemurafenib	am							
	pm							
Cobimetinib	am							

*NOTE: Extra days provided for patient convenience.

Patient signature: ____ Date: _______________

Health or medical complaints during this time:

My next scheduled visit is: __________________________________

If you have any questions, please call: ________________________

Study Coordinator Use Only

Number of pills returned ______ Number of vials returned: _______

Discrepancy Yes____/No____ Verified by __________________ Date________________

Appendix III General Guidelines for Concomitant Therapy and Additional Restrictions*

*NOTE: Protocol and investigator discretion are key.

Concomitant therapy includes any medication (e.g., prescription drugs, over-the-counter drugs, vaccines, herbal or homeopathic remedies, nutritional supplements) used by a patient in addition to protocol-mandated study treatment from 7 days prior to signing of the Informed Consent Form through 30 days after the last dose of study treatment.

Permitted Therapy

Patients who use oral contraceptives, hormone-replacement therapy, prophylactic anticoagulation therapy (such as low-molecular-weight heparin at a stable dose level), or other allowed maintenance therapy should continue their use. Male and female patients of reproductive potential should use highly effective means of contraception (see Section 9.7).

Patients are permitted to use the following therapies during the study:

- Oral contraceptives
- Hormone-replacement therapy
- Prophylactic or therapeutic anticoagulation therapy (such as warfarin at a stable dose or low- molecular-weight heparin)
- Inactivated influenza vaccinations
- Megestrol administered as an appetite stimulant
- Inhaled corticosteroids
- Mineralocorticoids
- Low-dose corticosteroids administered for orthostatic hypotension or adrenocortical insufficiency
- Pain medications as indicated, per standard practice

At the discretion of the investigator, anti-emetic medications, antidiarrheal medications, and may be administered prophylactically per standard local practice before the second and subsequent doses of study treatment. Anti-emetic medications, antidiarrheal medications, and hematopoietic growth factors are not to be administered prophylactically prior to initiation of study treatment.

Patients requiring treatment for toxicities or for co-existent conditions may be treated as clinically indicated. Planned use of other medications should be discussed with the principal investigator.

Premedication with antihistamines may be administered for the second and subsequent atezolizumab infusions only, at the discretion of the investigator.

Patients who experience infusion-associated symptoms may be treated symptomatically with acetaminophen, ibuprofen, diphenhydramine, and/or H2-receptor antagonists (e.g., famotidine, cimetidine), or equivalent medications per local standard practice. Serious infusion-associated events manifested by dyspnea, hypotension, wheezing, bronchospasm, tachycardia, reduced oxygen saturation, or respiratory distress should be managed with supportive therapies as clinically indicated (e.g., supplemental oxygen and β2-adrenergic agonists; Section 9.2).

Prohibited Therapy

Use of the following concomitant therapies is prohibited during neoadjuvant treatment as described below:

Prohibited therapies applicable to all patients

Concomitant therapy intended for the treatment of cancer (including, but not limited to, chemotherapy, hormonal therapy, immunotherapy, radiotherapy for palliative or other purposes, and herbal therapy), whether health authority–approved or experimental, is prohibited during study treatment until disease progression is documented and the patient has discontinued study treatment.

Investigational therapy is prohibited within 42 days prior to initiation of study treatment and during the study.

Live, attenuated vaccines (e.g., FluMist®) are prohibited within 4 weeks prior to initiation of study treatment, during study treatment, for 90 days after the final dose of tiragolumab, and for 5 months after the final dose of atezolizumab.

Patients being treated with chemotherapy (i.e., carboplatin, cisplatin, pemetrexed, paclitaxel, or gemcitabine) should not receive live vaccines.

Systemic immunostimulatory agents (including, but not limited to, interferons and IL-2) are prohibited within 4 weeks or 5 drug-elimination half-lives (whichever is longer) prior to initiation of study treatment and during study treatment because these agents could potentially increase the risk for autoimmune conditions when given in combination with atezolizumab and tiragolumab.

Systemic immunosuppressive medications (including, but not limited to, cyclophosphamide, azathioprine, methotrexate, and thalidomide) during study treatment because these agents could potentially alter the efficacy and safety of study treatments.

Prohibited therapies applicable to patients receiving atezolizumab:

Live, attenuated vaccines (e.g., FluMist®) are prohibited within 4 weeks prior to initiation of atezolizumab, during atezolizumab treatment, and for 5 months after the last dose of atezolizumab.

Systemic immunostimulatory agents (including, but not limited to, interferons and IL-2) are prohibited within 4 weeks or five half-lives of the drug, whichever is longer, prior to initiation of atezolizumab and during atezolizumab treatment because these agents could potentially increase the risk for autoimmune conditions when given in combination with atezolizumab.

Systemic immunosuppressive medications (including, but not limited to, cyclophosphamide, azathioprine, methotrexate, and thalidomide) are prohibited during atezolizumab treatment because these agents could potentially alter the efficacy and safety of atezolizumab. However, systemic corticosteroids can be given as premedication to patients for whom CT scans with contrast are contraindicated (i.e., patients with contrast allergy or impaired renal clearance).

Prohibited therapies applicable to patients receiving cobimetinib or vemurafenib:

St. John's wort is prohibited during cobimetinib and vemurafenib treatment and for 2 weeks after the last dose of cobimetinib or vemurafenib (whichever is later).

Patients who require the use of any of the agents listed above will be discontinued from study treatment (unless use is agreed upon in consultation with the principal investigator) and followed for safety outcomes for 30 days after their last dose of study treatment or until they receive another anti-cancer therapy, whichever occurs first.

Cautionary Medications

Systemic corticosteroids and TNF-α inhibitors may attenuate potential beneficial immunologic effects of treatment with atezolizumab. For treatment of vemurafenib-related adverse events (other than hepatotoxicity as outlined in Section 8), systemic corticosteroids may be administered at the lowest effective dose and for the shortest possible duration necessary. If feasible, alternatives to these agents should be considered. Topical corticosteroids are allowed for suspected cutaneous autoimmune reactions. Megestrol may be administered as an appetite stimulant during the study. In situations in which systemic corticosteroids or TNF-α inhibitors would be routinely administered, alternatives, including antihistamines, should be considered. If the alternatives are not feasible, systemic corticosteroids and TNF-α inhibitors may be administered at the discretion of the investigator.

Systemic corticosteroids are recommended, at the discretion of the investigator, for the treatment of specific adverse events when associated with atezolizumab therapy (see Section 8).

Medications Given with Precaution due to Effects Related to Cytochrome P450 Enzymes

Cautionary therapies applicable to patients receiving cobimetinib

Concomitant use of strong and moderate inhibitors of CYP3A (e.g., clarithromycin, itraconazole, ketoconazole, posaconazole, telithromycin, and voriconazole) should be avoided during cobimetinib treatment because cobimetinib is a sensitive substrate of CYP3A and exposures will be increased in presence of these agents (approximately 7-fold increase in presence of itraconazole in healthy subjects).

Strong and moderate CYP3A inducers (e.g., rifampin, phenytoin, carbamazepine, and phenobarbital) should be avoided during cobimetinib treatment because they increase the metabolism of cobimetinib. Strong inducers of CYP3A4 should be avoided, or selection of an alternate concomitant medicinal product with no or minimal potential to induce CYP3A4 should be considered.

Cautionary therapies applicable to patients receiving vemurafenib

Vemurafenib is a moderate CYP1A2 inhibitor and a CYP3A4 inducer. Concomitant use of vemurafenib with agents with a narrow therapeutic window that are metabolized by CYP1A2 and CYP3A4 is not recommended. If co-administration cannot be avoided, exercise caution, as vemurafenib may increase plasma exposure of CYP1A2 substrate drugs and decrease plasma exposure of CYP3A4 substrate drugs. Dose reduction of the concomitant CYP1A2 substrate drug may be considered, if clinically indicated.

In two drug-drug interaction studies, vemurafenib increased the exposure of a single, oral dose of digoxin. An approximate 1.8- and 1.5-fold increase in digoxin area under the concentration-time curve (AUC) from Time 0 to the last measurable concentration and maximum concentration, respectively, was observed In Study GO28394. Digoxin and other p-glycoprotein substrates with a narrow therapeutic window are prohibited within 7 days prior to initiation of vemurafenib, during vemurafenib treatment, and for 30 days after the last dose of vemurafenib.

Warfarin and vemurafenib should be co-administered with caution, as vemurafenib has been shown to increase the AUC of S-warfarin, a CYP2C9 substrate. If warfarin is given, additional INR monitoring should be considered.

In vitro data have demonstrated that vemurafenib is a moderate inhibitor of CYP2C8. CYP2C8 substrates with a narrow therapeutic window should be used with caution, as their concentration may be increased when co-administered with vemurafenib.

In vitro data have demonstrated that vemurafenib is a substrate of CYP3A4. Thus, strong CYP3A4 inhibitors (e.g., ketoconazole, itraconazole, clarithromycin, atazanavir, nefazodone, saquinavir, telithromycin, ritonavir, indinavir, nelfainavir, voriconazole) and inducers (e.g., phenytoin, carbamazepine, rifampin, rifabutin, rifapentine, phenobarbital) should be used with caution when co-administered with vemurafenib

****The above-listed cautionary medications are not necessarily comprehensive. The investigator should consult the prescribing information for any concomitant medication when determining whether it can be safely co-administered with study treatment. In addition, the investigator should contact the principal investigator if questions arise regarding medications not listed above.

Medications Affecting the QT Interval

Caution should be exercised when the study drugs are co-administered with drugs that cause QT prolongation or cardiac arrhythmia, especially in patients with a preexisting cardiac disease or ECG abnormality that may predispose them to cardiac dysrhythmia. Investigators should closely monitor patients who are on medications or supplements that may affect the QT interval. Alternative treatment

options for medications known to affect QT interval should be discussed with each patient prior to their randomization in this study. Additional information is available at the following Internet site:

https://crediblemeds.org/healthcare-providers/

The effect of vemurafenib 960 mg BID on QT interval was evaluated in a multicenter, open-label, single-arm study in 132 previously treated patients with BRAFV600 mutation–positive metastatic melanoma (Study NP22657). No large changes from baseline in mean QTc interval (i.e., ≥20 ms) were detected in the study. However, vemurafenib is associated with concentration-dependent QT prolongation.

Although nonclinical studies showed a low potential for QT-interval prolongation with cobimetinib, no additive effect on QT-interval prolongation was observed when patients were treated with cobimetinib in combination with vemurafenib in Study GO28141. A combined cobimetinib and vemurafenib concentration-QTcF model (for which 433 patients contributed 1031 cobimetinib and vemurafenib observations) provided no evidence that cobimetinib prolonged QTcF interval when co-administered with vemurafenib.

Herbal Therapies

Concomitant use of herbal therapies is not recommended because their pharmacokinetics, safety profiles, and potential drug-drug interactions are generally unknown. However, herbal therapies not intended for the treatment of cancer may be used during the study at the discretion of the investigator.

Safety Plan

The safety plan for patients in this study is based on clinical experience with atezolizumab, cobimetinib, and vemurafenib in completed and ongoing studies.

Measures will be taken to ensure the safety of patients participating in this study, including the use of stringent inclusion and exclusion criteria and close monitoring of patients during the study. Administration of atezolizumab will be performed in a monitored setting in which there is immediate access to trained personnel and adequate equipment and medicine to manage potentially serious reactions. Adverse events will be reported as described in Section 10.0. In addition to the oversight provided by the principal investigator and drug safety personnel for this trial, an iDMC will monitor and evaluate patient safety throughout the study.

Risks Associated with Atezolizumab

Refer to the Atezolizumab Investigator's Brochure for a detailed description of anticipated safety risks for atezolizumab.

Systemic immune activation is a rare condition characterized by an excessive immune response. Given the mechanism of action of atezolizumab, systemic immune activation is considered a potential risk when given in combination with other immunomodulating agents. Systemic immune activation should be included in the differential diagnosis for patients who, in the absence of an alternative etiology, develop a sepsis-like syndrome after administration of atezolizumab, and the initial evaluation should include the following:

CBC with peripheral smear

PT, PTT, fibrinogen, and D-dimer

Ferritin

Triglycerides

AST, ALT, and total bilirubin

LDH

Complete neurologic and abdominal examination (assess for hepatosplenomegaly)

If systemic immune activation is still suspected after the initial evaluation, contact the Medical Monitor for additional recommendations.

Guidelines for management of patients who develop specific adverse events associated with atezolizumab are provided in Sections 8 and 9.

Risks Associated with Tiragolumab

IRR is an identified risk of tiragolumab. Although clinical evaluation of tiragolumab is limited and not all risks are known, as an antagonist of TIGIT, tiragolumab is anticipated to enhance T-cell and NK-cell proliferation, survival, and function. Therefore, tiragolumab may increase the risk of autoimmune inflammation (also described as immune-mediated adverse events). In addition, owing to the intact F_c-effector function of tiragolumab, lymphopenia by means of antibody dependent cell cytotoxicity (ADCC) is a theoretical risk.

Refer to the Tiragolumab Investigator's Brochure for a detailed description of anticipated safety risks for tiragolumab.

Infusion-Related Reactions

Because tiragolumab is a therapeutic mAb and targets tumor-infiltrating immune cells, IRRs associated with hypersensitivity reactions, target-mediated cytokine release, and/or emergent anti-drug antibodies (ADAs) may occur. Clinical signs and symptoms of such reactions may include rigors, chills, wheezing, pruritus, flushing, rash, hypotension, hypoxemia, and fever.

IRRs have been reported in patients treated with tiragolumab with or without atezolizumab. The majority of events were mild to moderate and manageable.

To minimize the risk and sequelae of IRRs, the initial dose of tiragolumab will be administered over 60 minutes followed by a 60-minute observation period, and subsequent infusions as well as observation times may be shortened only if the preceding infusion was well tolerated. All infusions will be administered in an appropriate medical setting.

Refer to Section 4.3.2 for detailed guidance on administration of tiragolumab in this study. Refer to Appendix 10 for guidance on anaphylaxis precautions, and Appendix 12 for guidance on management of IRRs and risks associated with tiragolumab.

Immune-Mediated Adverse Events

Nonclinical models have suggested a role of TIGIT signaling interruption in autoimmunity. In a knockout model ($\mathit{TIGIT}^{\square/\square}$), loss of TIGIT signaling resulted in hyperproliferative T-cell responses and exacerbation of experimental autoimmune encephalitis (EAE). $\mathit{TIGIT}^{\square/\square}$ and wild-type B6 mice were immunized with myelin oligodendrocyte glycoprotein peptide for induction of EAE using suboptimal doses. In contrast to the wild-type B6 mice, the majority of the $\mathit{TIGIT}^{\square/\square}$ mice developed severe EAE (Joller et al. 2011).

Clinical experience with therapeutic agents intended to enhance anti-tumor T-cell responses has demonstrated that development of autoimmune inflammatory conditions is a general risk and may therefore be considered a potential risk of tiragolumab. Such immune-mediated adverse events have been described for virtually all organ systems and include, but are not limited to, colitis, hepatitis, pneumonitis, endocrinopathies, ocular toxicity, pancreatic toxicity, neurologic toxicity, myocarditis, and rash. Rash and hypothyroidism have been reported in patients treated with tiragolumab, with or without atezolizumab.

Management guidelines for individual suspected immune-mediated adverse events are provided in Section 9

Lymphopenia

Given the IgG1 backbone of tiragolumab with intact Fc-effector function, ADCC mediated reduction in lymphocyte count is a potential risk. In a repeat-dose toxicity study in cynomolgus monkeys, however, there were no tiragolumab-related decreases in overall lymphocyte counts.

Transient decreases in lymphocyte count without clinical sequelae have been observed in patients treated with tiragolumab, with or without atezolizumab.

Owing to this potential risk of tiragolumab to induce lymphopenia, CBCs will be monitored regularly during the study (see Appendix 1).

Risks Associated with the Combination of Atezolizumab and Tiragolumab

Based on results from nonclinical and/or clinical studies with each molecule as a single agent, clinical data from Studies GO30103 and GO40290 with atezolizumab plus tiragolumab, and data from molecules with similar mechanisms of action, there is a potential for overlapping toxicity in patients treated with atezolizumab plus tiragolumab. Because the expected pharmacologic activity of these two molecules is to increase adaptive T-cell immune responses, there is the possibility of heightened immune responses.

Based on the mechanism of action of atezolizumab and tiragolumab, immune-mediated adverse events are potential overlapping toxicities associated with combination use of tiragolumab plus atezolizumab.

Based on clinical experience to date, it is anticipated that immune-mediated adverse events following treatment with atezolizumab and tiragolumab will be amenable to monitoring and manageable in the setting of this combination study. The extensive experience with immune CPIs to date has been incorporated into the design and safety management plan (see Section 5.1) in order to reduce the potential risks to participating patients.

Risks Associated with Cobimetinib

The following adverse events are classified as identified risks associated with cobimetinib: serous retinopathy, left ventricular dysfunction, photosensitivity (when administered with vemurafenib), severe hemorrhage, rhabdomyolysis, and pneumonitis. The following adverse events are classified as potential risks for cobimetinib: severe hepatotoxicity (Grade ≥3), impaired female fertility, and teratogenicity and developmental toxicity. In addition, there is the possibility of drug-drug interactions in patients treated with cobimetinib (see above sections of Appendix III for details). Refer to Section 6 of the cobimetinib Investigator's Brochure for a detailed description of all anticipated risks for cobimetinib.

Serous Retinopathy

Serous retinopathy (fluid accumulation within the layers of the retina) has been observed in patients treated with MEK inhibitors, including cobimetinib (Flaherty et al. 2012). Manifestations of serous retinopathy include visual disturbances, findings of retinal detachment, and retinopathy. Serous retinopathy events may also be asymptomatic.

To address serous retinopathy with cobimetinib treatment, all patients are required to undergo a baseline ophthalmologic examination to evaluate for risk factors for neurosensory retinal detachment. Patients will also undergo complete ophthalmologic examinations at specified timepoints throughout the study and as clinically indicated if a patient notes any visual disturbances.

Guidelines for management of patients who develop serous retinopathy are provided in Sections 8 and 9.

Left Ventricular Dysfunction

Decrease from baseline in left ventricular ejection fraction has been reported in patients receiving cobimetinib. Decreased left ventricular ejection fraction may be symptomatic or asymptomatic.

All patients will undergo evaluation of left ventricular ejection fraction, either by echocardiography or multigated acquisition scan at baseline, at specified timepoints during treatment, at the end of treatment, and as clinically indicated.

Guidelines for management of patients who have decreases in left ventricular ejection fraction are provided in Sections 8 and 9.

Photosensitivity (When Administered with Vemurafenib)

No evidence of photosensitivity has been observed with cobimetinib as a single agent. However, photosensitivity has been observed when cobimetinib was given in combination with vemurafenib.

Guidelines for management of patients who develop photosensitivity are provided in Sections 8 and 9.

Pneumonitis

Events of pneumonitis have been reported in cobimetinib clinical studies. Most reported events were reported as non-serious and a lower severity (grade).

Guidelines for management of patients who develop pulmonary events (including pneumonitis) are provided in Sections 8 and 9.

Rhabdomyolysis

Elevations in CPK have been observed in patients who received cobimetinib monotherapy as well as when administered with other agents. The majority of CPK elevations reported were asymptomatic, non-serious, and resolved with or without study drug interruption. One event of rhabdomyolysis was reported in the Phase III study GO28141, and rhabdomyolysis has been reported in postmarketing experience. CPK will be monitored at baseline and monthly during treatment or as clinically indicated.

Guidelines for management of patients who develop CPK elevations or rhabdomyolysis are provided in Sections 8 and 9.

Hemorrhage

Hemorrhage, including major hemorrhages defined as symptomatic bleeding in a critical area or organ, can occur with Cotellic. In clinical studies with cobimetinib, events of cerebral hemorrhage, gastrointestinal tract hemorrhage, reproductive tract hemorrhage, and hematuria, have been reported.

Caution should be used in patients with additional risk factors for bleeding, such as brain metastases, and/or in patients that use concomitant medications that increase the risk of bleeding (including antiplatelet or anticoagulant therapy).

Instructions for dose modification for hemorrhage events are included in Sections 8 and 9.

Severe Hepatotoxicity (Grade ≥ 3)

Liver laboratory test abnormalities, including increases in ALT, AST, and alkaline phosphatase, have been reported as adverse events and serious adverse events in patients treated with cobimetinib and vemurafenib. Generally, elevations in liver laboratory tests have been managed effectively with dose modifications.

Guidelines for management of patients who develop elevations in ALT, AST, and/or bilirubin are provided in Sections 8 and 9.

Impaired Female Fertility

Results from nonclinical studies indicate that there is a potential for effects on female fertility. While no dedicated fertility studies have been conducted with cobimetinib in animals, degenerative changes were observed in reproductive tissues of dogs. These changes were reversible upon discontinuation of cobimetinib.

Teratogenicity and Developmental Toxicity

There are no data regarding the use of cobimetinib in pregnant women. When cobimetinib was administered to pregnant rats, cobimetinib caused embryolethality and fetal malformations of the great vessels and skull at similar systemic exposures to those observed in patients administered the 60 mg dose. Therefore, teratogenicity and developmental toxicity is a potential risk for cobimetinib, and cobimetinib use is not recommended during pregnancy.

Risks Associated with Vemurafenib

The following adverse events are classified as identified risks associated with vemurafenib: cutaneous squamous cell carcinoma, new primary melanoma, progression of RAS-mutant malignancy, photosensitivity and sunburn, liver injury, QT prolongation, hypersensitivity and severe cutaneous reactions, uveitis, VIIth nerve paralysis, radiation recall and radiation sensitization, and acute kidney injury. The following adverse events are classified as potential risks for vemurafenib: non-cutaneous squamous cell carcinoma, bone marrow toxicity, drug-drug interaction, second primary malignancy, gastrointestinal polyps, and retinal vein occlusion. In addition, neutropenia, pancreatitis, and Dupuytren's contracture/plantar fascial fibromatosis have been identified as adverse drug reactions in the postmarketing setting. Descriptions of identified risks and selected other risks are provided below. Refer to Section 6 of the vemurafenib Investigator's Brochure for a detailed description of all identified and potential risks for vemurafenib, including adverse drug reactions in the postmarketing setting.

Cutaneous Squamous Cell Carcinoma and New Primary Melanoma

Cases of cutaneous squamous cell carcinoma, which include those classified as keratoacanthomas or mixed keratoacanthoma subtype, have been reported in patients treated with vemurafenib, usually early in the course of treatment. Potential risk factors associated with cutaneous squamous cell carcinoma in three vemurafenib clinical trials included age (≥65 years), prior skin cancer, and chronic sun exposure. Cases of new primary malignant melanoma have also been reported in patients treated with vemurafenib. Cutaneous squamous cell carcinoma and new primary malignant melanoma were managed with excision, and patients were able to continue treatment without dose adjustment.

All new primary neoplasms, including new primary melanoma, will be reported until 6 months after the last dose of vemurafenib.

Guidelines for management of patients who develop new skin lesions are provided in Sections 8 and 9.

Non-Cutaneous Squamous Cell Carcinoma

Cases of squamous cell carcinoma of the head and neck have been reported in patients treated with vemurafenib.

RAS-Mutant Malignancies

On the basis of its mechanism of action, vemurafenib may cause progression of cancers associated with RAS mutations. In addition, progression of pre-existing RAS-mutant malignancies (chronic myelomonocytic leukemia, pancreatic cancer) have been reported in patients treated with vemurafenib. Vemurafenib should be used with caution in patients with a prior cancer associated with RAS mutation.

Photosensitivity and Sunburn

Mild to severe skin photosensitivity has been reported in patients treated with vemurafenib. All patients should be advised to minimize sun exposure, wear protective clothing, and use a broad-spectrum

ultraviolet A/ultraviolet B sunscreen and lip balm (SPF≥30), reapplied every 2 to 3 hours, when outdoors during vemurafenib treatment and for at least 5-10 days after discontinuing vemurafenib.

Guidelines for management of patients who develop photosensitivity are provided in Sections 8 and 9.

Liver Injury

Liver injury, including cases of severe liver injury, have been reported in patients treated with vemurafenib. Alkaline phosphatase, ALT, AST, and bilirubin will be monitored at specified timepoints throughout the study.

Guidelines for management of patients who develop elevations in ALT, AST, and/or bilirubin are provided in Sections 8 and 9.

QT Prolongation

Exposure-dependent QT prolongation was observed in an uncontrolled, open-label Phase II QT substudy in patients with metastatic melanoma. QT prolongation may lead to an increased risk of ventricular arrhythmias, including torsades de pointes.

Patients with a history of congenital long QT syndrome, QTc interval corrected using Fridericia’s method ≥450 ms, or uncorrectable abnormalities in serum electrolytes will be excluded from study inclusion. ECG and electrolytes, including potassium, magnesium, and calcium, will be monitored throughout the study. In addition, investigators should closely monitor patients who are on medications or supplements that may affect the QT interval. Alternative treatment options for medications known to affect QT interval should be discussed with each patient prior to their randomization in this study.

Guidelines for management of patients who develop QT prolongation are provided in Sections 8 and 9.

Hypersensitivity and Severe Cutaneous Reactions

Serious hypersensitivity reactions, including anaphylaxis, have been reported in association with vemurafenib and upon re-initiation of treatment. Severe hypersensitivity reactions included generalized rash and erythema or hypotension. Drug reaction with eosinophilia and systemic symptoms has been reported in association with vemurafenib in the postmarketing setting. Severe dermatologic reactions have been reported in patients receiving vemurafenib, including cases of Stevens-Johnson syndrome and toxic epidermal necrolysis.

Guidelines for management of patients who develop hypersensitivity reactions and dermatologic events are provided in Sections 8 and 9.

Uveitis and Retinal Vein Occlusion

Serious ophthalmologic reactions, including uveitis, have been reported in patients treated with vemurafenib. Retinal vein occlusion has been observed and is a potential risk.

Patients will under ophthalmologic examinations at specified timepoints during the study (see Section 4). Patients will be evaluated at screening for risk factors for neurosensory retinal detachment, retinal vein occlusion, or neovascular macular degeneration. Risk factors for retinal vein occlusion include elevated serum cholesterol, hypertriglyceridemia, hyperglycemia, hypertension, and glaucoma. Patients with such conditions will be excluded from the study as detailed in Section 3.

Guidelines for management of patients who develop uveitis or retinal vein occlusion are provided in Sections 8 and 9.

VIIth Nerve Paralysis

Cases of VIIth nerve paralysis have been observed in patients treated with vemurafenib. In clinical trials, these events resolved without sequelae.

Radiation Recall and Radiation Sensitization

An adverse drug reaction of potentiation of radiation treatment toxicity has been identified in patients treated with radiation prior to, during, or subsequent to vemurafenib treatment. The nature and severity were evaluated as worse than expected for the normal tissue tolerance to therapeutic radiation. The reaction was seen in the skin, esophagus, lung, liver, rectum, and urinary bladder. Most cases were cutaneous in nature, but some cases involving visceral organs had fatal outcomes. Vemurafenib should be used with caution when given concomitantly or sequentially with radiation treatment.

Radiotherapy is not permitted during the study.

Acute Kidney Injury and Renal Function Alterations

Acute kidney injury, including interstitial nephritis, has been observed in patients treated with vemurafenib. The majority of these cases have been characterized by mild to moderate increases in serum creatinine (some observed in the setting of dehydration events), with recovery after dose modification.

Serum creatinine will be monitored throughout the study. Vemurafenib acute kidney injury dose modification guidelines should be utilized when applicable and it is recommended to routinely monitor serum creatinine levels in all patients undergoing vemurafenib therapy.

Drug-Drug Interaction

Drug-drug interactions are a potential risk for vemurafenib. Prohibited therapies and food and cautionary therapies are described above in Appendix III.

Gastrointestinal Polyps

Rare cases of colonic polyps have been reported in patients treated with vemurafenib for 2 or more years while enrolled in a clinical study (Chapman et al. 2012).

Neutropenia

Neutropenia has been identified as an uncommon adverse drug reaction associated with the use of vemurafenib, typically occurring during the first 6-12 weeks of treatment. It appears to be reversible usually within 2 weeks, with temporary interruption, dose reduction, or discontinuation of vemurafenib, and in some cases has been managed with granulocyte colony-stimulating factor.

Pancreatitis

Pancreatitis has been identified as an uncommon adverse drug reaction in patients being treated with vemurafenib. The clinical presentation in terms of severity, mild to moderate, was consistent with the clinical picture of drug-induced pancreatitis (Lankisch et al.1995).

The Sponsor recommends that serum amylase and lipase testing be conducted as part of the workup of any suspected case of pancreatitis in addition to other appropriate testing (e.g., abdomen computed tomography scan).

Guidelines for management of patients who develop pancreatitis are provided in Sections 8 and 9.

Potential Risks Associated with Combination Use of Atezolizumab, Cobimetinib, and Vemurafenib

The following adverse events are common toxicities observed in ≥ 10% of patients treated with atezolizumab, vemurafenib, or cobimetinib monotherapy, or in patients treated with vemurafenib in combination with cobimetinib: anemia, chills, decreased appetite, headache, nausea, peripheral edema, vomiting, fatigue, arthralgia, pyrexia, diarrhea, and rash. In addition, the following represent potential overlapping toxicities: gastrointestinal toxicity, liver laboratory abnormalities and hepatotoxicity, dermatologic toxicity, pancreatitis, nephritis or increased creatinine, hyperglycemia, ocular events, and pneumonitis. Descriptions of potential risks with combination treatment are provided below.

Gastrointestinal Toxicity

Immune-mediated colitis has been associated with the administration of atezolizumab. In addition, because diarrhea has been commonly observed with vemurafenib, cobimetinib, and cobi + vem, gastrointestinal reactions are a potential overlapping toxicity with this treatment combination. Diarrhea can frequently be managed with anti-diarrheal agents but can also progress to clinically significant dehydration and/or electrolyte imbalances with effects on other organs, possibly resulting in renal, hepatic, and/or cardiac failure. Patients should be instructed to promptly contact the investigators if they develop diarrhea. Investigators should treat diarrhea and intervene promptly for patients who appear to be at increased risk of developing significant dehydration, electrolyte imbalances, or multi-organ failure. Patients should receive maximum supportive care per institutional guidelines.

Guidelines for management of patients who develop gastrointestinal toxicity are provided in Sections 8 and 9.

Hepatic Toxicity

Liver laboratory abnormalities and hepatotoxicity have been observed with cobi + vem, vemurafenib monotherapy, and atezolizumab monotherapy. Generally, liver laboratory abnormalities following treatment with cobi + vem as well as vemurafenib monotherapy are manageable with dose modifications. Rare cases of immune-related hepatitis have been associated with the administration of atezolizumab. It appears that Grade ≥3 ALT or AST elevations may occur at a higher frequency with atezo + cobi + vem.

Eligible patients must have adequate liver function, as manifested by measurements of total bilirubin and hepatic transaminases. Patients with known liver disease that could increase susceptibility to or exacerbate the effect of any potential hepatotoxicity of study treatment, including those with active hepatitis B or active hepatitis C viral infection, are excluded from the study.

Liver function will be monitored throughout the study. Patients with right upper-quadrant abdominal pain and/or unexplained nausea or vomiting should have liver function tests performed immediately and reviewed before administration of the next dose of study drug. For patients with elevated liver-function tests, concurrent medication, viral hepatitis, and toxic or neoplastic etiologies should be considered and addressed, as appropriate.

Guidelines for management of patients who develop elevations in ALT, AST, and/or bilirubin are provided in Sections 8 and 9.

Dermatologic Toxicity

Treatment-emergent rash has been reported with single-agent atezolizumab. The majority of these cases were mild in severity and self-limited, with or without pruritus. Because rash has been observed with vemurafenib and cobi + vem, this is a potentially overlapping toxicity and should be closely monitored. A dermatologist should evaluate persistent and/or severe rash or pruritus. A biopsy should be considered unless contraindicated.

Guidelines for management of patients who develop dermatologic toxicity are provided in Sections 8 and 9.

Pancreatic Events

Symptoms of abdominal pain associated with elevations of amylase and lipase, suggestive of pancreatitis, have been associated with the administration of atezolizumab. Pancreatitis is an adverse drug reaction for vemurafenib and could therefore represent an overlapping toxicity. The differential diagnosis of acute abdominal pain should include pancreatitis. Appropriate work-up should include an evaluation for ductal obstruction, as well as serum amylase and lipase tests.

Guidelines for management of patients who develop pancreatic events are provided in Sections 8 and 9.

Renal Events

Renal failure, ranging from creatinine elevation to acute interstitial nephritis and acute tubular necrosis, has been observed with vemurafenib treatment. Nephritis is a rare potential risk for atezolizumab.
Creatinine will be monitored according to the schedule of activities during study treatment. Guidelines for management of patients who develop renal events are provided in Sections 8 and 9.

Hyperglycemia

Increased blood sugar has been rarely observed with cobimetinib treatment. Rare cases of immune related diabetes have been observed with atezolizumab treatment. Blood glucose will be assessed throughout the study. Patients with Grade ≥2 or symptomatic hyperglycemia (fasting) will be exclusionary. Guidelines for management of patients who develop hyperglycemia are provided in Sections 8 and 9.

Ocular Toxicity

Uveitis or episcleritis and other immune-mediated ocular disease may be associated with atezolizumab. Ophthalmologic reactions have been observed with vemurafenib (uveitis and retinal vein occlusion) and cobimetinib (serous retinopathy). Thus, this is a potential overlapping toxicity with this treatment combination.

Patients will under ophthalmologic examinations at specified timepoints during the study. An ophthalmologist should evaluate visual complaints.

Guidelines for management of patients who develop ocular toxicity are provided in Sections 8 and 9.

Pulmonary Toxicity

Dyspnea, cough, fatigue, hypoxia, pneumonitis, and pulmonary infiltrates have been associated with the administration of atezolizumab. Pneumonitis is an identified risk for cobimetinib and is therefore a potential overlapping toxicity with this treatment combination.

Patients will be assessed for pulmonary signs and symptoms throughout the study and will also have computed tomography scans of the chest performed at every tumor assessment. All pulmonary events should be thoroughly evaluated for other commonly reported etiologies such as pneumonia or other infection, lymphangitic carcinomatosis, pulmonary embolism, heart failure, chronic obstructive pulmonary disease, or pulmonary hypertension.

Guidelines for management of patients who develop pulmonary toxicity (including pneumonitis) are provided in Sections 8 and 9.

Management of Patients Who Experience Specific Adverse Events See Sections

8 and 9.
