# Supplementary material for: Neoadjuvant cobimetinib and atezolizumab with or without vemurafenib for high-risk operable Stage III melanoma: the Phase II NeoACTIVATE trial
Source: Nat Commun. 2024 Feb 16;15:1430. doi: 10.1038/s41467-024-45798-8 (PMC10873383; doi:10.1038/s41467-024-45798-8)
Supplement: Supplementary file 2 — Reporting Summary [file 41467_2024_45798_MOESM2_ESM.pdf]

nature portfolio

Corresponding author(s): Matthew S. Block

Last updated by author(s): Dec 18, 2023

Reporting Summary

Nature Portfolio wishes to improve the reproducibility of the work that we publish. This form provides structure for consistency and transparency in reporting. For further information on Nature Portfolio policies, see our Editorial Policies and the Editorial Policy Checklist.

Statistics

For all statistical analyses, confirm that the following items are present in the figure legend, table legend, main text, or Methods section.

n/a	Confirmed	
☐	☒	The exact sample size (*n*) for each experimental group/condition, given as a discrete number and unit of measurement
☐	☒	A statement on whether measurements were taken from distinct samples or whether the same sample was measured repeatedly
☐	☒	The statistical test(s) used AND whether they are one- or two-sided<br>*Only common tests should be described solely by name; describe more complex techniques in the Methods section.*
☐	☒	A description of all covariates tested
☐	☒	A description of any assumptions or corrections, such as tests of normality and adjustment for multiple comparisons
☐	☒	A full description of the statistical parameters including central tendency (e.g. means) or other basic estimates (e.g. regression coefficient) AND variation (e.g. standard deviation) or associated estimates of uncertainty (e.g. confidence intervals)
☐	☒	For null hypothesis testing, the test statistic (e.g. *F*, *t*, *r*) with confidence intervals, effect sizes, degrees of freedom and *P* value noted<br>*Give P values as exact values whenever suitable.*
☒	☐	For Bayesian analysis, information on the choice of priors and Markov chain Monte Carlo settings
☒	☐	For hierarchical and complex designs, identification of the appropriate level for tests and full reporting of outcomes
☒	☐	Estimates of effect sizes (e.g. Cohen's *d*, Pearson's *r*), indicating how they were calculated

Our web collection on statistics for biologists contains articles on many of the points above.

Software and code

Policy information about availability of computer code

Data collection	The clinical data were collected using electronic case report forms (eCRF) with iMedidata Rave software at Mayo Clinic Rochester and University of Minnesota. Flow cytometry data were collected on a CytoFLEX LX (Beckman Coulter, Atlanta, GA). CyTOF data were collected as .FCS files using the Cytof software (Version 6.7.1014). After acquisition intra file signal drift was normalized to the acquired calibration bead signal using the CyTOF software.
Data analysis	Statistical analyses were performed using SAS software version 9.4. Flow cytometry analysis was performed with the FlowJo Software 10.4 (Tree Star, Palo Alto, CA). Normalized .FCS data were uploaded to the Astrolabe Cytometry Plateform (Astrolabe Diagnostics, Inc) where transformation, cleaning (doublets, debris), labeling, and unsupervised clustering were done. The resulting processed data were further analyzed use R software version 4.1.1.

For manuscripts utilizing custom algorithms or software that are central to the research but not yet described in published literature, software must be made available to editors and reviewers. We strongly encourage code deposition in a community repository (e.g. GitHub). See the Nature Portfolio guidelines for submitting code & software for further information.

Data

Policy information about availability of data

All manuscripts must include a data availability statement. This statement should provide the following information, where applicable:

- Accession codes, unique identifiers, or web links for publicly available datasets
- A description of any restrictions on data availability
- For clinical datasets or third party data, please ensure that the statement adheres to our policy

De-identified data are available with scientific approval of the study team under restricted access. These data will be provided to scientific investigators for research purposes. Requests will be reviewed by the Institutional Review Board and subject to a Data Use Agreement. Details on acceptable methods and duration of data transfer will be determined by institutional policies based on which data are requested and for what research purposes. The remaining data can be found in the Article, Supplementary and Source Data files.

Research involving human participants, their data, or biological material

Policy information about studies with human participants or human data. See also policy information about sex, gender (identity/presentation), and sexual orientation and race, ethnicity and racism.

Reporting on sex and gender	The ratio of female to male patients enrolled in this trial is 19:11, which is slightly higher than is reported among melanoma patients of median age 59 years, as melanoma is generally more common in females under age 50 but shifts to a male preponderance at older ages.
Reporting on race, ethnicity, or other socially relevant groupings	All patients enrolled in this study were Caucasian, which is reflective of the demographics of melanoma within the United States.
Population characteristics	The median patient age was 59, and 90% had an ECOG performance of 0. The majority of patients had more than one clinically evident involved lymph node at presentation prior to neoadjuvant therapy. Further details are described in the manuscript text and summarized in Table 1.
Recruitment	Patients were screened for eligibility prior to their consultation visit at Mayo Clinic Rochester and University of Minnesota. All eligible patients were offered trial participation. Comparison of the screening log with the enrolled patients showed similar patient characteristics in both groups. The primary reasons for non-enrollment among screened patients were prior excisional biopsy of the involved lymph node, abnormal electrocardiogram characteristics, and prior malignancies.
Ethics oversight	The study was conducted in accordance with Good Clinical Practice guidelines after approval of the institutional review boards at both Mayo Clinic and University of Minnesota and with oversight by an independent data safety monitoring board. The study design and conduct complied with all relevant regulations regarding the use of human study participants and was conducted in accordance with the criteria set by the Declaration of Helsinki.

Note that full information on the approval of the study protocol must also be provided in the manuscript.

Field-specific reporting

Please select the one below that is the best fit for your research. If you are not sure, read the appropriate sections before making your selection.

[x] Life sciences [ ] Behavioural & social sciences [ ] Ecological, evolutionary & environmental sciences

For a reference copy of the document with all sections, see nature.com/documents/nr-reporting-summary-flat.pdf

Life sciences study design

All studies must disclose on these points even when the disclosure is negative.

Sample size	A sample size of 15 patients per cohort was chosen so that the maximum half width of the 90% binomial confidence for the pCR rate would be +/- 21.2%.
Data exclusions	There were none.
Replication	Data were not able to be replicated, as this is a study involving human subjects, and therefore, the data generated thereof are unique to each study participant.
Randomization	No randomization occurred. Allocation to treatment arm was based on BRAF mutation status. Sample size precludes additional controls for co-variates; patient characteristics were similar across both cohorts.
Blinding	No blinding was used, as is customary for a non-randomized trial.

Reporting for specific materials, systems and methods

We require information from authors about some types of materials, experimental systems and methods used in many studies. Here, indicate whether each material, system or method listed is relevant to your study. If you are not sure if a list item applies to your research, read the appropriate section before selecting a response.

Materials & experimental systems

n/a	Involved in the study
☐	☒ Antibodies
☒	☐ Eukaryotic cell lines
☒	☐ Palaeontology and archaeology
☒	☐ Animals and other organisms
☐	☒ Clinical data
☒	☐ Dual use research of concern
☒	☐ Plants

Methods

n/a	Involved in the study
☒	☐ ChIP-seq
☐	☒ Flow cytometry
☒	☐ MRI-based neuroimaging

Antibodies

Antibodies used

Name	Clone	Cat#	Concentration (dilution)	Company
Anti-Human CD8-PE-Cy7	RPA-T8	557746	1:200	BD Biosciences
Anti-human PD-1-BV510	EH12.2H7	329931	1:100	Biolegend
Anti-human CD11a-APC	HI111	301212	1:200	Biolegend
Anti-human CX3CR1-APC/Cy7	2A9.1	341616	1:100	Biolegend
Anti-human CD3-BV421	OKT3	317344	1:200	BD Biosciences
Anti-Bim-Rabbit mAb -PE	C34C5	12186	1:200	Cell Signaling
Anti-NKG7-Rabbit mAb-AF488	8H3/8K3		1:200	House made
CD45	HI30	3089003B	400	Standard BioTools
CD196/CCR6	G034E3	3141003A	200	Standard BioTools
CD19	HIB19	3142001B	400	Standard BioTools
CD127/IL-7Ra	A019D5	3143012B	200	Standard BioTools
CD38	HIT2	3144014B	200	Standard BioTools
CD11a	HI111	301223	400	Biolegend
IgD	IA6-2	3146005B	400	Standard BioTools
CD11c	Bu15	3147008B	400	Standard BioTools
CD16	3G8	3148004B	800	Standard BioTools
CD194/CCR4	L291h4	3149029A	200	Standard BioTools
LAG-3	11C3C65	3150030B	100	Standard BioTools
CD123/IL-3R	6H6	3151001B	200	Standard BioTools
TCRgd	11F2	3152008B	400	Standard BioTools
CD185/CXCR5	RF8B2	3153020B	200	Standard BioTools
CD3	UCHT1	3154003B	400	Standard BioTools
CD45RA	HI100	3155011B	400	Standard BioTools
PD-L1	29E.2A3	3156026B	100	Standard BioTools
CD27	L128	3158010B	400	Standard BioTools
Tim-3	F38-2E2	345019	100	Biolegend
CD28	CD28.2	3160003B	200	Standard BioTools
PD-1	EH12.2H7	329941	100	Biolegend
CD66b	80H3	3162023B	100	Standard BioTools
CD183/CXCR3	G025H7	3163004B	200	Standard BioTools
CD161	HP-3G10	3164009B	100	Standard BioTools
CD45RO	UCHL1	3165011B	200	Standard BioTools
CD24	ML5	3166007B	200	Standard BioTools
CD197/CCR7	G043H7	3167009A	200	Standard BioTools
CD8a	SK1	3168002B	800	Standard BioTools
CD25/IL-2R	2A3	3169003B	200	Standard BioTools
CTLA-4	14D3	3170005B	100	Standard BioTools
CD20	2H7	3171012B	800	Standard BioTools
CX3CR1	2A9-1	341602	100	Biolegend
HLA-DR	L243	3173005B	400	Standard BioTools
CD4	SK3	3174004B	800	Standard BioTools
CD14	M5E2	3175015B	200	Standard BioTools
CD56/NCAM	NCAM16.2	3176008B	200	Standard BioTools
TIGIT	MBSA43	3209013B	100	Standard BioTools

Validation

CyTOF Antibodies were validated in the following manuscript:
Bagwell CB, Hunsberger B, Hill B, Herbert D, Bray C, Selvanantham T, Li S, Villasboas JC, Pavelko K, Strausbauch M, Rahman A, Kelly G, Asgharzadeh S, Gomez-Cabrero A, Behbehani G, Chang H, Lyberger J, Montgomery R, Zhao Y, Inokuma M, Goldberger O, Stelzer G. Multi-site reproducibility of a human immunophenotyping assay in whole blood and peripheral blood mononuclear cells preparations using CyTOF technology coupled with Maxpar Pathsetter, an automated data analysis system. Cytometry B Clin Cytom. 2020 Mar;98 (2):146-160. doi: 10.1002/cyto.b.21858. Epub 2019 Nov 23. PMID: 31758746; PMCID: PMC7543682.
Validation for flow cytometry antibodies was provided in the following manuscripts:
Yan, Y., et al. CX3CR1 identifies PD-1 therapy-responsive CD8+ T cells that withstand chemotherapy during cancer chemoimmunotherapy. JCI Insight 3(2018).
Zhang, H., et al. Phase II Evaluation of Stereotactic Ablative Radiotherapy (SABR) and Immunity in (11)C-Choline-PET/CT-Identified Oligometastatic Castration-Resistant Prostate Cancer. Clin Cancer Res 27, 6376-6383 (2021).
Gicobi, J.K., et al. Salvage therapy expands highly cytotoxic and metabolically fit resilient CD8(+) T cells via ME1 up-regulation. Sci Adv 9, eadi2414 (2023).
Wen, T., et al. NKG7 Is a T-cell-Intrinsic Therapeutic Target for Improving Antitumor Cytotoxicity and Cancer Immunotherapy. Cancer Immunol Res 10, 162-181 (2022).

Clinical data

Policy information about clinical studies

All manuscripts should comply with the ICMJE guidelines for publication of clinical research and a completed CONSORT checklist must be included with all submissions.

Clinical trial registration	NCT03554083
Study protocol	Supplementary Note
Data collection	Collected using electronic case report forms (eCRF) with iMedidata Rave software at Mayo Clinic Rochester and University of Minnesota. Enrollment from 6/22/18-5/10/21. Data collection from 6/22/18-8/9/21.
Outcomes	Pathologic response is the primary endpoint of the neoadjuvant component of this study and is reported as percentage of viable tumor across the entire tumor bed as per suggested international guidelines (INMC). Pre-specified benchmarks for major pathologic response were defined as 50% (Cohort A) and 30% (Cohort B) based on best estimates at time of study activation when little data existed for neoadjuvant treatment of Stage III melanoma. Our secondary endpoint was adverse events during the neoadjuvant portion of the trial, as defined per CTCAE v.4.0. No pre-specified benchmark was defined for adverse events.

Flow Cytometry

Plots

Confirm that:

☒ The axis labels state the marker and fluorochrome used (e.g. CD4-FITC).

☒ The axis scales are clearly visible. Include numbers along axes only for bottom left plot of group (a 'group' is an analysis of identical markers).

☒ All plots are contour plots with outliers or pseudocolor plots.

☒ A numerical value for number of cells or percentage (with statistics) is provided.

Methodology

Sample preparation	Blood was obtained at the time of registration, after Cycles 1, 2, and 3 of neoadjuvant therapy, and after Cycle 4 (lymph node dissection). Blood was processed for viably cyropreserved peripheral blood mononuclear cells (PBMCS) and plasma per laboratory protocols.
Instrument	Data were collected on a CytoFLEX LX (Beckman Coulter, Atlanta, GA).
Software	FlowJo Software 10.4 (Tree Star, Palo Alto, CA).
Cell population abundance	The frequency of tumor-related T cells was defined as the number of CD8+/PD-1+/CD11c+ cells as a fraction of the total number of CD8+ cells. The frequency of effector CTLs was defined as the number of CD8+/PD-1+/CD11c+/NKG7+/CX3CR1+ cells as a fraction of the CD8+/PD-1+/CD11c+ cells. The frequency of pro-apoptotic cells was defined as the number of CD8+/PD-1+/CD11c+/Bim+ cells as a fraction of the CD8+/PD-1+/CD11c+ cells.
Gating strategy	Cells were gated as described within the references to the Methods section and Supplementary Information. In brief, the preliminary FSC/SSC gates were drawn to surround the relatively homogeneous population of lymphocytes within PBMCs. Fluorescent gates were designed to split bimodal (positive/negative) populations with maximum distinction.

☒ Tick this box to confirm that a figure exemplifying the gating strategy is provided in the Supplementary Information.
